# B Cell Lymphoma Immunotherapy Using TLR9-Targeted Oligonucleotide STAT3 Inhibitors

**DOI:** 10.1016/j.ymthe.2018.01.007

**Published:** 2018-01-17

**Authors:** Xingli Zhao, Zhuoran Zhang, Dayson Moreira, Yu-Lin Su, Haejung Won, Tomasz Adamus, Zhenyuan Dong, Yong Liang, Hongwei H. Yin, Piotr Swiderski, Raju K. Pillai, Larry Kwak, Stephen Forman, Marcin Kortylewski

**Affiliations:** 1 Department of Immuno-Oncology, Beckman Research Institute, City of Hope, Duarte, CA 91010, USA; 2 State Key Laboratory of Experimental Hematology, Institute of Hematology and Blood Diseases Hospital, Chinese Academy of Medical Science and Peking Union Medical College, Tianjin 300020, China; 3 Department of Hematology and Hematopoietic Cell Transplantation, Beckman Research Institute, City of Hope, Duarte, CA 91010, USA; 4 Toni Stephenson Lymphoma Center, Department of Hematology and Hematopoietic Cell Transplantation, Beckman Research Institute, City of Hope, Duarte, CA 91010, USA; 5 DNA/RNA Synthesis Core Facility, Beckman Research Institute, City of Hope, Duarte, CA 91010, USA; 6 Molecular Pathology, Beckman Research Institute, City of Hope, Duarte, CA 91010, USA

**Keywords:** immunotherapy, STAT3, TLR9, CpG oligonucleotides, lymphoma

## Abstract

Growing evidence links the aggressiveness of non-Hodgkin’s lymphoma, especially the activated B cell-like type diffuse large B cell lymphomas (ABC-DLBCLs) to Toll-like receptor 9 (TLR9)/MyD88 and STAT3 transcription factor signaling. Here, we describe a dual-function molecule consisting of a clinically relevant TLR9 agonist (CpG7909) and a STAT3 inhibitor in the form of a high-affinity decoy oligodeoxynucleotide (dODN). The CpG-STAT3dODN blocked STAT3 DNA binding and activity, thus reducing expression of downstream target genes, such as *MYC* and *BCL2L1*, in human and mouse lymphoma cells. We further demonstrated that injections (i.v.) of CpG-STAT3dODN inhibited growth of human OCI-Ly3 lymphoma in immunodeficient mice. Moreover, systemic CpG-STAT3dODN administration induced complete regression of the syngeneic A20 lymphoma, resulting in long-term survival of immunocompetent mice. Both TLR9 stimulation and concurrent STAT3 inhibition were critical for immune-mediated therapeutic effects, since neither CpG7909 alone nor CpG7909 co-injected with unconjugated STAT3dODN extended mouse survival. The CpG-STAT3dODN induced expression of genes critical to antigen-processing/presentation and Th1 cell activation while suppressing survival signaling. These effects resulted in the generation of lymphoma cell-specific CD8/CD4-dependent T cell immunity protecting mice from tumor rechallenge. Our results suggest that CpG-STAT3dODN as a systemic/local monotherapy or in combination with PD1 blockade can provide an opportunity for treating patients with B cell NHL.

## Graphical Abstract

**Figure:**
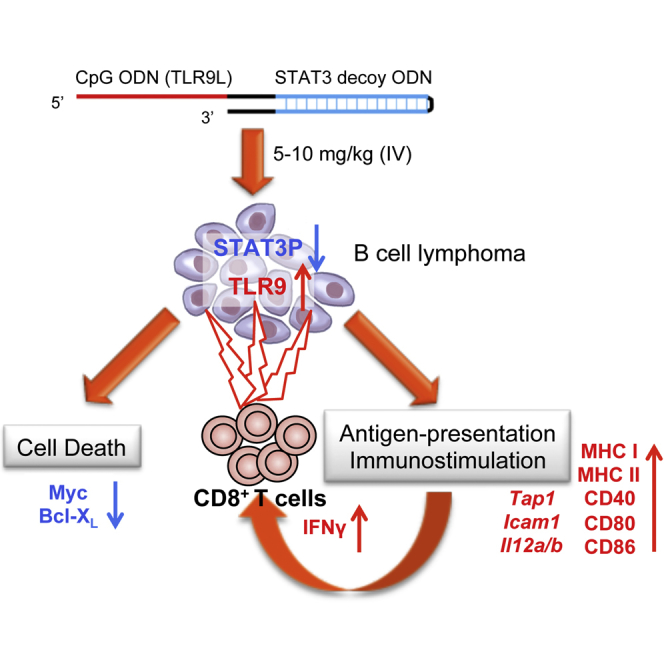

## Teaser

Zhao et al. describe a synthetic oligonucleotide-based strategy for therapy of disseminated B cell lymphomas. They demonstrate that combination of the TLR9 agonist (CpG) with the STAT3 decoy inhibitor into a single oligodeoxynucleotide conjugate generates two-pronged therapeutic effect by direct and T cell-mediated antitumor effects against B cell lymphoma cells *in vivo*.

## Introduction

Diffuse large B cell lymphoma (DLBCL) is the most prevalent, aggressive type of non-Hodgkin’s lymphoma (NHL).^1^, ^2^, ^3^ While chemo-immunotherapy improved outcome for advanced NHLs, a significant percentage of patients ultimately develops the progressive disease.^4^ The STAT3 transcription factor is frequently activated in NHL, regulating cell survival and proliferation, and has been associated with poor survival of patients with aggressive lymphoma.^5^, ^6^, ^7^, ^8^, ^9^ Mutations of upstream STAT3 regulators are common in DLBCL.^10^ STAT3 activation has been linked to autocrine/paracrine stimulation by interleukin (IL)-6 in the tumor microenvironment.^11^, ^12^ More universally, STAT3 is a central immune checkpoint regulator in cancer cells and tumor-associated immune cells, such as myeloid-derived suppressor cells (MDSCs) and tumor-associated macrophages (TAMs).^13^, ^14^, ^15^, ^16^ While an attractive target for cancer therapy,^17^, ^18^, ^19^ direct pharmacological inhibition of STAT3 proved difficult, and despite many attempts, there are still no US Food and Drug Administration (FDA)-approved small-molecule STAT3 inhibitors.^20^, ^21^ These challenges underscore the need for alternative strategies, such as oligonucleotide-based STAT3 inhibitors,^20^, ^22^ and new methods for targeted oligonucleotide delivery.^23^ To overcome these obstacles, we previously developed a strategy for the delivery of therapeutic oligonucleotides, such as *STAT3* small interfering RNA (siRNA), specifically to Toll-like receptor 9^+^ (TLR9^+^) immune cells, such as plasmacytoid dendritic cells (pDCs), B cells,^24^, ^25^ and cancer cells.^19^, ^26^, ^27^, ^28^, ^29^ TLR9 is also commonly expressed in many hematologic malignancies, including BCL.^30^, ^31^ Multiple clinical trials in NHL demonstrated safety but only limited efficacy of TLR9 agonists.^25^, ^30^ These difficulties can be, at least partly, ascribed to more recently described defects in TLR9 signaling in BCLs. Several studies have linked mutations in downstream TLR9 signaling (e.g., MYD88) or polymorphism in the *TLR9* promoter to the pathogenesis of aggressive NHL^10^, ^32^ or increased NHL incidence,^33^ respectively. Based on these observations, we developed dual-function CpG-STAT3 inhibitors to generate growth-inhibitory and immuno-mediated effects against DLBCL.

## Results

### Optimization of the CpG-STAT3dODN Strategy for Targeting BCL Cells

**Figure 1:**
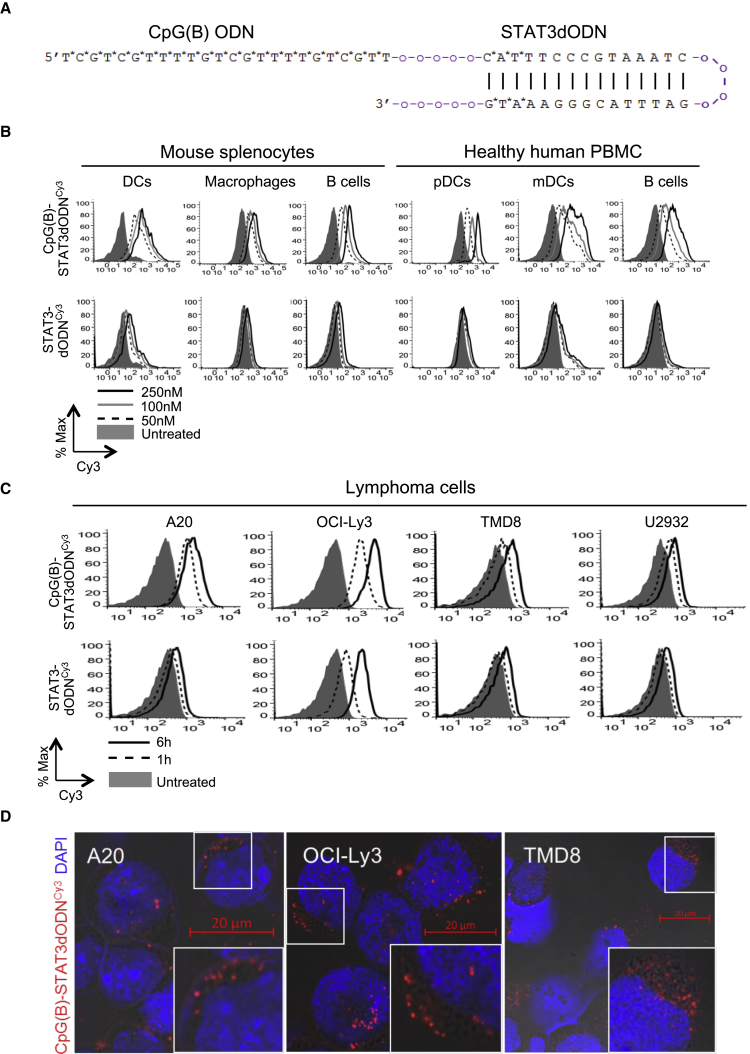
CpG(B)-STAT3dODN Design and Internalization into Specific Human and Mouse Target Cells (A) Structure and sequence of CpG(B)-STAT3dODN synthesized chemically as a conjugate of CpG7909 ODN with a double-stranded STAT3 decoy ODN; asterisks indicate phosphorothioation sites in the oligonucleotide backbone; “o” indicates single unit of the C3 carbon chain (CH2)_3_. (B) Dose-dependent internalization of CpG(B)-STAT3dODN^Cy3^ or the unconjugated STAT3dODN^Cy3^ by primary human peripheral blood mononuclear cells (PBMCs: CD1c^+^/pDCs; CD303^+^/mDCs; CD19^+^/B cells) or mouse splenocytes (CD11c^+^/DCs; F4/80^+^/macrophages; CD19^+^/B cells) after 4 hr incubation as measured using flow cytometry. (C) Uptake of 250 nM CpG(B)-STAT3dODN^Cy3^ or STAT3dODN^Cy3^ by human and mouse BCL cells after 1 or 6 hr. (D) Intracellular uptake of CpG(B)-STAT3dODN^Cy3^ by target human OCI-Ly3, TMD8, and mouse A20 lymphoma cells. Cells were incubated with 100 nM fluorescently labeled CpG7909-STAT3dODN^Cy3^ for 1 hr. The intracellular localization of the conjugate (red) and nuclei using DAPI (blue) was detected using phase contrast and confocal microscopy after 1 hr incubation. Shown are images from 1 of 3 independent experiments with similar results. Scale bars, 20 μm. % Max, percentage of maximum. *p < 0.05; **p < 0.01; ***p < 0.001.

We recently developed a strategy to deliver STAT3 decoy oligodeoxynucleotide inhibitor (STAT3dODN) into human myeloid cells after conjugation to the type-A TLR9 agonist, CpG ODN.^34^ While CpG(A)-STAT3dODN showed efficacy in targeting a variety of myeloid cell types, it showed moderate internalization by non-malignant B cells and BCLs. To improve the targeting of BCL, we modified the targeting sequence to the well-characterized, B-type CpG7909 that was previously evaluated in clinical trials in NHL patients (Figure 1A).^35^, ^36^ More extensive phosphorothioation (PS) of the new CpG(B)-STAT3dODN improved nuclease resistance of this conjugate, which showed an 82-hr half-life in the presence of human serum (Figure S1), compared to the 63-hr half-life previously reported for CpG(A)-STAT3dODN.^34^ Consistent with our previous study,^26^ primary human and mouse B cells and myeloid cells quickly and efficiently internalized fluorescently labeled CpG(B)-STAT3dODN^Cy3^, but not STAT3dODN^Cy3^ alone, even at a low 50-nM dose (Figure 1B). Furthermore, human activated B cell-like type (ABC)-DLBCL and mouse A20 lymphoma cells internalized CpG(B)-STAT3dODN^Cy3^ within 1–6 hr of incubation. The uptake of STAT3dODN alone was negligible, with the exception of the OCI-Ly3 cells, which internalized unconjugated decoy DNA, albeit less effectively (Figure 1C). Finally, we confirmed cytoplasmic localization of the CpG(B)-STAT3dODN after being internalized by target lymphoma cells, using phase-contrast and confocal microscopy (Figure 1D). Our results suggested that modified CpG(B)-STAT3dODN can effectively penetrate into immune and lymphoma cells, thereby enabling STAT3 targeting.

### CpG(B)-STAT3dODN Inhibits the Transcriptional Activity of STAT3

**Figure 2:**
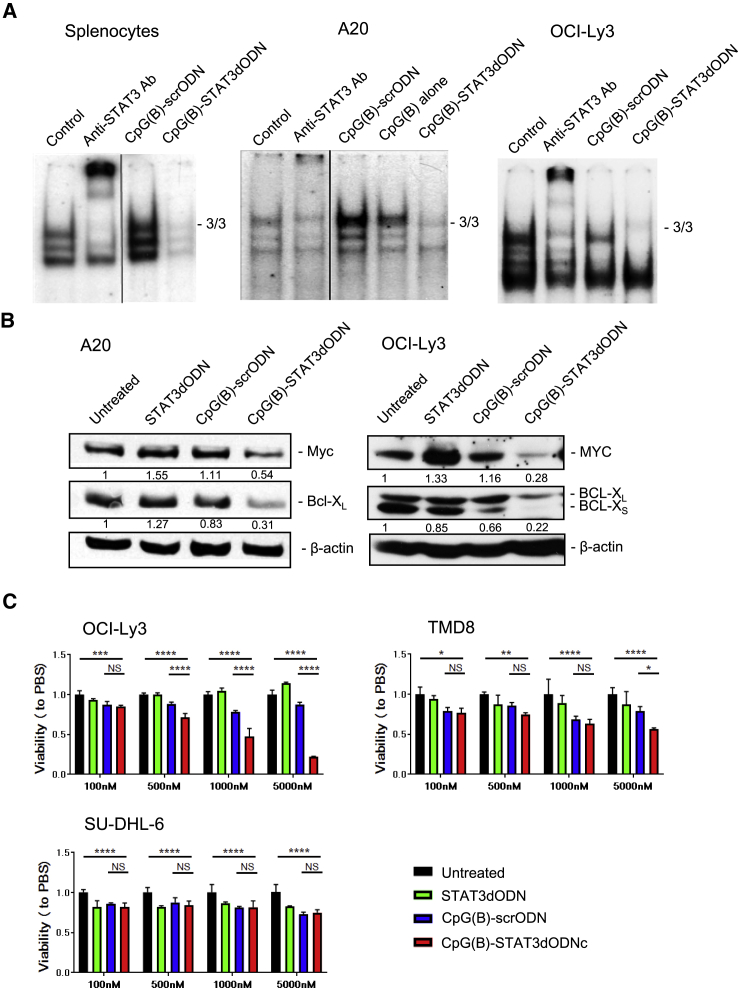
CpG(B)-STAT3dODN Reduces STAT3 DNA Binding and Downstream Target Gene Expression *In Vitro* (A) Mouse splenocytes, A20 cells, or human OCI-Ly3 cells were incubated for 48 hr with CpG(B)-STAT3dODN, CpG(B)-scrODN, or CpG(B) alone (500 nM). Next, cells were lysed, and the nuclease extracts were incubated with radioactive hSIE DNA probe specific to STAT3. STAT3-specific antibodies were used to confirm band identity (supershift): S3/S3 indicates position of STAT3 homodimers. (B) A20 and OCI-Ly3 cells were incubated for 72 hr in the presence of 500 nM CpG(B)-STAT3dODN, negative control CpG(B)-scrODN, or STAT3dODN alone. The expression level of c-MYC and BCL-X_L_ was assessed in cell lysates using western blotting. Protein band intensities were quantified densitometrically using ImageJ v.1.46 and normalized to levels of β-actin as indicated; shown are the results from one of three independent experiments with similar outcomes. (C) Dose-dependent cytotoxic effect of CpG-STAT3dODN on STAT3-dependent human ABC-DLBCL cells. 10^5^ human DLBCL cells (ABC-DLBCL: OCI-Ly3, TMD8; GC-DLBCL: SU-DHL6) per well were seeded in 96-well plate in IMDM/1% FBS medium. Cells were treated with CpG(B)-STAT3dODN or control oligonucleotides at concentrations as indicated for 3 days. The cell viability was detected using Vita-Orange Cell Viability Reagent (Biotool). STAT3-independent SU-DHL6 cells were used as a negative control. Shown are the results from one of two independent experiments performed in triplicates. Data indicate means ± SEM. *p < 0.05; **p < 0.01; ***p < 0.001, ****p ≤ 0.0001; NS, not significant.

Binding of the high-affinity decoy molecules to activated STAT3 dimers prevents downstream target gene transactivation.^20^ We utilized electrophoretic mobility shift assays (EMSAs) to assess the effect of CpG(B)-STAT3dODN on STAT3 binding to a STAT3-specific radiolabeled high affinity mutant of the c-Fos sis-inducible element (hSIE) probe. As shown in Figure 2A, CpG(B)-STAT3dODN abrogated almost completely the STAT3 activity in primary mouse splenocytes and also in mouse and human BCL cells, A20 and OCI-Ly3, respectively. In contrast, both control CpG(B)-scrODN and CpG(B) ODN alone increased STAT3 activity, especially in mouse target cells, which is a known effect of TLR9 signaling. The TLR9/nuclear factor κB (NF-κB) signaling induces the expression of IL-6 and/or IL-10, which activate STAT3 to restrain immunostimulation as a negative-feedback effect.^12^, ^37^, ^38^, ^39^ We further verified that the inhibition of STAT3 activity translates into reduced expression of downstream target proteins, such as BCL-X_L_ and c-MYC, in human and mouse lymphoma cells.^40^, ^41^ The protein levels of BCL-X_L_ and c-MYC were strongly downregulated by CpG(B)-STAT3dODN but not by the unconjugated STAT3dODN or control CpG(B)-scrODN in A20 and even more pronouncedly in OCI-Ly3 lymphoma (Figure 2B). Correspondingly, CpG(B)-STAT3dODN induced dose-dependent cytotoxicity in STAT3-dependent OCI-Ly3 and TMD8 ABC-DLBCL cells *in vitro*, while it had minimal effect on SU-DHL-6 germinal center (GC)-DLBCL or A20 cells (Figure 2C; data not shown).

Next, we tested the feasibility of using this strategy for targeting STAT3 *in vivo*. Mice with established, subcutaneously engrafted (s.c.-engrafted) A20 lymphoma were treated using repeated daily intratumoral (IT) injections of 1 mg/kg CpG(B)-STAT3dODN, control CpG(B)-scrODN, or PBS. Whole tumors were harvested 1 day after the third injection to assess STAT3 activation using EMSAs. While both CpG(B)-STAT3dODN and control CpG(B)-scrODN induced similar NF-κB activity (Figure S2), only the CpG(B)-STAT3dODN reduced STAT3 DNA-binding activity in A20 tumors (Figures S3A and S3B). The observed STAT3 inhibition correlated with a suppression of target *Bcl2l1* and *Myc* mRNA in A20 cells, as verified by real-time qPCR (Figure S3C).

### Local CpG(B)-STAT3dODN Treatment Inhibits Growth of Human ABC-DLBCL

**Figure 3:**
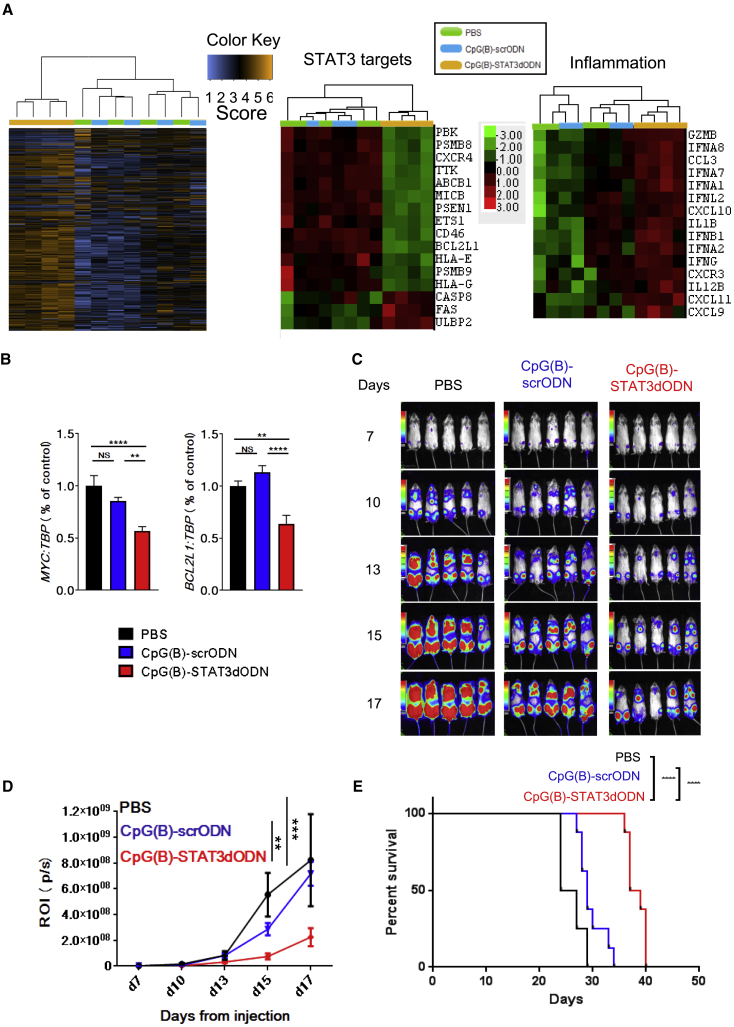
CpG(B)-STAT3dODN Inhibits Growth of Disseminated Human BCL (A and B) Local CpG(B)-STAT3dODN treatment reduces STAT3 signaling in OCI-Ly3 lymphoma xenotransplants. 1 × 10^7^ OCI-Ly3 cells were engrafted s.c. into NSG mice. Mice with established lymphoma were injected IT 3 times every day using 1 mg/kg CpG(B)-STAT3dODN, CpG(B)-scrODN, or PBS (n = 4). Total RNA isolated from harvested tumors was analyzed using NanoString analysis and the human-specific PanCancer Immune Profiling panel (A) and real-time qPCR (B). Data indicate means ± SEM. (C–E) Systemic administration of CpG-STAT3dODN inhibits growth of the disseminated OCI-Ly3^LUC^ lymphoma. 5 × 10^6^ OCI-Ly3^LUC^ cells were engrafted intravenously into NSG mice. Starting from day 7 after engraftment, mice were treated once daily for 10 days using injections (intravenous) of 10 mg/kg CpG(B)-STAT3dODN, CpG(B)-scrODN, or PBS alone. (C) Tumor progression was monitored using bioluminescent imaging (BLI); shown are representative images. (D) Tumor growth kinetics are indicated as assessed using quantification of the BLI signal during the experiment. ROI, regions of interest; p/s, photons per second. Data indicate means ± SEM (n = 5 per each group). (E) Systemic administration of CpG(B)-STAT3dODN extends the survival of OCI-Ly3-bearing mice. Shown are Kaplan-Meier survival curves derived from one of two independent experiments (n = 8). **p < 0.01; ***p < 0.001, ****p ≤ 0.0001; NS, not significant.

We used the human ABC-DLBCL model to verify the therapeutic effect of CpG(B)-STAT3dODN in immunodeficient NSG mice. As performed similarly in A20 lymphoma (Figure S3), three IT injections of CpG(B)-STAT3dODN (1 mg/kg), but not CpG(B)-scrODN or PBS, inhibited STAT3 DNA binding in human OCI-Ly3 lymphoma, as assessed using the EMSA (Figure S4). These results were further validated using the human gene-specific Nanostring assay on the total RNA isolated from whole OCI-Ly3 tumors treated using—similarly, as before—a short-term treatment with three IT injections of 1 mg/kg CpG(B)-STAT3dODN, CpG(B)-scrODN, or PBS. The heatmap analysis demonstrated clustering of the CpG(B)-STAT3dODN gene expression pattern versus that of both CpG(B)-scrODN and PBS (Figure 3A, left). We also confirmed downregulation of known STAT3 gene targets—e.g., *ABCB1*, *BCL2L1*, *CD46*, or *MICB*—specifically in the CpG(B)-STAT3dODN group, using Nanostring and qPCR analyses (Figures 3A, middle, and 3B). Changes in the expression of genes regulating proliferation (*TTK*) and cell death (*CASP8*, *FAS*, and *PBK*) suggested the onset of growth inhibition and apoptosis. In addition, we observed co-expression of immune mediators, such as interferons and their targets (*IFNA1-2*, *IFNA7-8*, *IFNB1*, and *IFNG*), as well as proinflammatory cytokines (*IL-1B* and *IL12B*) and chemokines and their receptors (*CXCL9-11* and *CXCR3*) by CpG(B)-STAT3dODN-treated lymphoma cells (Figure 3A, right). In immunodeficient NSG mice, these proinflammatory mediators of human origin could have only limited effect on innate, but not on adaptive, antitumor immunity. Nevertheless, these results suggested that the therapeutic effects of TLR9 triggering and STAT3 inhibition against BCL can be two-pronged: directly cytotoxic and immune mediated.

To assess the efficacy of systemic CpG(B)-STAT3dODN administration on OCI-Ly3 lymphoma, the immunodeficient NSG mice with established, disseminated-luciferase-expressing OCI-Ly3 lymphoma were treated using intravenous (i.v.) injections of 10 mg/kg CpG(B)-STAT3dODN, CpG(B)-scrODN, or only PBS for 10 days. The treatment with CpG(B)-STAT3dODN significantly delayed lymphoma progression in contrast to CpG(B)-scrODN control (Figures 3C and 3D). The growth inhibitory effect of CpG(B)-STAT3dODN on OCI-Ly3 lymphoma extended mouse survival versus both control groups. The effect of TLR9 stimulation alone (CpG(B)-scrODN) was relatively weak and failed to significantly affect mouse survival (Figure 3E).

### Systemic Administration of CpG-STAT3dODN Triggers T Cell-Mediated BCL Regression

**Figure 4:**
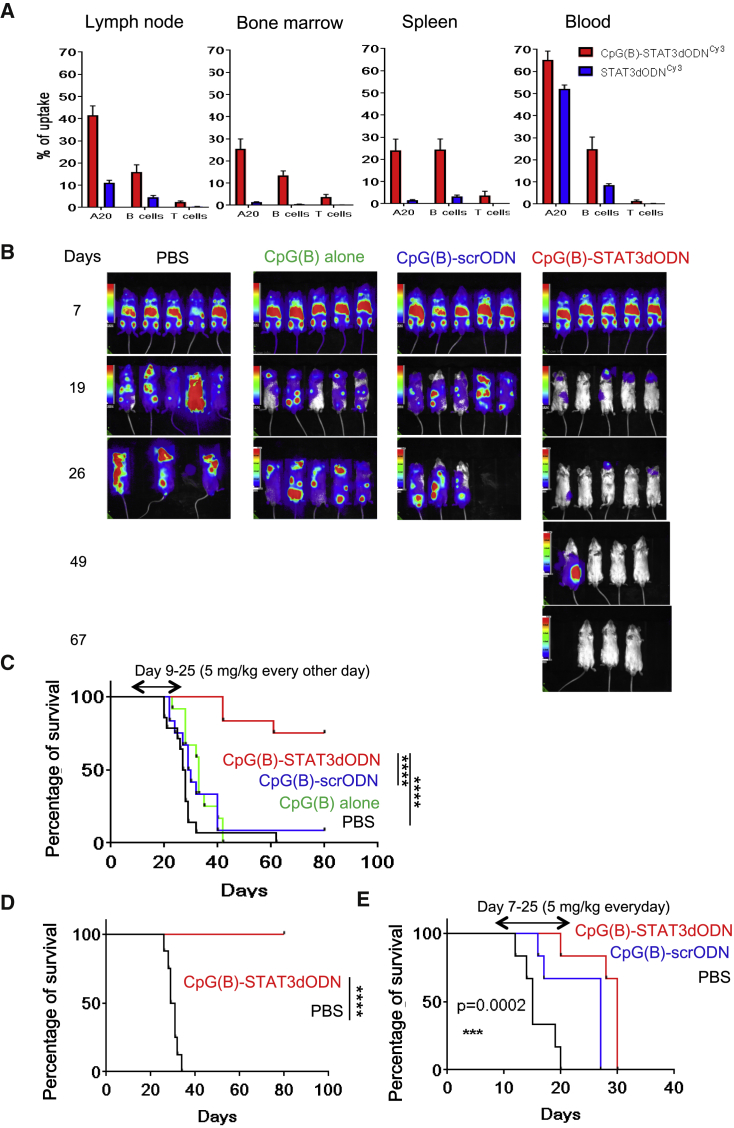
Systemic Administration of CpG(B)-STAT3dODN^Cy3^ Induces Regression and Protective Immunity against Syngeneic BCL in Immunocompetent Mice (A) Biodistribution of CpG(B)-STAT3dODN^Cy3^ is shown in comparison to the unconjugated STAT3dODN^Cy3^ after a single injection intravenously (5 mg/kg) in mice bearing A20^LUC^ lymphoma. Blood and indicated organs were collected 3 hr later to assess the percentage of Cy3-positive A20 cells (CD19^+^FSC-A^HI^), B cells (CD19^+^FSC-A^LO^), or CD3^+^ T cells using flow cytometry; results are from one of two independent experiments; data indicate means ± SEM (n = 4). (B) Mice with established A20^LUC^ lymphoma were treated every other day using 5 mg/kg CpG(B)-STAT3dODN, CpG(B)-scrODN, CpG(B) alone, or PBS. Lymphoma burden was monitored using BLI; representative images are from one of two independent experiments. (C) CpG(B)-STAT3dODN, but not CpG(B)-scrODN or CpG(B) alone (n = 12 per each group), results in long-term survival of the majority of mice. Survival curves for the indicated treatment groups combine results from two independent experiments. (D) CpG(B)-STAT3dODN treatment generates long-term protective immunity. Mice that survived initial tumor engraftment resisted re-challenge with A20^LUC^ lymphoma (n = 8), compared to the naive BALB/c mice (n = 8) used as positive controls. (E) Adaptive immunity is required for the durable therapeutic effect of CpG(B)-STAT3dODN against syngeneic BCL. The immunodeficient NSG mice with established, disseminated A20 lymphoma were administered intravenous treatment every day, using 5 mg/kg CpG(B)-STAT3dODN, CpG(B)-scrODN, or PBS. Shown are survival curves for various treatment groups (n = 6 per group). ***p < 0.001, ****p ≤ 0.0001.

**Figure 5:**
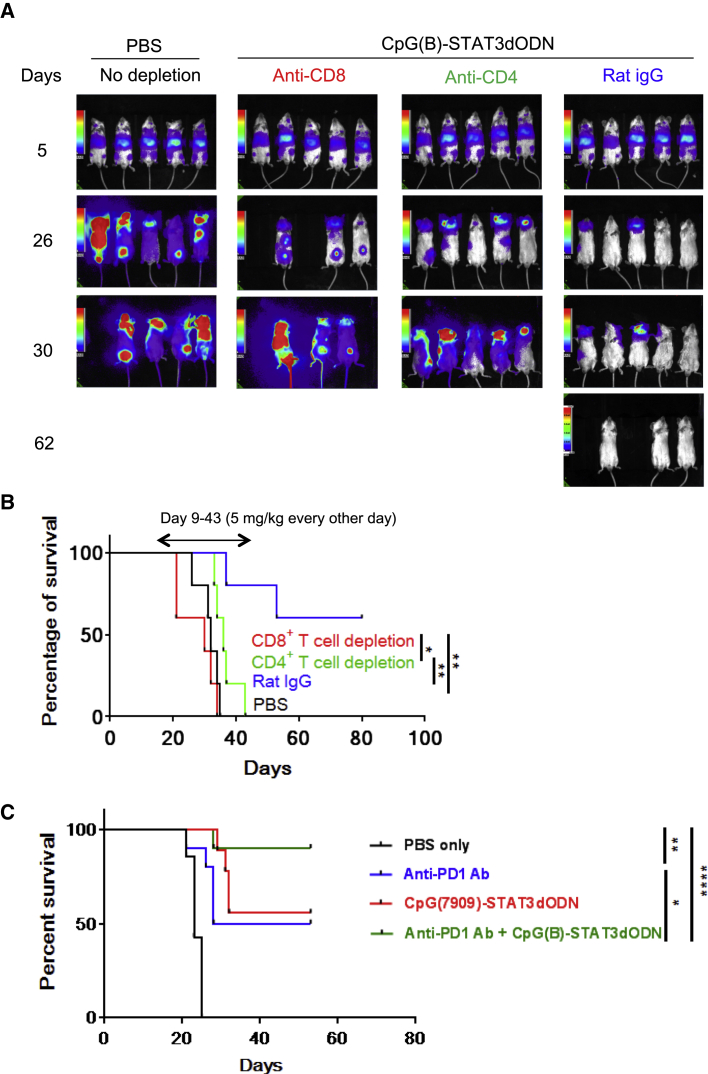
Therapeutic Effect of CpG(B)-STAT3dODN against BCL Relies on T Cell Activity and Can Be Augmented by Combination with PD1 Blockade (A and B) CD8^+^ and CD4^+^ T cell populations were depleted in mice engrafted with A20^LUC^ lymphoma using 200 μg i.p. anti-CD8 (2.43), anti-CD4 (GK1.5), or control rat IgG (Sigma-Aldrich) at day 6, day 8, and then once a week for the rest of the experiment. Starting from day 9, mice were administered an intravenous injection using 5 mg/kg CpG(B)-STAT3dODN every other day. (A) Lymphoma burden was monitored using BLI; shown are the representative images. (B) Combined results showing Kaplan-Meier survival curves for the indicated treatment groups (n = 5 per group). IgG, immunoglobulin G. (C) Mice with established systemic A20^LUC^ lymphoma were treated using CpG(B)-STAT3dODN (5 mg/kg, every other day from day 9) and anti-PD1 antibody (eBioscience; 200 μg i.p.) injected alone or in combination. Kaplan-Meier survival curves are shown for the indicated treatment groups (n = 10 per group, except for PBS: n = 7). *p < 0.05, **p < 0.01, ****p ≤ 0.0001.

High nuclease resistance enables the systemic delivery of CpG(B)-STAT3dODN to disseminated BCL cells and non-malignant lymphocytes. We used fluorescently labeled CpG(B)-STAT3dODN^Cy3^ to assess oligonucleotide biodistribution. BALB/c mice with established, disseminated A20 lymphomas received a single intravenous injection of CpG(B)-STAT3dODN^Cy3^. Peripheral blood, lymph nodes, spleen, and bone marrow were collected after 3 hr to determine CpG(B)-STAT3dODN biodistribution using flow cytometry. The internalization by A20 cells in various tissues ranged from 25% to 65% (Figure 4A). The uptake by non-malignant B cells was lower at 15%–25% and negligible for T cells in all locations, consistent with the *in vitro* uptake of CpG(B)-STAT3dODN (Figure 1B). Although intravenous injections resulted only in partial penetration of the BCL compartment, based on our previous study,^34^ such an internalization rate can be sufficient for the induction of systemic antitumor immunity using CpG-STAT3 inhibitors. Thus, we next assessed the antitumor efficacy of CpG(B)-STAT3dODN administered systemically. BALB/c mice with disseminated A20^LUC^ were treated using, every other day, intravenous injections of 5 mg/kg CpG(B)-STAT3dODN, CpG(B)-scrODN, or CpG(B) alone or were treated with vehicle only (PBS). Mice were treated until day 25 and then monitored without further treatment using bioluminescent imaging and body condition scoring. Within 10 days, CpG(B)-STAT3dODN treatments arrested A20^LUC^ lymphoma progression (Figure 4B) and then led to complete lymphoma regression in 75% of mice (Figure 4C). In contrast, control CpG(B)-scrODN and clinically relevant CpG(B)/CpG7909 or CpG7909, co-injected in equimolar amounts with the unconjugated STAT3dODN, had minimal and only transient inhibitory effects on lymphoma progression (Figures 4B and S5, respectively). Importantly, all CpG(B)-STAT3dODN-treated mice (0/8) that survived the initial A20 challenge were resistant to the rechallenge using the same lymphoma cells, while all naive mice (8/8) showed A20 engraftment (Figure 4D). CpG(B)-STAT3dODN efficacy was also superior when compared to that of atovaquone, a recently described STAT3 inhibitor with activity against human multiple myeloma and acute myeloid leukemia (AML) (Figure S6).^42^ The extended therapeutic effect of the single 2-week treatment cycle using CpG(B)-STAT3dODN and its long-term protective antitumor effect were indicative of immune-mediated antitumor responses. To assess the contribution of directly cytotoxic and immune-dependent antitumor effects, we engrafted A20^LUC^ lymphoma into the immunodeficient NSG mice. Following the engraftment as verified by bioluminescent imaging (BLI), mice were treated for 2 weeks of daily injections of CpG(B)-STAT3dODN, CpG(B)-scrODN, or PBS alone (Figure 4E). The CpG(B)-STAT3dODN failed to eliminate tumors in NSG mice and only weakly improved their survival, similar to CpG(B)-scrODN. These results likely indicate the limited effect of TLR9 stimulation on innate immune cells, such as granulocytes/neutrophils still active in NSG mice,^19^ while underscoring the crucial role of adaptive immune responses in generating durable lymphoma regression. We further verified the contribution of CD8^+^ and CD4^+^ T cells to these therapeutic effects, using antibody-mediated depletion. The CD8^+^ T cell neutralization had the strongest negative impact on the CpG(B)-STAT3dODN effect against A20 lymphoma (Figure 5A). In the absence of CD8^+^ T cells, lymphoma progression was accelerated compared to that of other treatment groups, and mouse survival did not differ from that of the control PBS-treated mice (Figure 5B). The CD4-specific cell depletion completely alleviated the late onset of CpG(B)-STAT3dODN-induced antitumor immunity at 2 weeks after treatment initiation (Figure 5A). However, it did not prevent the early delay in A20 lymphoma progression, which resulted in minimally extended animal survival (Figure 5B). In contrast, over half of CpG(B)-STAT3dODN-treated reference mice (CpG(B)-STAT3dODN/IgG) survived lymphoma challenge and remained tumor free for >80 days (Figure 5B). After confirming the essential role of T cell immunity in mediating CpG(B)-STAT3dODN effects *in vivo*, we assessed whether therapeutic efficacy of this strategy can be enhanced by blocking PD1/PD-L1 immune checkpoint regulation, which often results in the reduced activity of exhausted T cells. As shown in Figure 5C, systemic administration of CpG(B)-STAT3dODN or anti-PD1 antibodies as single agents showed comparable effects against A20 lymphoma, with ∼50% mouse survival. Importantly, the combination of both strategies strongly improved therapeutic outcome, with 90% survival of mice (Figure 5C). These results suggest that the presence and activity of T cells are critical for the overall antitumor activity of CpG(B)-STAT3dODN against BCL.

### TLR9 Stimulation and STAT3 Inhibition Prompts Immunogenicity of Lymphoma Cells

**Figure 6:**
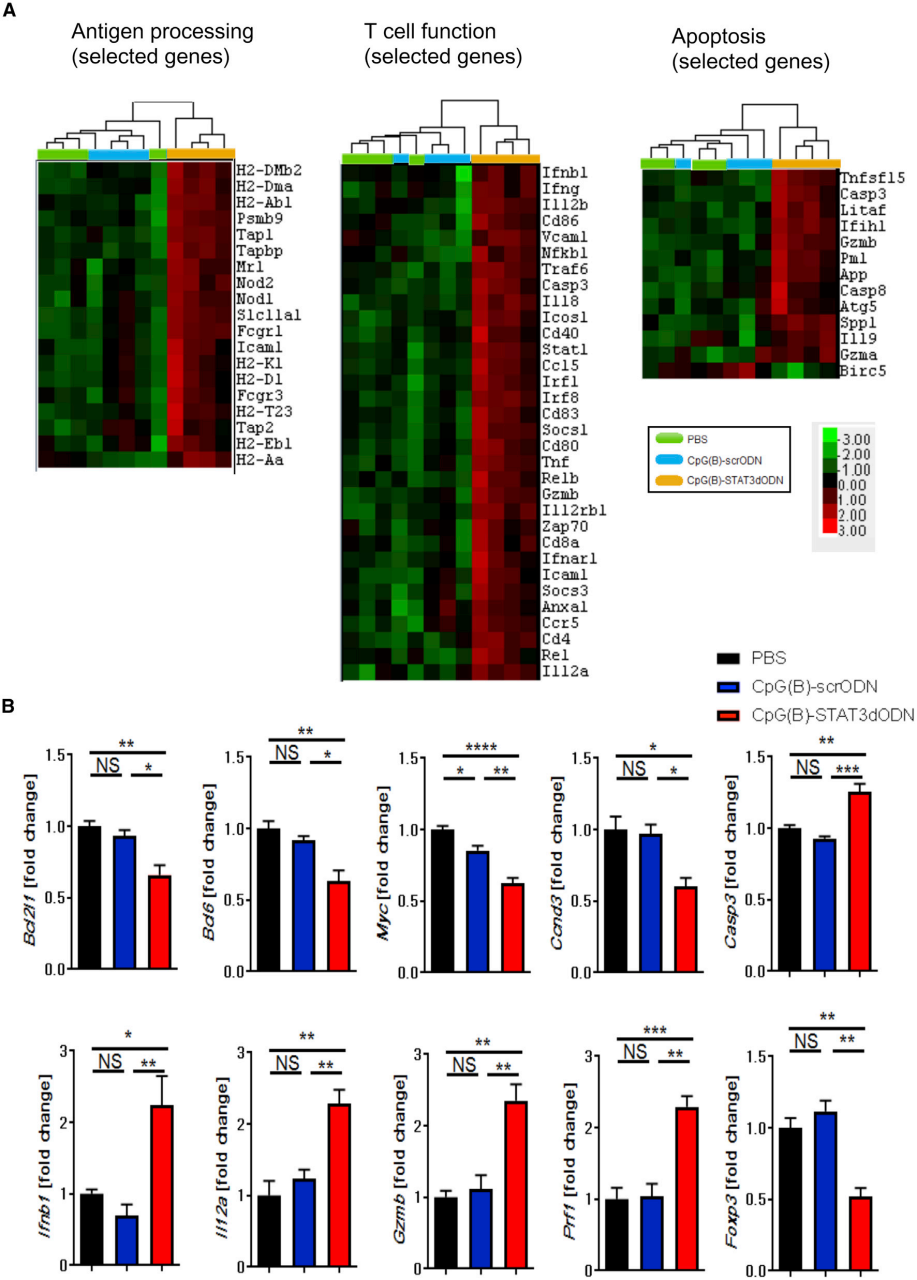
Gene Expression Profiling of BCL following TLR9 Stimulation with or without Concurrent STAT3 Inhibition Mice with s.c.-established A20 lymphoma were treated starting on day 14 after tumor challenge using 1 mg/kg CpG(B)-STAT3dODN, control CpG(B)-scrODN, or PBS three times every other day (n = 4 per each group). Before tumor volumes differed, tumors were harvested to isolate total RNA for NanoString analysis using the mouse-specific PanCancer Immune Profiling panel. (A) Heatmap analyses for selected functional groups of genes related to antigen processing (left), T cell function (middle), and apoptosis (right). (B) Graph bars representing expression of specific targets from apoptosis- and T cell function-related groups; data indicate means ± SEM (n = 4). *p < 0.05, **p < 0.01, ***p < 0.001, ****p ≤ 0.0001; NS, not significant.

To gain insights into potential molecular mechanisms underlying the antitumor effect of CpG(B)-STAT3dODN *in vivo*, we performed Nanostring gene expression analysis on A20 BCLs. Mice with s.c.-engrafted A20 tumors were treated using three IT injections of 1 mg/kg CpG(B)-STAT3dODN, CpG(B)-scrODN, or PBS every other day. We then examined gene expression profiles in whole A20 tumors from different treatment groups. Among the 770 immune-regulation-related genes, 465 genes were significantly altered (p < 0.05), with 203 genes significantly upregulated more than 2-fold. The initial analysis revealed a clearly distinct transcription signature of CpG(B)-STAT3dODN-treated tumors compared to that of both vehicle- and CpG(B)-scrODN-treated groups, which clustered together, indicating a limited outcome of TLR9 triggering alone (Figure S7A). In contrast, CpG(B)-STAT3dODN resulted in the significant upregulation of signaling pathways involved in immunoregulation (Figure S7B). Further analysis indicated that TLR9 triggering and STAT3 inhibition elevated genes involved in antigen processing/presentation, such as major histocompatibility complex (MHC) class I-related genes (e.g., *H2-D1*, *H2-K1*, *Psmb9*, *Tap1*, *Tap2*, and *Tapbp*) and class II-related genes (e.g., *H2-DM1* and *H2-DMb2*), and modulated the essential regulators of T cell activation (e.g., *Cd40/80/83/86*, *Foxp3*, *Icosl*, and *Il12a/b*) or cytotoxic functions (*Gzma/b*, *Ifnb1*, *Ifng*, and *Prf1*) (Figures 6A and 6B). Expression of the several STAT3-controlled mediators of lymphoma cell proliferation and survival, including *Bcl2l1*, *Bcl6*, *Casp3*, *Ccnd3*, and *Myc*, was reduced (Figure 6B). These gene expression profiles were reflected by significant increase of both cytotoxic cell and Th1 cell scores, with a decrease in cell cycle score, specifically in CpG(B)-STAT3dODN-treated lymphomas, but not in controls (Figure S7C).

**Figure 7:**
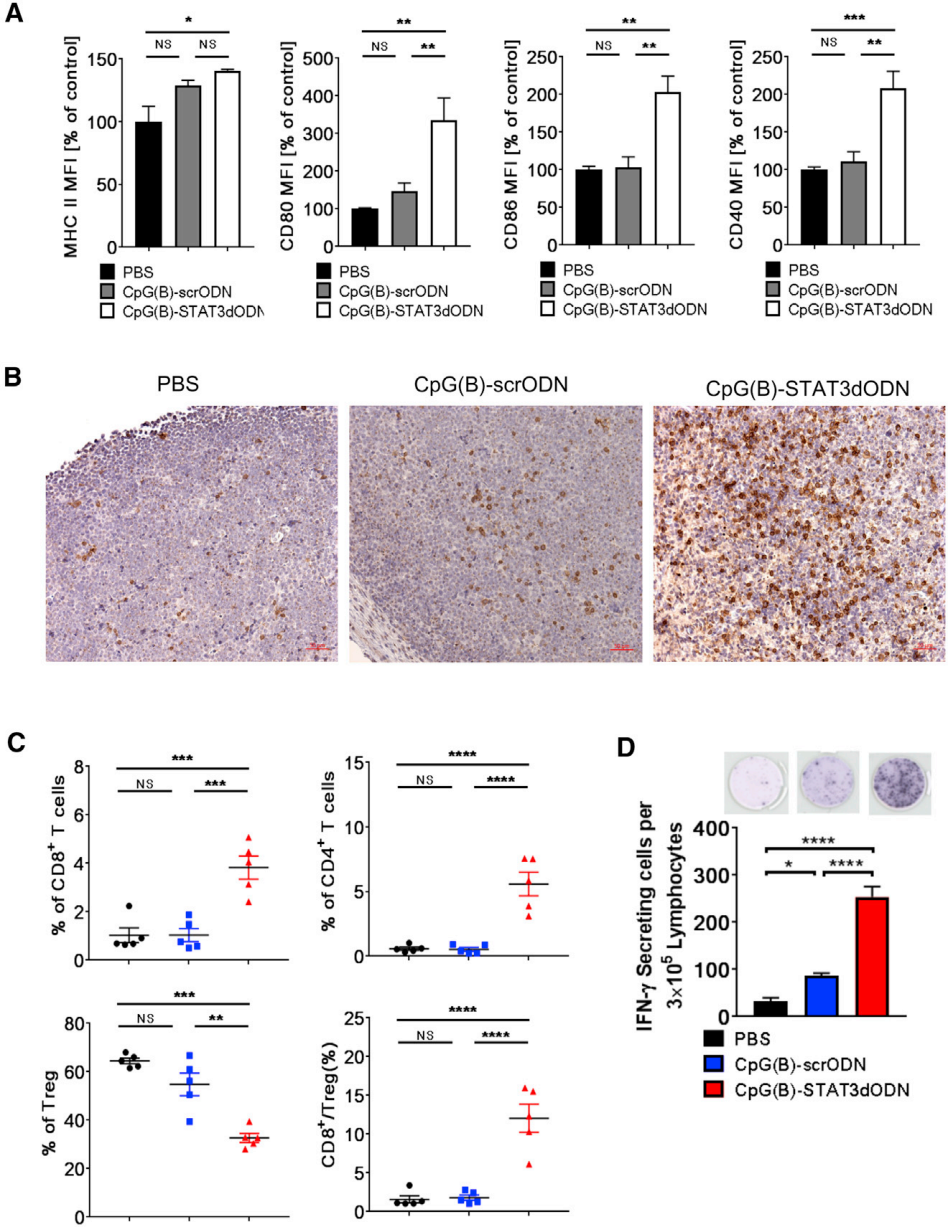
CpG(B)-STAT3dODN Treatment Results in B Cell-Lymphoma-Specific T Cell Immune Responses with Tumor Infiltration by CD8^+^ T Cells Mice with s.c.-established A20 lymphoma were injected IT using 1 mg/kg CpG(B)-STAT3dODN, CpG-scrODN, or PBS 4 times every other day. Tumors were harvested and dispersed into single-cell suspensions for the phenotypic immune marker analysis using flow cytometry. (A) CpG(B)-STAT3dODN upregulates surface expression of MHC class-II, costimulatory CD40, CD80, and CD86 molecules. Data indicate means ± SEM (n = 5) from one of two independent experiments with similar outcomes. (B) CD8^+^ T cell infiltration in A20 lymphoma tumors visualized using immunohistochemical staining on FFPE tumor sections. Scale bars, 50 μm. (C) The infiltration by various T cell populations in A20 tumors were assessed using flow cytometry. Shown are percentages of total CD3^+^CD8^+^, CD3^+^CD4^+^ T cells, and CD3^+^CD4^+^Foxp3^+^ regulatory T cells (Tregs), as well as the ratio of CD3^+^CD8^+^ T cells to Tregs. (D) TLR9 stimulation combined with STAT3 inhibition generates A20 B cell-lymphoma-specific immune responses. Recall response to irradiated (100 Gy) A20 cells was assessed using single-cell suspensions prepared from tumor-draining lymph nodes from *in vivo*-treated mice as described above. Numbers of IFNγ-secreting cells were assessed using an ELISPOT assay. Upper panel: representative images; bottom panel: bar graphs representing results from one of two independent experiments. Data indicate means ± SEM (n = 5). *p < 0.05, **p < 0.01, ***p < 0.001, ****p ≤ 0.0001; FFPE, formalin-fixed, paraffin embedded; MFI, mean fluorescence intensity; NS, not significant.

These results prompted us to investigate whether CpG(B)-STAT3dODN can directly enhance the immunogenicity of BCL. Similarly as before, mice with s.c.-established A20 BCL were treated every other day using 1 mg/kg CpG(B)-STAT3dODN, CpG(B)-scrODN, or only PBS. A day after the fourth injection, tumors were harvested to analyze the phenotype of lymphoma cells and T cell infiltration. We first assessed phenotypic changes in surface markers related to the potential immunogenicity of A20 cells. CpG(B)-STAT3dODN, but not the control CpG(B)-scrODN, moderately enhanced the already high basal MHC class-II levels on BCL cells, but it led to a 2- to 3-fold increase in the expression of costimulatory molecules: CD80, CD86, and CD40 (Figure 7A). These changes in the phenotype of BCL cells were reflected by strongly enhanced CD8^+^ T cell infiltration of A20 lymphoma verified by immunohistochemical staining (Figure 7B). More specific flow-cytometric analysis confirmed 4- to 5-fold increases in the percentage of tumor-infiltrating CD8^+^ and CD4^+^ T cells, with concomitant reduction in the percentage of CD4^+^/FoxP3^+^ regulatory T cells (Tregs). These changes produced an average 12-fold increase in the ratio of CD8^+^ to Tregs, which can be indicative of an effective, adaptive antitumor immune response (Figure 7C). Finally, we verified whether CpG(B)-STAT3dODN-induced immune responses are specific for A20 BCL antigens. The irradiated A20 cells were used as a source of tumor antigens to trigger recall antitumor responses in T cells isolated from the lymph nodes of A20-lymphoma-bearing mice following treatment using CpG(B)-STAT3dODN, CpG(B)-scrODN, or PBS. The ELISPOT assay detected highly elevated numbers of IFNγ-secreting lymphocytes from CpG(B)-STAT3dODN-treated mice, compared to a significant but weak response to CpG(B)-scrODN treatment, versus PBS control mice (Figure 7D). Despite the potent antitumor efficacy of CpG(B)-STAT3dODN *in vivo*, we did not observe toxicities or autoimmune manifestations in immunocompetent mice over the 2-week treatment. Toxicology studies and histopathological analyses, in acute and chronic settings, failed to detect dose-limiting toxicities or organ gross abnormalities in CpG(B)-STAT3dODN-treated mice for up to 60 mg/kg/week (Figure S8). These results confirm that CpG(B)-STAT3dODN can be safe and well tolerated during repeated systemic administration. Finally, the immunostimulatory effect of CpG(B)-STAT3dODN on human PBMCs at low or high cell density was moderate, comparable with the effect of CpG(B) alone, and did not result in a “cytokine storm” (Figure S9).^43^ Overall, our results support the feasibility and safety of using the combination of TLR9 stimulation with STAT3 inhibition for treatment of BCLs. The CpG(B)-STAT3dODN strategy has the potential to augment therapeutic effects by combining direct cytotoxicity with strong CD8^+^ and CD4^+^ T cell-mediated antitumor immunity.

## Discussion

In this study, we demonstrate that a conjugate of STAT3 decoy inhibitor with TLR9 ligand, CpG(B)-STAT3dODN, provides an *in vivo* strategy for cell-selective delivery to BCL cells. Without the need for pharmacological formulation, the unformulated CpG(B)-STAT3dODN, injected locally or systemically, was effective in blocking STAT3 in lymphoma cells. Repeated treatment with CpG(B)-STAT3dODN had a therapeutic effect against established human DLBCL xenotransplants, inhibiting their growth by a direct cytotoxic/cytostatic effect. While the therapeutic efficacy of CpG(B)-STAT3dODN in immunocompetent mice relied mainly on the potent T cell-mediated antitumor immunity, it was, at least partly, supported by the direct antitumor effect. The exact contribution of indirect versus direct antitumor effects will be explored in our further studies in TLR9-deficient lymphoma models. Importantly, the dual-function antitumor effects required the combination of TLR9 agonist (CpG ODN) with STAT3 inhibitor in one molecule and were not observed for CpG7909 ODN or STAT3 inhibitor when injected alone or even when co-injected. The STAT3 inhibition, through the reversible decoy effect, combined with concurrent TLR9 activation seemed sufficient for the induction of the lymphoma differentiation to antigen-presenting phenotype, thereby ensuring therapeutic antitumor efficacy.

Previous studies by other groups demonstrated that TLR9 agonists—such as CpG7909, used in our study—inhibit the growth of cultured BCL cells and stimulate their immunogenicity.^25^, ^30^, ^44^ Preclinical studies in mice suggested that the increased immunogenicity of BCL cells can contribute to systemic antitumor immune responses generated by local administration of CpG ODNs, creating an effect of *in situ* vaccination.^45^ However, these effects required 5- to 10-fold higher concentrations of TLR9 agonists and direct IT injections to achieve antitumor effects.^44^, ^46^, ^47^ To overcome these limitations, TLR9 agonists in preclinical studies, and later in clinical trials, were combined with either chemotherapy, radiotherapy, or targeted antibody-based strategies (e.g., rituximab).^25^, ^47^, ^48^ Our previous studies and current observations suggest that blocking STAT3 removes restraints on the potency of TLR9-driven immunostimulation.^26^, ^28^, ^38^, ^49^ This is in agreement with an original report in mouse lymphoma cells that suggested the role of STAT3 in restricting BCL cell immunogenicity.^50^ Importantly, while mutations may prevent direct effects of STAT3 inhibition on BCL cells, the increased activity of non-malignant antigen-presenting cells can be sufficient for the induction of antitumor immune responses.^51^ Several small-molecule JAK inhibitors and STAT3 antisense oligonucleotides are currently in clinical trials for hematologic malignancies, including drug-resistant or relapsed BCL cells.^22^, ^52^ Whether their efficacy partly relies on immune-mediated effects is not yet clear. The non-selective, systemic Jak/STAT inhibition potentially might impede IFN-mediated antitumor T cell immunity and/or generation of memory T cells.^11^ In contrast, emerging clinical immunotherapies activate T cells by directly blocking immune checkpoint molecules, such as PD1, responsible for T cell exhaustion. Given that targeting PD1 has already shown promise in initial phase I/II clinical trials in patients with advanced-stage NHL, including DLBCL, these strategies are likely to benefit from the combination with immunogenic CpG(B)-STAT3dODN.^52^ Therefore, TLR9-targeted delivery of STAT3 inhibitors, as single agents or in combination with T cell-based approaches, offers a novel strategy for safer and more effective immunotherapy of BCL and, potentially, other hematologic malignancies.

## Materials and Methods

### Cells

PBMCs from anonymous healthy donors were collected in accordance with the Declaration of Helsinki under the institutional review board (IRB) protocol 13378 (City of Hope; COH).^19^ Cell viability was >90%, as confirmed using flow cytometry. Mouse A20 and human OCI-Ly3 cells were from ATCC or DSMZ, respectively, and TMD8 and U2932 ABC-DLBCL cells were kindly provided by Dr. G. Inghirami (Weill Cornell Medicine, New York, NY, USA). Cells were cultured in RPMI 1640/10% fetal bovine serum (FBS) media. To generate A20^LUC^ and OCI-Ly3^LUC^ cells, parental cells were transduced with *luciferase*/*mCherry* using a lentiviral vector.^53^

### Mice

All animal experiments were followed established institutional guidance and approved protocols from the institutional animal care and use committee (COH). BALB/c mice were purchased from the National Cancer Institute (Frederick, MD, USA). *NOD/SCID/IL-2RγKO* (NSG) mice, originally from the Jackson Laboratory, were maintained at the COH. Mice were injected intravenously or s.c. with 5 × 10^6^ OCI-Ly3^LUC^ or A20^LUC^ in PBS, and lymphoma engraftment/progression was monitored using BLI on the AmiX (Spectral Instruments).

### Oligonucleotides

All oligonucleotides were synthesized in the DNA/RNA Synthesis Core (COH) by linking CpG7909 to STAT3dODN in a manner similar to one previously described.^26^ The resulting conjugates are shown below (x = single C3 unit; asterisks indicate phosphorothioation sites):

#### CpG(B)-STAT3dODN

5′-T*C*G*T*C*G*T*T*T*T*G*T*C*G*T*T*T*T*G*T*C*G*T*T-xxxxx-C*A*T*TTCCCGTAAATC-xxxx-GATTTACGGGAA*A*T*G-xxxxx-3′

#### CpG(B)-scrambledODN

5′-T*C*G*T*C*G*T*T*T*T*G*T*C*G*T*T*T*T*G*T*C*G*T*T -xxxxx-A*C*T*CTTGCCAATTAC-xxxx-GTAATTGGCAAG*A*G*T-xxxxx-3′

#### STAT3dODN

5′-C*A*T*TTCCCGTAAATC-xxxx-GATTTACGGGAA*A*T*G-xxxxx-3′

#### CpG(B/7909)

5′-T*C*G*T*C*G*T*T*T*T*G*T*C*G*T*T*T*T*G*T*C*G*T*T-3′

For internalization/biodistribution studies, oligonucleotides were 3′-labeled using Cy3 fluorochrome.

### EMSAs and Protein Detection

EMSAs to detect STAT3 DNA-binding activity were performed as described previously.^26^ Briefly, 10 μg nuclear extracts were incubated with hSIE ^32^P-labeled oligonucleotide probes specific to STAT3 (hSIE)^54^ for supershift control using anti-STAT3 antibody (Santa Cruz Biotechnology). Western blot detection to detect STAT3, pSTAT3, BCL-X_L_, c-Myc, and β-actin expression was performed as described earlier.^19^

### Cell Viability Assay

10^4^ DLBCL lymphoma cell lines were seeded in 100 μL 1% FBS Iscove’s Modified Dulbecco’s Medium (IMDM) in a 96-well plate and incubated in the presence of designated reagent for 3 days. 10 μL Vita-Orange Cell Viability Reagent (Biotool, B34304) was added into each well, and the mixture was incubated at 37°C with 5% CO_2_ for 4 hr. Optical density (OD) at 450 nm was detected using Citation3, with OD at 690 nm as reference.

### Immune Assays

For extra-/intracellular staining, fluorochrome-labeled antibodies and previously described staining protocols were used (eBioscience).^17^, ^29^ Data collected on the BD-Accuri C6 and BD-Fortessa (BD Biosciences) were analyzed using FlowJo software (TreeStar). The ELISPOT assay was performed following manufacturer’s protocol (CellSciences), as described previously.^28^ Immunohistochemistry using anti-CD8 antibody (eBioscience/4SM15) was performed as described previously^19^ and was analyzed on the Observer II microscope (Zeiss). For confocal microscopy, cells were fixed in 2% paraformaldehyde (Electron Microscopy Sciences); then, nuclei were stained using DAPI (Sigma-Aldrich), and slides were analyzed using an inverted confocal microscope (LSM880-Airyscan; Zeiss) and the LSM Image Browser (Zeiss, v.4.2.0.121).^27^

### NanoString Analysis

Total RNA was extracted from whole OCI-Ly3 or A20 tumors using the mirVana miRNA Isolation Kit (Ambion). RNA quality was verified using the Bioanalyzer-2100 (Agilent). Gene expression was analyzed using the PanCancer Immune Profiling panel for human or mouse, XT-CSO-HIP1-12 or XT-CSO-MIP1-12, respectively, on the nCounter system (NanoStringTechnologies) following the manufacturer’s recommendations. Results were analyzed using nSolver 3.0 software (NanoStringTechnologies) and automated normalization of raw data.

### Statistical Analysis

An unpaired t test was used to calculate the two-tailed p value to estimate statistical significance of differences between two experimental groups. A one-way ANOVA plus Bonferroni posttest were applied to assess the statistical significance of differences between multiple treatment groups. The relationship between two groups was assessed using correlation and linear regression. The p and r^2^ values are indicated in the figures with asterisks: *p < 0.05; **p < 0.01; ***p < 0.001. Data were analyzed using Prism v.6.03 software (GraphPad).

## Author Contributions

Study design: M.K. and X.Z.; Conducting experiments: X.Z., Z.Z., D.M., Y.-L.S., H.W., and T.A.; Data analysis/interpretation: M.K., S.F., L.K., X.Z., Z.Z., H.H.Y., and R.K.P.; Providing reagents: Y.L., Z.D., P.S., and L.K.; Writing manuscript: Z.Z., X.Z., and M.K.

## Supplemental Information

Document S1. Supplemental Materials and Methods and Figures S1–S9

Document S2. Article plus Supplemental Information
