# Supplementary material for: B Cell Lymphoma Immunotherapy Using TLR9-Targeted Oligonucleotide STAT3 Inhibitors
Source: Mol Ther. 2018 Jan 17;26(3):695–707. doi: 10.1016/j.ymthe.2018.01.007 (PMC5910676; doi:10.1016/j.ymthe.2018.01.007)
Supplement: Document S1. Supplemental Materials and Methods and Figures S1–S9 [file mmc1.pdf]

YMTHE, Volume *26*

Supplemental Information

B Cell Lymphoma Immunotherapy Using TLR9-Targeted Oligonucleotide STAT3 Inhibitors

Xingli Zhao, Zhuoran Zhang, Dayson Moreira, Yu-Lin Su, Haejung Won, Tomasz Adamus, Zhenyuan Dong, Yong Liang, Hongwei H. Yin, Piotr Swiderski, Raju K. Pillai, Larry Kwak, Stephen Forman, and Marcin Kortylewski

Supplementary Materials and Methods

Western blot for phospho-iκBα

10^6 of mouse A20 cells or human OCI-Ly3 lymphoma cells were engrafted SC into BALB/C or NSG mice, respectively. After tumors were established, mice were treated using 5 mg/kg CpG(B)-STAT3dODN, control CpG(B)-scrODN or PBS every day for 3 days. Tumors were harvested and protein was extracted for Western blot detection of phospho-iκBα (Cell signaling) levels compared to β-actin (Santa Cruz) used a s a loading control.

Animal studies

BALB/c mice with established disseminated $A20^{LUC}$ lymphoma were treated using 5 mg/kg of CpG(B)-STAT3dODN injected every other day, 200mg/kg of atovaquone (Mepron™) injected daily or PBS. Lymphoma burden was monitored using BLI for up to 100 days for surviving mice.

Luminex assays

Cytokine secretion from cultured healthy donors' PBMC was analyzed using Human Magnetic Luminex Screening Assay (R&D) on Flexmap 3D system (Luminex).

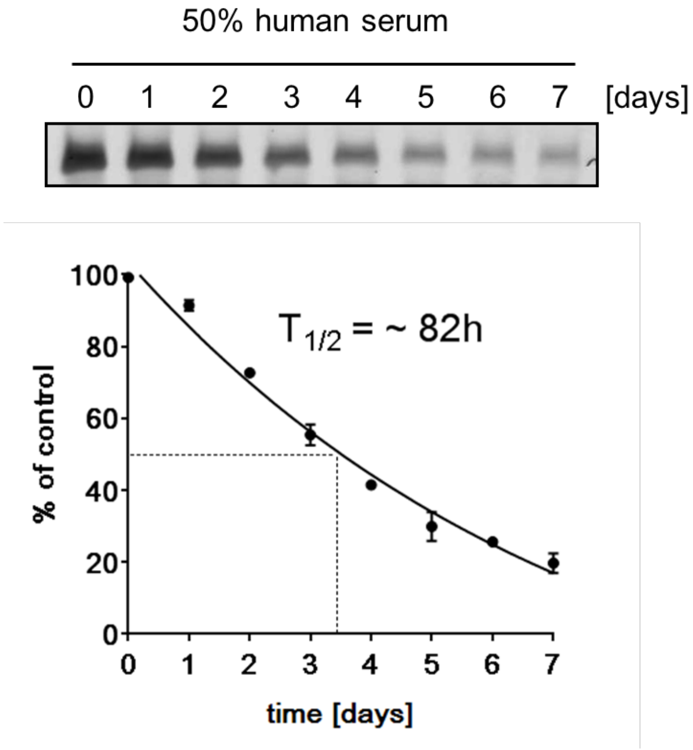

Supplemental Fig. S1. Stability of chemically modified CpG-STAT3dODN in human serum. CpG(B)-STAT3dODN were incubated in 50% human serum at 37°C for the indicated times. The samples were then resolved on the 7.5 M Urea/20 % PAGE gel and stained using ethidium bromide; the representative gel image for CpG(B)-STAT3dODN is shown in the upper panel. Bottom panel shows the quantification of band intensities for CpG(B)-STAT3dODN; shown are means ± SEM (*n* = 3). The estimated half-life ($T_{1/2}$) of CpG(B)-STAT3dODN was indicated.

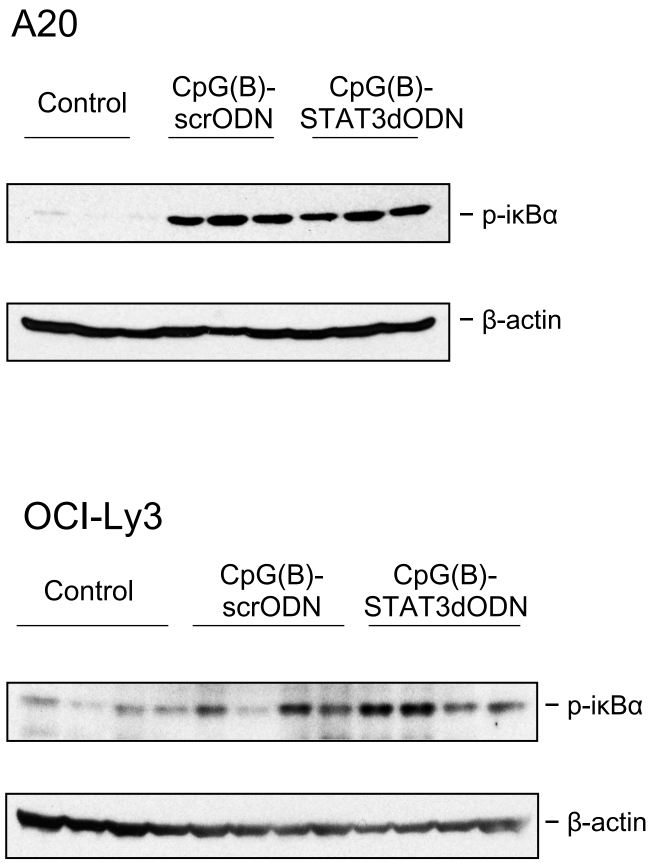

Supplemental Fig. S2. ***In vivo*** **CpG(B)-STAT3dODN treatment results in activation of NF-κB signaling.** (A) 10^6 of mouse A20 cells or human OCI-Ly3 lymphoma cells were engrafted SC into BALB/C or NSG mice, respectively. After tumors were established, mice were treated using 5 mg/kg CpG(B)-STAT3dODN, control CpG(B)-scrODN or PBS every day for 3 days. Tumors were harvested and protein was extracted for Western blot detection of phospho-iκBα levels compared to β-actin used a s a loading control.

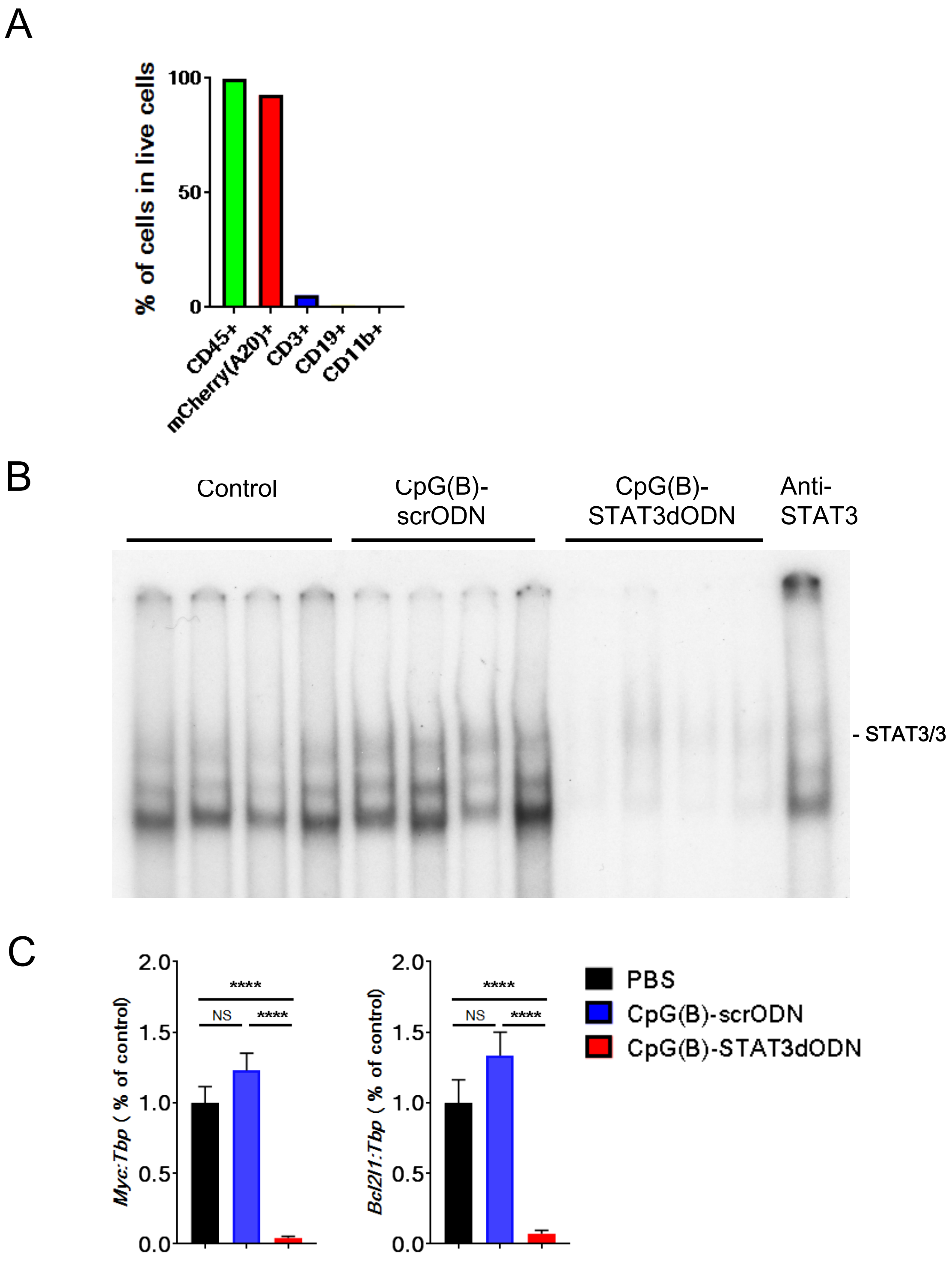

Supplemental Fig. S3. Local administration of CpG(B)-STAT3dODN inhibits STAT3 and downstream target gene expression in A20 lymphoma.

BALB/c mice were engrafted SC using 10^7 A20/mCherry/Luc cells. After tumors were established, mice treated with 1 mg/kg CpG(B)-STAT3dODN, CpG(B)-scrODN or PBS every day for 3 days. Tumors were harvested to assess cellular composition, STAT3 inhibition and downstream gene activity **(A)** Cellular composition of A20 tumors from mice before treatment as assessed using flow cytometry. Over 95% of cells are $CD45^+$/$mCherry^+$ A20 lymphoma cells with a small infiltrate of $CD3^+$ T cells and <1% of $CD45^-$ stromal cells. STAT3 activity measured using EMSA **(B)** and *c-Myc* and *Bcl2l1* expression using qPCR **(C)** after normalization to *Tbp.* Results (B,C) from one of two independent experiments; means ± SEM (*n* = 4).

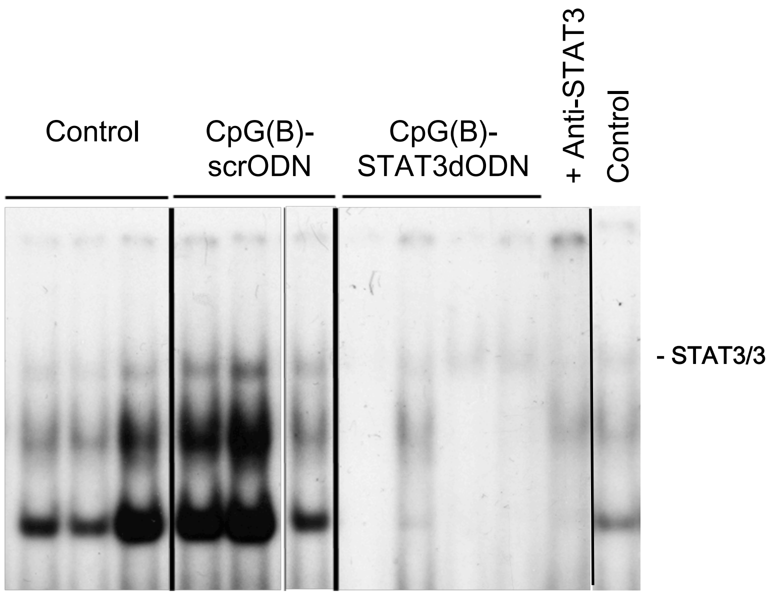

Supplemental Fig. S4. Treatment with CpG(B)-STAT3dODN inhibits STAT3 DNA binding in human B cell lymphoma *in vivo*. 10^6 of human B-cell lymphoma OCI-Ly3 cells were engrafted SC into NSG mice. After tumors were established, mice were treated using 1 mg/kg CpG(B)-STAT3dODN, control CpG(B)-scrODN or PBS every day for 3 days. Tumors were harvested for EMSA analysis of STAT3 activity using hSIE probe. The location of major STAT3-specific bands is indicated.

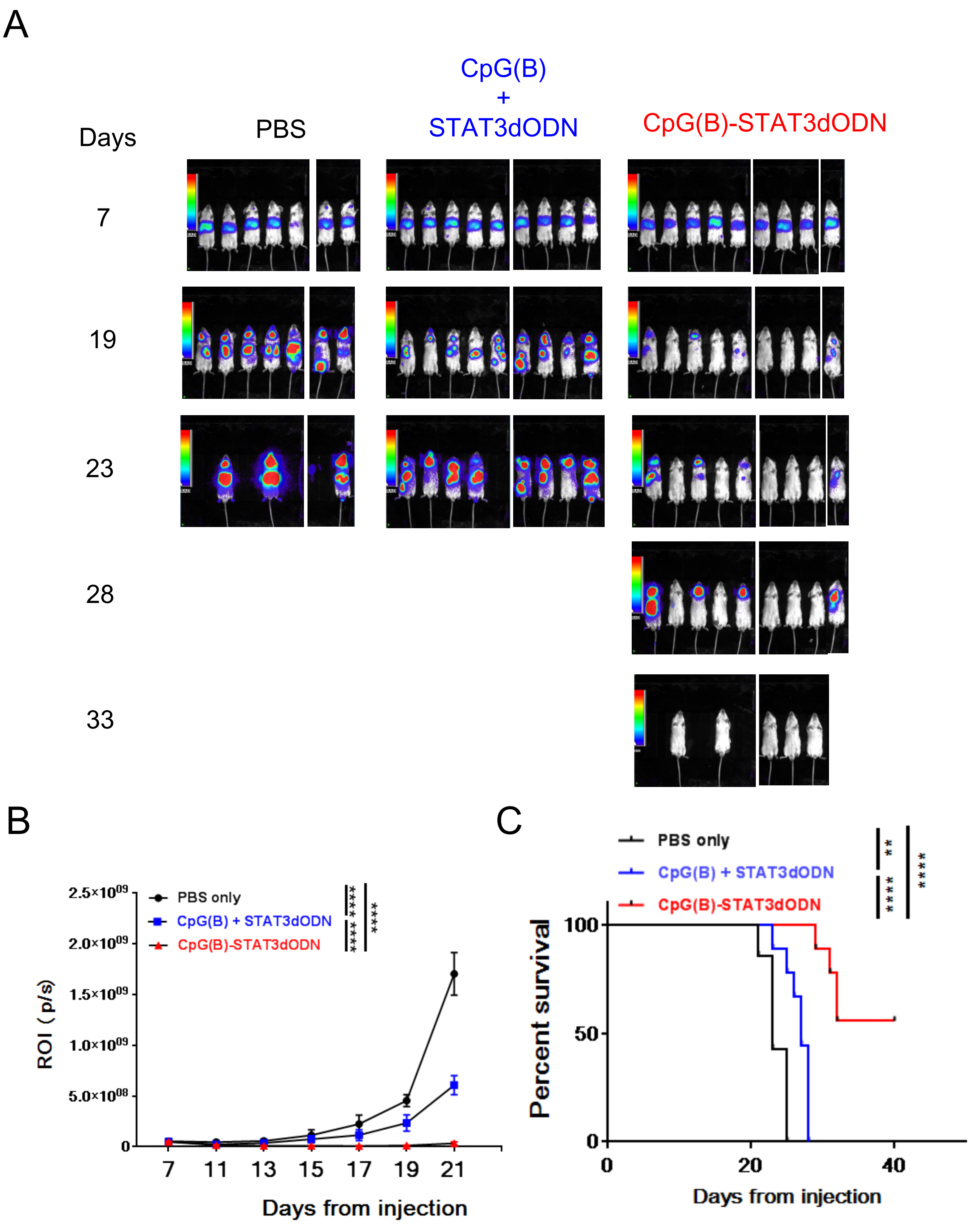

Supplemental Fig. S5. CpG(B)-STAT3dODN showed better antitumor effect *in vivo* compared with co-injection of equimolar amounts of CpG and STAT3dODN. (A) Mice with established systemic A20^{LUC} lymphoma were treated every other day starting from day 9 after injection using PBS or 5.3 nmole of each: CpG(B)-STAT3dODN, CpG(B) alone or together with equimolar amount of STAT3dODN alone; Lymphoma burden was monitored using BLI. **(B)** Tumor growth kinetics were assessed using quantification of the BLI signal during the experiment; ROI, regions of interest; p/s, photons/second; means ± SEM (*n* = 9/per group, except for PBS: *n* = 7). **(C)** CpG(B)-STAT3dODN but not CpG(B) plus STAT3dODN alone (*n* = 9/per group, except for PBS: *n* = 7) results in long-term survival of the majority of mice.

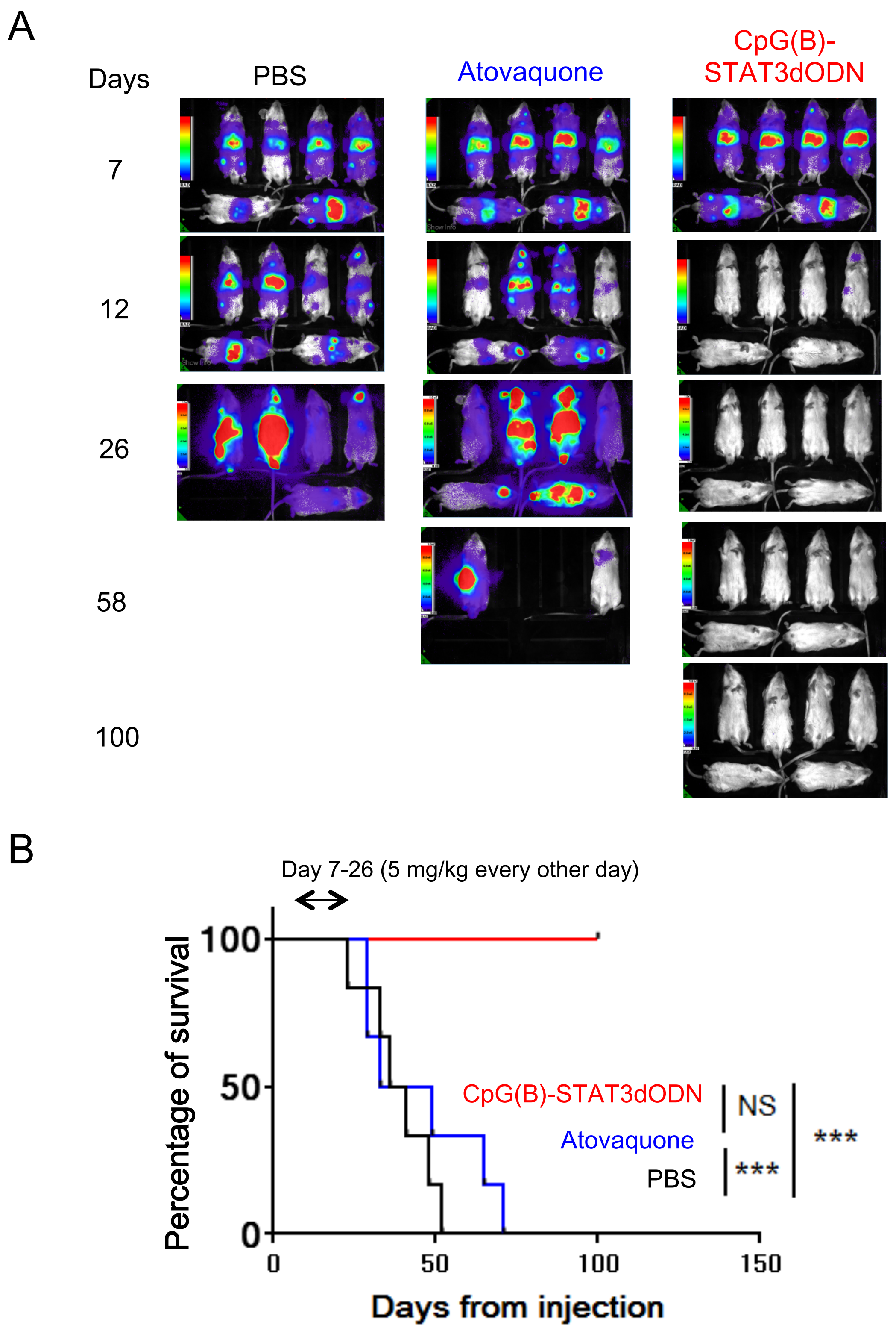

Supplemental Fig. S6. Systemic administration of CpG(B)-STAT3dODN induces better regression of syngeneic A20 B-cell lymphoma mouse model compared with the STAT3 inhibitor (atovaquone) treatment. (A) BALB/c mice with established disseminated A20^{LUC} lymphoma were treated using 5 mg/kg/every other day of CpG(B)-STAT3dODN, 200 mg/kg/day atovaquone or PBS. Lymphoma burden was monitored using BLI for up to 100 days for surviving mice. Shown are representative images from one experiments. **(B)** Treatment with CpG(B)-STAT3dODN but not atovaquone (*n* = 6/per group) leads to long-term mice survival. Shown are survival curves for the indicated treatment groups.

A

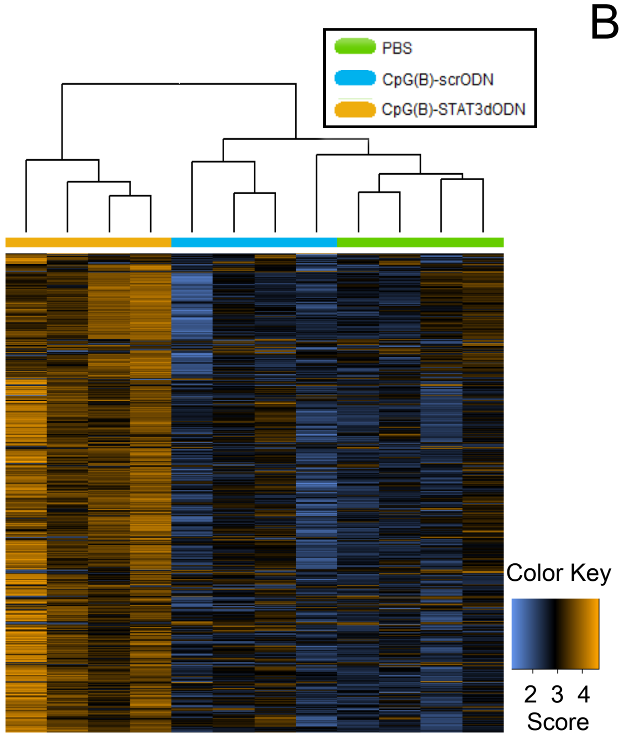

B

Signaling pathways (number of genes)	Significantly altered genes compared to PBS-treated group	
	CpG-scrODN (% coverage)	CpG-STAT3dODN (% coverage)
Cell adhesion (28)	7.1	71.4
NF-κB signaling (91)	2.2	33.0
PI3K-AKT signaling (30)	10.0	66.7
Apoptosis (45)	4.4	53.3
T-cell receptor signaling (37)	5.4	43.2
TNF signaling (63)	1.6	54.0

C

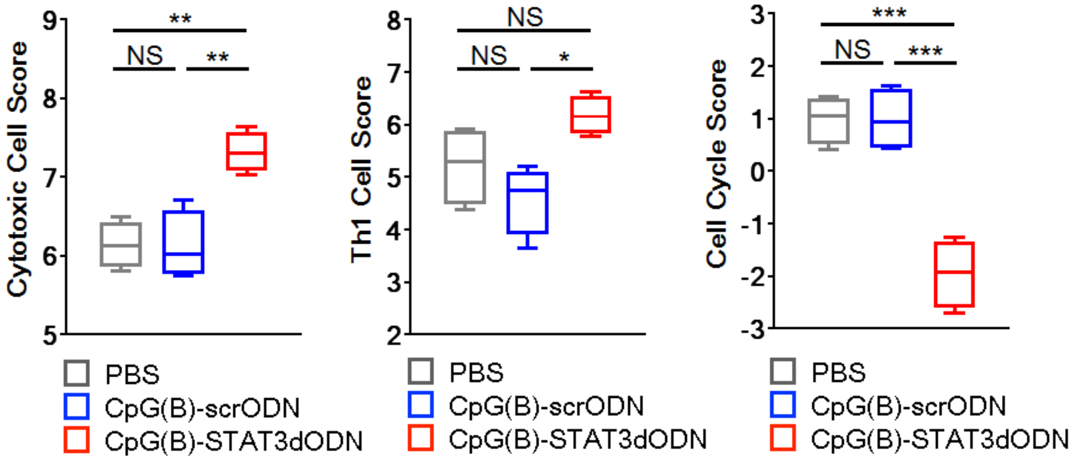

Supplemental Fig. S7. Gene expression profiling of B-cell lymphoma following TLR9 stimulation with or without concurrent STAT3 inhibition. Mice with established A20 lymphoma (SC) were treated starting on day 14 after tumor challenge using 1 mg/kg of CpG(B)-STAT3dODN, control CpG(B)-scrODN or PBS three times every other day (*n* = 4/per each group). Before tumor volumes differed, tumors were harvested to isolate total RNA for Nanostring gene expression analysis. **(A)** Comparative heat-map analysis of total gene expression changes in all samples. **(B)** Top functional pathways operating in CpG(B)-STAT3dODN- and control CpG(B)-scrODN-treated samples vs. PBS-treated controls. **(C)** Predictive scores generated by the nSolver analysis for the presence of cytotoxic cells (left), Th1 cells (middle) and the active cell cycle (right) in tested B-cell lymphoma samples; shown are means ± SEM (*n* = 4).

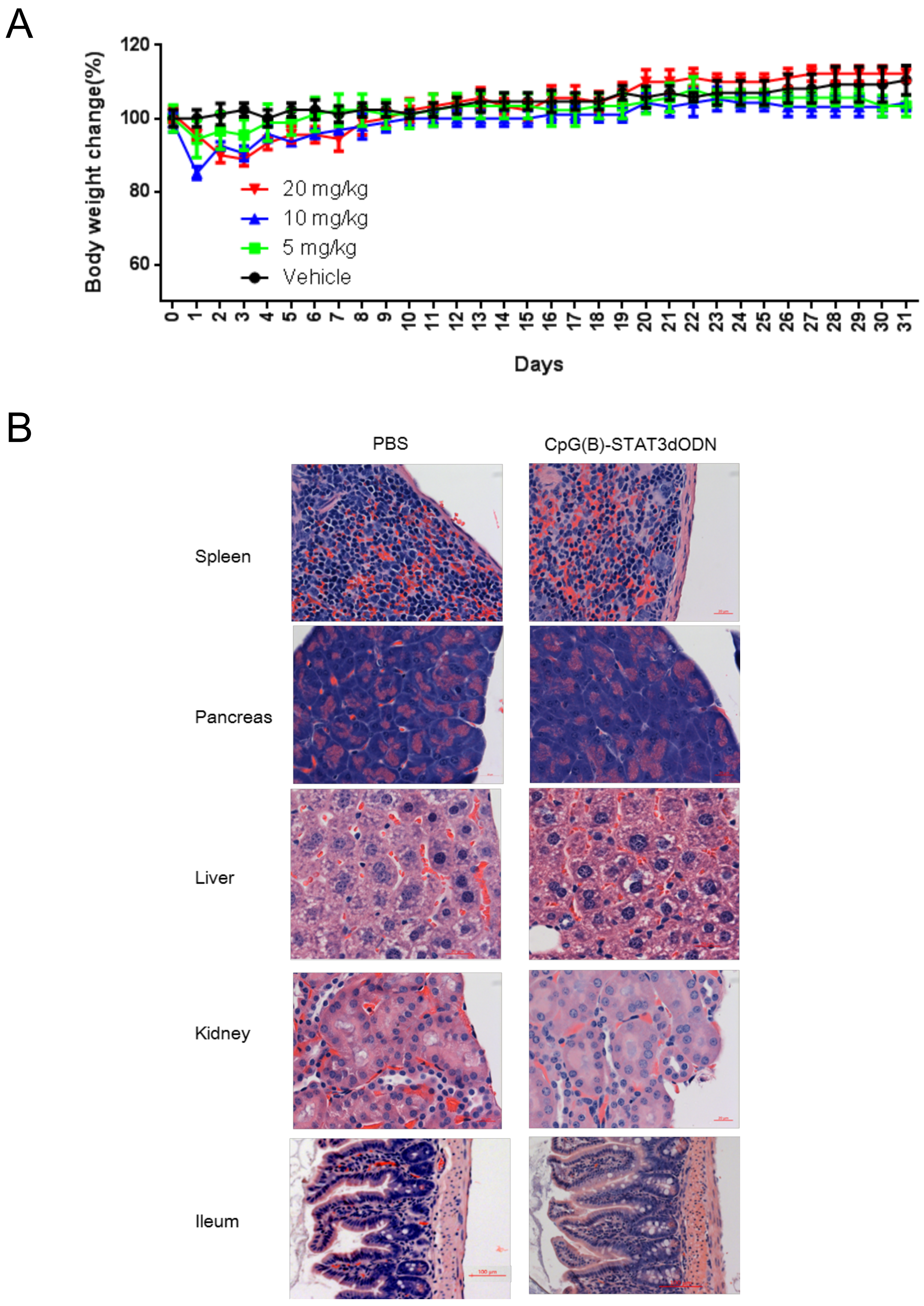

Supplemental Fig. S8. Repeated injection of CpG(B)-STAT3dODN are well tolerated in mice. (A) Balb/C mice were injected IV with 5, 10, or 20 mg/kg of CpG-STAT3dODN every other day for two weeks. The animal condition was monitored by changes in body weight compared to animal weight at the baseline (day 0) set as 100%. There were no toxicities or mortalities during the treatment and for up to 2 weeks after treatment completions. Shown are means ± SD (*n* = 4). **(B)** Repeated CpG(B)-STAT3dODN treatments did not result in gross abnormalities or inflammatory manifestations in tested organs as assessed 2 weeks after treatment completion. Histological assessment was performed on tissue sections from the highest dose group (60 mg/kg/week) vs. PBS control group. Tissue sections from the indicated organs were formalin fixed and stained using haematoxylin and eosin.

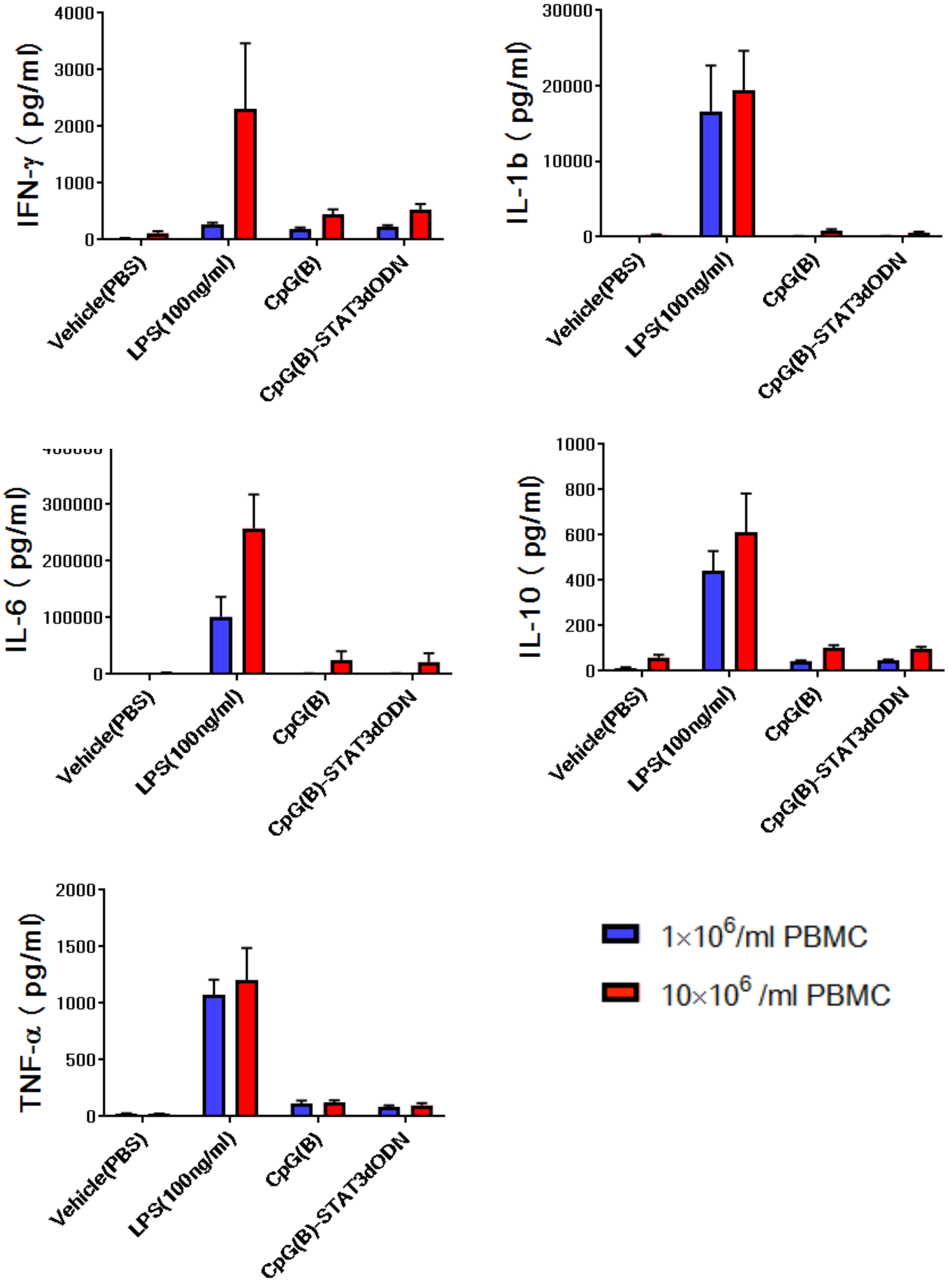

Supplemental Fig. S9. Proinflammatory cytokine production by human PBMCs cultured at low or high cell density in the presence of CpG(B)-STAT3dODN. Human PBMC isolated from from 9 healthy donors were cultured in RPMI 1640 medium (10% FBS) at standard or high density, 1 or 10 mln cells/ml, respectively. Cell were then treated using PBS, LPS (100 ng/ml), CpG(B) alone (1 μM), CpG(B)-STAT3dODN (1 μM) for 48 hours. Supernatant were collected and concentrations of immunoregulatory mediators was analyzed using Luminex assay; shown are means ± SEM (*n* = 9).
