# Supplementary material for: B Cell Lymphoma Immunotherapy Using TLR9-Targeted Oligonucleotide STAT3 Inhibitors
Source: Mol Ther. 2018 Jan 17;26(3):695–707. doi: 10.1016/j.ymthe.2018.01.007 (PMC5910676; doi:10.1016/j.ymthe.2018.01.007)
Supplement: Document S2. Article plus Supplemental Information [file mmc2.pdf]

Molecular Therapy
Original Article

B Cell Lymphoma Immunotherapy Using TLR9-Targeted Oligonucleotide STAT3 Inhibitors

Xingli Zhao,[1,2] Zhuoran Zhang,[1] Dayson Moreira,[1] Yu-Lin Su,[1] Haejung Won,[1] Tomasz Adamus,[1] Zhenyuan Dong,[3,4] Yong Liang,[5] Hongwei H. Yin,[6] Piotr Swiderski,[5] Raju K. Pillai,[6] Larry Kwak,[3,4] Stephen Forman,[3,4] and Marcin Kortylewski[1]

[1]Department of Immuno-Oncology, Beckman Research Institute, City of Hope, Duarte, CA 91010, USA; [2]State Key Laboratory of Experimental Hematology, Institute of Hematology and Blood Diseases Hospital, Chinese Academy of Medical Science and Peking Union Medical College, Tianjin 300020, China; [3]Department of Hematology and Hematopoietic Cell Transplantation, Beckman Research Institute, City of Hope, Duarte, CA 91010, USA; [4]Toni Stephenson Lymphoma Center, Department of Hematology and Hematopoietic Cell Transplantation, Beckman Research Institute, City of Hope, Duarte, CA 91010, USA; [5]DNA/RNA Synthesis Core Facility, Beckman Research Institute, City of Hope, Duarte, CA 91010, USA; [6]Molecular Pathology, Beckman Research Institute, City of Hope, Duarte, CA 91010, USA

Growing evidence links the aggressiveness of non-Hodgkin's lymphoma, especially the activated B cell-like type diffuse large B cell lymphomas (ABC-DLBCLs) to Toll-like receptor 9 (TLR9)/MyD88 and STAT3 transcription factor signaling. Here, we describe a dual-function molecule consisting of a clinically relevant TLR9 agonist (CpG7909) and a STAT3 inhibitor in the form of a high-affinity decoy oligodeoxynucleotide (dODN). The CpG-STAT3dODN blocked STAT3 DNA binding and activity, thus reducing expression of downstream target genes, such as *MYC* and *BCL2L1*, in human and mouse lymphoma cells. We further demonstrated that injections (i.v.) of CpG-STAT3dODN inhibited growth of human OCI-Ly3 lymphoma in immunodeficient mice. Moreover, systemic CpG-STAT3dODN administration induced complete regression of the syngeneic A20 lymphoma, resulting in long-term survival of immunocompetent mice. Both TLR9 stimulation and concurrent STAT3 inhibition were critical for immune-mediated therapeutic effects, since neither CpG7909 alone nor CpG7909 co-injected with unconjugated STAT3dODN extended mouse survival. The CpG-STAT3dODN induced expression of genes critical to antigen-processing/presentation and Th1 cell activation while suppressing survival signaling. These effects resulted in the generation of lymphoma cell-specific CD8/CD4-dependent T cell immunity protecting mice from tumor rechallenge. Our results suggest that CpG-STAT3dODN as a systemic/local monotherapy or in combination with PD1 blockade can provide an opportunity for treating patients with B cell NHL.

INTRODUCTION

Diffuse large B cell lymphoma (DLBCL) is the most prevalent, aggressive type of non-Hodgkin's lymphoma (NHL).[1–3] While chemo-immunotherapy improved outcome for advanced NHLs, a significant percentage of patients ultimately develops the progressive disease.[4] The STAT3 transcription factor is frequently activated in NHL, regulating cell survival and proliferation, and has been associated with poor survival of patients with aggressive lymphoma.[5–9] Mutations of upstream STAT3 regulators are common in DLBCL.[10] STAT3 activation has been linked to autocrine/paracrine stimulation by interleukin (IL)-6 in the tumor microenvironment.[11,12] More universally, STAT3 is a central immune checkpoint regulator in cancer cells and tumor-associated immune cells, such as myeloid-derived suppressor cells (MDSCs) and tumor-associated macrophages (TAMs).[13–16] While an attractive target for cancer therapy,[17–19] direct pharmacological inhibition of STAT3 proved difficult, and despite many attempts, there are still no US Food and Drug Administration (FDA)-approved small-molecule STAT3 inhibitors.[20,21] These challenges underscore the need for alternative strategies, such as oligonucleotide-based STAT3 inhibitors,[20,22] and new methods for targeted oligonucleotide delivery.[23] To overcome these obstacles, we previously developed a strategy for the delivery of therapeutic oligonucleotides, such as *STAT3* small interfering RNA (siRNA), specifically to Toll-like receptor 9$^+$ (TLR9$^+$) immune cells, such as plasmacytoid dendritic cells (pDCs), B cells,[24,25] and cancer cells.[19,26–29] TLR9 is also commonly expressed in many hematologic malignancies, including BCL.[30,31] Multiple clinical trials in NHL demonstrated safety but only limited efficacy of TLR9 agonists.[25,30] These difficulties can be, at least partly, ascribed to more recently described defects in TLR9 signaling in BCLs. Several studies have linked mutations in downstream TLR9 signaling (e.g., MYD88) or polymorphism in the *TLR9* promoter to the pathogenesis of aggressive NHL[10,32] or increased NHL incidence,[33] respectively. Based on these observations, we developed dual-function CpG-STAT3 inhibitors to generate growth-inhibitory and immuno-mediated effects against DLBCL.

Received 22 September 2017; accepted 11 January 2018;
https://doi.org/10.1016/j.ymthe.2018.01.007.

Correspondence: Marcin Kortylewski, PhD, Department of Immuno-Oncology, Beckman Research Institute, City of Hope, 1500 East Duarte Rd., Duarte, CA 91010, USA.
E-mail: mkortylewski@coh.org

This is an open access article under the CC BY license (http://creativecommons.org/licenses/by/4.0/).

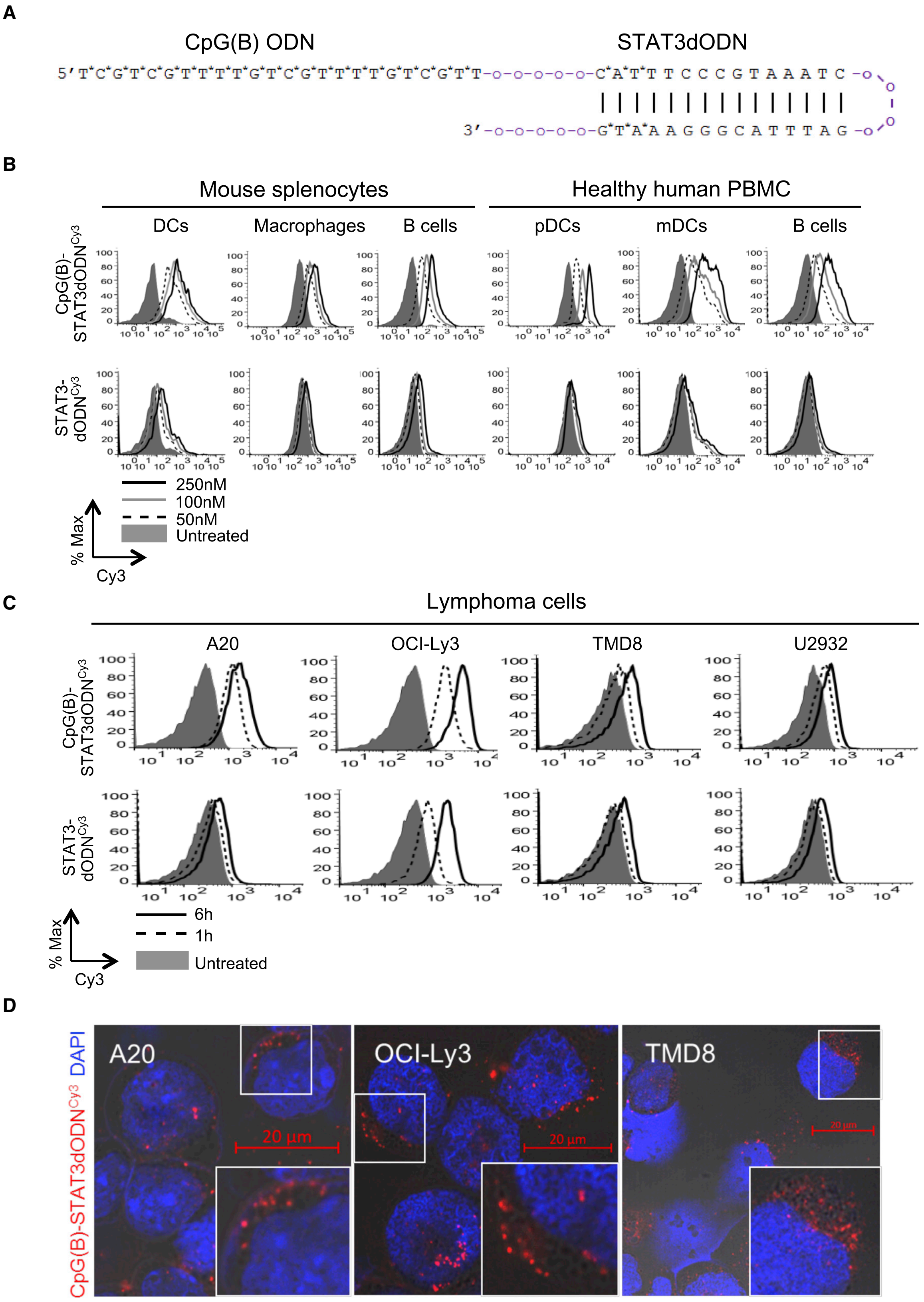

(legend on next page)

RESULTS

Optimization of the CpG-STAT3dODN Strategy for Targeting BCL Cells

We recently developed a strategy to deliver STAT3 decoy oligodeoxynucleotide inhibitor (STAT3dODN) into human myeloid cells after conjugation to the type-A TLR9 agonist, CpG ODN.[34] While CpG(A)-STAT3dODN showed efficacy in targeting a variety of myeloid cell types, it showed moderate internalization by non-malignant B cells and BCLs. To improve the targeting of BCL, we modified the targeting sequence to the well-characterized, B-type CpG7909 that was previously evaluated in clinical trials in NHL patients (Figure 1A).[35,36] More extensive phosphorothioation (PS) of the new CpG(B)-STAT3dODN improved nuclease resistance of this conjugate, which showed an 82-hr half-life in the presence of human serum (Figure S1), compared to the 63-hr half-life previously reported for CpG(A)-STAT3dODN.[34] Consistent with our previous study,[26] primary human and mouse B cells and myeloid cells quickly and efficiently internalized fluorescently labeled CpG(B)-STAT3dODN^{Cy3}, but not STAT3dODN^{Cy3} alone, even at a low 50-nM dose (Figure 1B). Furthermore, human activated B cell-like type (ABC)-DLBCL and mouse A20 lymphoma cells internalized CpG(B)-STAT3dODN^{Cy3} within 1–6 hr of incubation. The uptake of STAT3dODN alone was negligible, with the exception of the OCI-Ly3 cells, which internalized unconjugated decoy DNA, albeit less effectively (Figure 1C). Finally, we confirmed cytoplasmic localization of the CpG(B)-STAT3dODN after being internalized by target lymphoma cells, using phase-contrast and confocal microscopy (Figure 1D). Our results suggested that modified CpG(B)-STAT3dODN can effectively penetrate into immune and lymphoma cells, thereby enabling STAT3 targeting.

CpG(B)-STAT3dODN Inhibits the Transcriptional Activity of STAT3

Binding of the high-affinity decoy molecules to activated STAT3 dimers prevents downstream target gene transactivation.[20] We utilized electrophoretic mobility shift assays (EMSAs) to assess the effect of CpG(B)-STAT3dODN on STAT3 binding to a STAT3-specific radiolabeled high affinity mutant of the c-Fos sis-inducible element (hSIE) probe. As shown in Figure 2A, CpG(B)-STAT3dODN abrogated almost completely the STAT3 activity in primary mouse splenocytes and also in mouse and human BCL cells, A20 and OCI-Ly3, respectively. In contrast, both control CpG(B)-scrODN and CpG(B) ODN alone increased STAT3 activity, especially in mouse target cells, which is a known effect of TLR9 signaling. The TLR9/nuclear factor κB (NF-κB) signaling induces the expression of IL-6 and/or IL-10, which activate STAT3 to restrain immunostimulation as a negative-feedback effect.[12,37–39] We further verified that the inhibition of STAT3 activity translates into reduced expression of downstream target proteins, such as BCL-X_L and c-MYC, in human and mouse lymphoma cells.[40,41] The protein levels of BCL-X_L and c-MYC were strongly downregulated by CpG(B)-STAT3dODN but not by the unconjugated STAT3dODN or control CpG(B)-scrODN in A20 and even more pronouncedly in OCI-Ly3 lymphoma (Figure 2B). Correspondingly, CpG(B)-STAT3dODN induced dose-dependent cytotoxicity in STAT3-dependent OCI-Ly3 and TMD8 ABC-DLBCL cells *in vitro*, while it had minimal effect on SU-DHL-6 germinal center (GC)-DLBCL or A20 cells (Figure 2C; data not shown).

Next, we tested the feasibility of using this strategy for targeting STAT3 *in vivo*. Mice with established, subcutaneously engrafted (s.c.-engrafted) A20 lymphoma were treated using repeated daily intratumoral (IT) injections of 1 mg/kg CpG(B)-STAT3dODN, control CpG(B)-scrODN, or PBS. Whole tumors were harvested 1 day after the third injection to assess STAT3 activation using EMSAs. While both CpG(B)-STAT3dODN and control CpG(B)-scrODN induced similar NF-κB activity (Figure S2), only the CpG(B)-STAT3dODN reduced STAT3 DNA-binding activity in A20 tumors (Figures S3A and S3B). The observed STAT3 inhibition correlated with a suppression of target *Bcl2l1* and *Myc* mRNA in A20 cells, as verified by real-time qPCR (Figure S3C).

Local CpG(B)-STAT3dODN Treatment Inhibits Growth of Human ABC-DLBCL

We used the human ABC-DLBCL model to verify the therapeutic effect of CpG(B)-STAT3dODN in immunodeficient NSG mice. As performed similarly in A20 lymphoma (Figure S3), three IT injections of CpG(B)-STAT3dODN (1 mg/kg), but not CpG(B)-scrODN or PBS, inhibited STAT3 DNA binding in human OCI-Ly3 lymphoma, as assessed using the EMSA (Figure S4). These results were further validated using the human gene-specific Nanostring assay on the total RNA isolated from whole OCI-Ly3 tumors treated using—similarly, as before—a short-term treatment with three IT injections of 1 mg/kg CpG(B)-STAT3dODN, CpG(B)-scrODN, or PBS. The heatmap analysis demonstrated clustering of the CpG(B)-STAT3dODN gene expression pattern versus that of both CpG(B)-scrODN and PBS (Figure 3A, left). We also confirmed downregulation of known STAT3 gene targets—e.g., *ABCB1*, *BCL2L1*, *CD46*, or *MICB*—specifically in the CpG(B)-STAT3dODN group, using Nanostring and qPCR analyses (Figures 3A, middle, and 3B). Changes in the expression of genes

Figure 1. CpG(B)-STAT3dODN Design and Internalization into Specific Human and Mouse Target Cells

(A) Structure and sequence of CpG(B)-STAT3dODN synthesized chemically as a conjugate of CpG7909 ODN with a double-stranded STAT3 decoy ODN; asterisks indicate phosphorothioation sites in the oligonucleotide backbone; "o" indicates single unit of the C3 carbon chain $(CH2)_3$. (B) Dose-dependent internalization of CpG(B)-STAT3dODN^{Cy3} or the unconjugated STAT3dODN^{Cy3} by primary human peripheral blood mononuclear cells (PBMCs: $CD1c^+$/pDCs; $CD303^+$/mDCs; $CD19^+$/B cells) or mouse splenocytes ($CD11c^+$/DCs; $F4/80^+$/macrophages; $CD19^+$/B cells) after 4 hr incubation as measured using flow cytometry. (C) Uptake of 250 nM CpG(B)-STAT3dODN^{Cy3} or STAT3dODN^{Cy3} by human and mouse BCL cells after 1 or 6 hr. (D) Intracellular uptake of CpG(B)-STAT3dODN^{Cy3} by target human OCI-Ly3, TMD8, and mouse A20 lymphoma cells. Cells were incubated with 100 nM fluorescently labeled CpG7909-STAT3dODN^{Cy3} for 1 hr. The intracellular localization of the conjugate (red) and nuclei using DAPI (blue) was detected using phase contrast and confocal microscopy after 1 hr incubation. Shown are images from 1 of 3 independent experiments with similar results. Scale bars, 20 μm. % Max, percentage of maximum. $^*p < 0.05$; $^{**}p < 0.01$; $^{***}p < 0.001$.

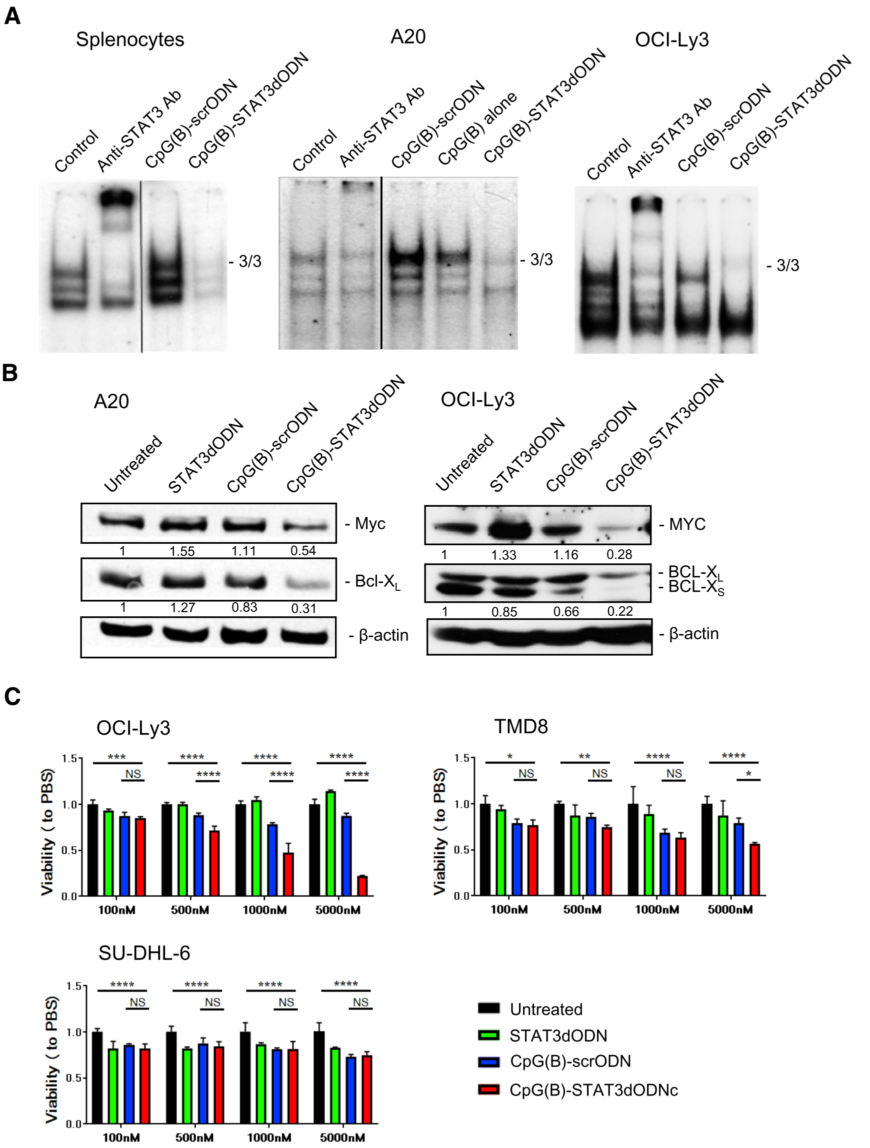

Figure 2. CpG(B)-STAT3dODN Reduces STAT3 DNA Binding and Downstream Target Gene Expression *In Vitro*

(A) Mouse splenocytes, A20 cells, or human OCI-Ly3 cells were incubated for 48 hr with CpG(B)-STAT3dODN, CpG(B)-scrODN, or CpG(B) alone (500 nM). Next, cells were lysed, and the nuclease extracts were incubated with radioactive hSIE DNA probe specific to STAT3. STAT3-specific antibodies were used to confirm band identity (supershift): S3/S3 indicates position of STAT3 homodimers. (B) A20 and OCI-Ly3 cells were incubated for 72 hr in the presence of 500 nM CpG(B)-STAT3dODN, negative control CpG(B)-scrODN, or STAT3dODN alone. The expression level of c-MYC and BCL-X_L was assessed in cell lysates using western blotting. Protein band intensities were quantified densitometrically using ImageJ v.1.46 and normalized to levels of β-actin as indicated; shown are the results from one of three independent experiments with similar outcomes. (C) Dose-dependent cytotoxic effect of CpG-STAT3dODN on STAT3-dependent human ABC-DLBCL cells. 10^5 human DLBCL cells (ABC-DLBCL: OCI-Ly3, TMD8; GC-DLBCL: SU-DHL6) per well were seeded in 96-well plate in IMDM/1% FBS medium. Cells were treated with CpG(B)-STAT3dODN or control oligonucleotides at concentrations as indicated for 3 days. The cell viability was detected using Vita-Orange Cell Viability Reagent (Biotool). STAT3-independent SU-DHL6 cells were used as a negative control. Shown are the results from one of two independent experiments performed in triplicates. Data indicate means ± SEM. *$p < 0.05$; **$p < 0.01$; ***$p < 0.001$, ****$p \leq 0.0001$; NS, not significant.

regulating proliferation (*TTK*) and cell death (*CASP8*, *FAS*, and *PBK*) suggested the onset of growth inhibition and apoptosis. In addition, we observed co-expression of immune mediators, such as interferons and their targets (*IFNA1-2*, *IFNA7-8*, *IFNB1*, and *IFNG*), as well as proinflammatory cytokines (*IL-1B* and *IL12B*) and chemokines and their receptors (*CXCL9-11* and *CXCR3*) by CpG(B)-STAT3dODN-treated lymphoma cells (Figure 3A, right). In immunodeficient NSG mice, these proinflammatory mediators of human origin could have only limited effect on innate, but not on adaptive, antitumor immunity. Nevertheless, these results suggested that the therapeutic effects of TLR9 triggering and STAT3 inhibition against BCL can be two-pronged: directly cytotoxic and immune mediated.

To assess the efficacy of systemic CpG(B)-STAT3dODN administration on OCI-Ly3 lymphoma, the immunodeficient NSG mice with established, disseminated-luciferase-expressing OCI-Ly3 lymphoma were treated using intravenous (i.v.) injections of 10 mg/kg CpG(B)-STAT3dODN, CpG(B)-scrODN, or only PBS for 10 days. The treatment with CpG(B)-STAT3dODN significantly delayed lymphoma progression in contrast to CpG(B)-scrODN control (Figures 3C and 3D). The growth inhibitory effect of CpG(B)-STAT3dODN on OCI-Ly3 lymphoma extended mouse survival versus both control groups. The effect of TLR9 stimulation alone (CpG(B)-scrODN) was relatively weak and failed to significantly affect mouse survival (Figure 3E).

Systemic Administration of CpG-STAT3dODN Triggers T Cell-Mediated BCL Regression

High nuclease resistance enables the systemic delivery of CpG(B)-STAT3dODN to disseminated BCL cells and non-malignant lymphocytes. We used fluorescently labeled CpG(B)-STAT3dODN^{Cy3} to assess oligonucleotide biodistribution. BALB/c mice with established, disseminated A20 lymphomas received a single intravenous injection of CpG(B)-STAT3dODN^{Cy3}. Peripheral blood, lymph nodes, spleen, and bone marrow were collected after 3 hr to determine CpG(B)-STAT3dODN biodistribution using flow cytometry. The internalization by A20 cells in various tissues ranged from 25% to 65% (Figure 4A).

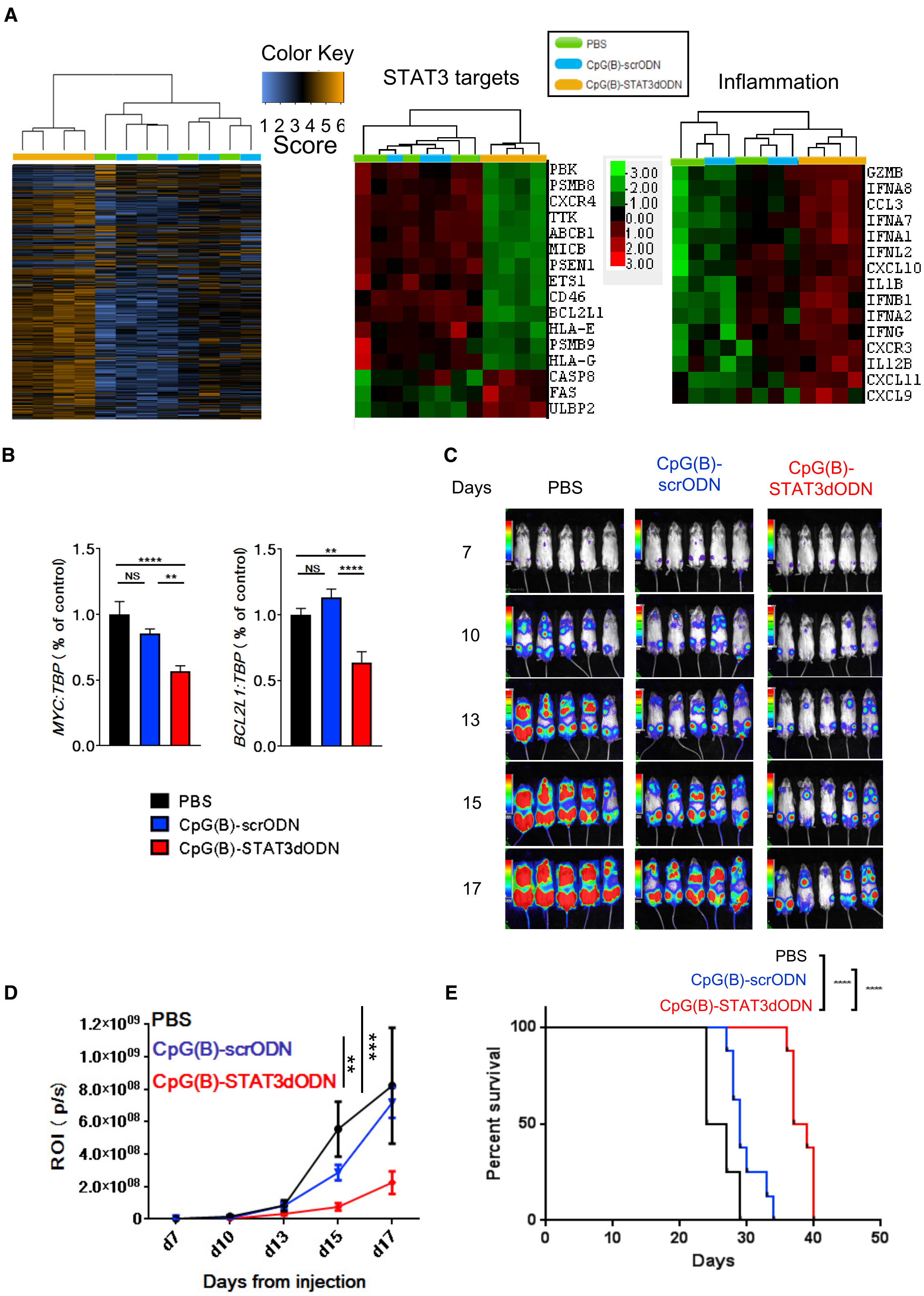

(legend on next page)

The uptake by non-malignant B cells was lower at 15%–25% and negligible for T cells in all locations, consistent with the *in vitro* uptake of CpG(B)-STAT3dODN (Figure 1B). Although intravenous injections resulted only in partial penetration of the BCL compartment, based on our previous study,[34] such an internalization rate can be sufficient for the induction of systemic antitumor immunity using CpG-STAT3 inhibitors. Thus, we next assessed the antitumor efficacy of CpG(B)-STAT3dODN administered systemically. BALB/c mice with disseminated $A20^{LUC}$ were treated using, every other day, intravenous injections of 5 mg/kg CpG(B)-STAT3dODN, CpG(B)-scrODN, or CpG(B) alone or were treated with vehicle only (PBS). Mice were treated until day 25 and then monitored without further treatment using bioluminescent imaging and body condition scoring. Within 10 days, CpG(B)-STAT3dODN treatments arrested $A20^{LUC}$ lymphoma progression (Figure 4B) and then led to complete lymphoma regression in 75% of mice (Figure 4C). In contrast, control CpG(B)-scrODN and clinically relevant CpG(B)/CpG7909 or CpG7909, co-injected in equimolar amounts with the unconjugated STAT3dODN, had minimal and only transient inhibitory effects on lymphoma progression (Figures 4B and S5, respectively). Importantly, all CpG(B)-STAT3dODN-treated mice (0/8) that survived the initial A20 challenge were resistant to the rechallenge using the same lymphoma cells, while all naive mice (8/8) showed A20 engraftment (Figure 4D). CpG(B)-STAT3dODN efficacy was also superior when compared to that of atovaquone, a recently described STAT3 inhibitor with activity against human multiple myeloma and acute myeloid leukemia (AML) (Figure S6).[42] The extended therapeutic effect of the single 2-week treatment cycle using CpG(B)-STAT3dODN and its long-term protective antitumor effect were indicative of immune-mediated antitumor responses. To assess the contribution of directly cytotoxic and immune-dependent antitumor effects, we engrafted $A20^{LUC}$ lymphoma into the immunodeficient NSG mice. Following the engraftment as verified by bioluminescent imaging (BLI), mice were treated for 2 weeks of daily injections of CpG(B)-STAT3dODN, CpG(B)-scrODN, or PBS alone (Figure 4E). The CpG(B)-STAT3dODN failed to eliminate tumors in NSG mice and only weakly improved their survival, similar to CpG(B)-scrODN. These results likely indicate the limited effect of TLR9 stimulation on innate immune cells, such as granulocytes/neutrophils still active in NSG mice,[19] while underscoring the crucial role of adaptive immune responses in generating durable lymphoma regression. We further verified the contribution of $CD8^{+}$ and $CD4^{+}$ T cells to these therapeutic effects, using antibody-mediated depletion. The $CD8^{+}$ T cell neutralization had the strongest negative impact on the CpG(B)-STAT3dODN effect against A20 lymphoma (Figure 5A). In the absence of $CD8^{+}$ T cells, lymphoma progression was accelerated compared to that of other treatment groups, and mouse survival did not differ from that of the control PBS-treated mice (Figure 5B). The CD4-specific cell depletion completely alleviated the late onset of CpG(B)-STAT3dODN-induced antitumor immunity at 2 weeks after treatment initiation (Figure 5A). However, it did not prevent the early delay in A20 lymphoma progression, which resulted in minimally extended animal survival (Figure 5B). In contrast, over half of CpG(B)-STAT3dODN-treated reference mice (CpG(B)-STAT3dODN/IgG) survived lymphoma challenge and remained tumor free for >80 days (Figure 5B). After confirming the essential role of T cell immunity in mediating CpG(B)-STAT3dODN effects *in vivo*, we assessed whether therapeutic efficacy of this strategy can be enhanced by blocking PD1/PD-L1 immune checkpoint regulation, which often results in the reduced activity of exhausted T cells. As shown in Figure 5C, systemic administration of CpG(B)-STAT3dODN or anti-PD1 antibodies as single agents showed comparable effects against A20 lymphoma, with ~50% mouse survival. Importantly, the combination of both strategies strongly improved therapeutic outcome, with 90% survival of mice (Figure 5C). These results suggest that the presence and activity of T cells are critical for the overall antitumor activity of CpG(B)-STAT3dODN against BCL.

TLR9 Stimulation and STAT3 Inhibition Prompts Immunogenicity of Lymphoma Cells

To gain insights into potential molecular mechanisms underlying the antitumor effect of CpG(B)-STAT3dODN *in vivo*, we performed Nanostring gene expression analysis on A20 BCLs. Mice with s.c.-engrafted A20 tumors were treated using three IT injections of 1 mg/kg CpG(B)-STAT3dODN, CpG(B)-scrODN, or PBS every other day. We then examined gene expression profiles in whole A20 tumors from different treatment groups. Among the 770 immune-regulation-related genes, 465 genes were significantly altered ($p < 0.05$), with 203 genes significantly upregulated more than 2-fold. The initial analysis revealed a clearly distinct transcription signature of CpG(B)-STAT3dODN-treated tumors compared to that of both vehicle- and CpG(B)-scrODN-treated groups, which clustered together, indicating a limited outcome of TLR9 triggering alone (Figure S7A). In contrast, CpG(B)-STAT3dODN resulted in the significant upregulation of signaling pathways involved in immunoregulation (Figure S7B). Further analysis indicated that TLR9 triggering and STAT3 inhibition elevated genes involved in antigen

Figure 3. CpG(B)-STAT3dODN Inhibits Growth of Disseminated Human BCL

(A and B) Local CpG(B)-STAT3dODN treatment reduces STAT3 signaling in OCI-Ly3 lymphoma xenotransplants. 1×10^{7} OCI-Ly3 cells were engrafted s.c. into NSG mice. Mice with established lymphoma were injected IT 3 times every day using 1 mg/kg CpG(B)-STAT3dODN, CpG(B)-scrODN, or PBS (n = 4). Total RNA isolated from harvested tumors was analyzed using NanoString analysis and the human-specific PanCancer Immune Profiling panel (A) and real-time qPCR (B). Data indicate means ± SEM. (C–E) Systemic administration of CpG-STAT3dODN inhibits growth of the disseminated OCI-$Ly3^{LUC}$ lymphoma. 5×10^{6} OCI-$Ly3^{LUC}$ cells were engrafted intravenously into NSG mice. Starting from day 7 after engraftment, mice were treated once daily for 10 days using injections (intravenous) of 10 mg/kg CpG(B)-STAT3dODN, CpG(B)-scrODN, or PBS alone. (C) Tumor progression was monitored using bioluminescent imaging (BLI); shown are representative images. (D) Tumor growth kinetics are indicated as assessed using quantification of the BLI signal during the experiment. ROI, regions of interest; p/s, photons per second. Data indicate means ± SEM (n = 5 per each group). (E) Systemic administration of CpG(B)-STAT3dODN extends the survival of OCI-Ly3-bearing mice. Shown are Kaplan-Meier survival curves derived from one of two independent experiments (n = 8). $^{**}p < 0.01$; $^{***}p < 0.001$, $^{****}p \leq 0.0001$; NS, not significant.

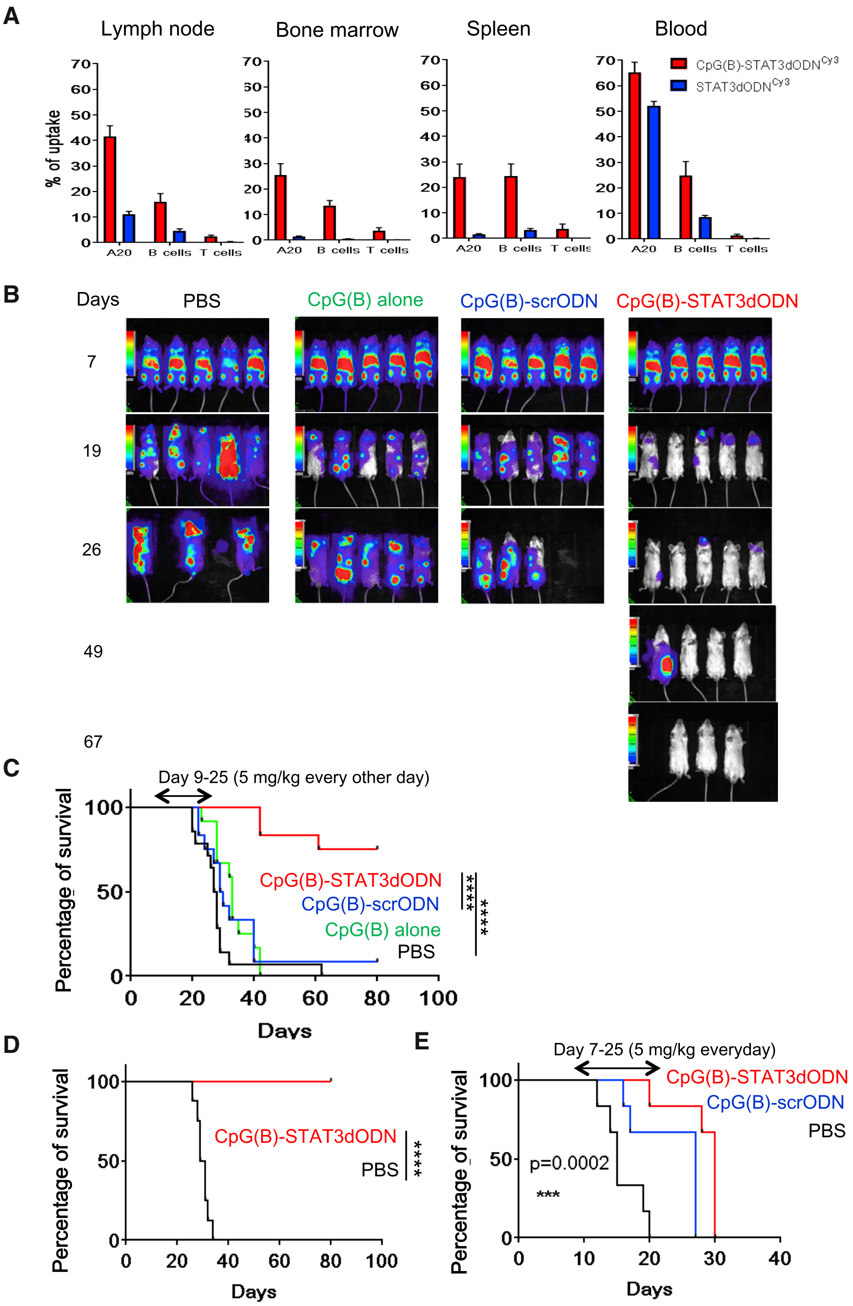

Figure 4. Systemic Administration of CpG(B)-STAT3dODN^{Cy3} Induces Regression and Protective Immunity against Syngeneic BCL in Immunocompetent Mice

(A) Biodistribution of CpG(B)-STAT3dODN^{Cy3} is shown in comparison to the unconjugated STAT3dODN^{Cy3} after a single injection intravenously (5 mg/kg) in mice bearing A20^{LUC} lymphoma. Blood and indicated organs were collected 3 hr later to assess the percentage of Cy3-positive A20 cells (CD19^{+}FSC-A^{HI}), B cells (CD19^{+}FSC-A^{LO}), or CD3^{+} T cells using flow cytometry; results are from one of two independent experiments; data indicate means ± SEM (n = 4). (B) Mice with established A20^{LUC} lymphoma were treated every other day using 5 mg/kg CpG(B)-STAT3dODN, CpG(B)-scrODN, CpG(B) alone, or PBS. Lymphoma burden was monitored using BLI; representative images are from one of two independent experiments. (C) CpG(B)-STAT3dODN, but not CpG(B)-scrODN or CpG(B) alone (n = 12 per each group), results in long-term survival of the majority of mice. Survival curves for the indicated treatment groups combine results from two independent experiments. (D) CpG(B)-STAT3dODN treatment generates long-term protective immunity. Mice that survived initial tumor engraftment resisted re-challenge with A20^{LUC} lymphoma (n = 8), compared to the naive BALB/c mice (n = 8) used as positive controls. (E) Adaptive immunity is required for the durable therapeutic effect of CpG(B)-STAT3dODN against syngeneic BCL. The immunodeficient NSG mice with established, disseminated A20 lymphoma were administered intravenous treatment every day, using 5 mg/kg CpG(B)-STAT3dODN, CpG(B)-scrODN, or PBS. Shown are survival curves for various treatment groups (n = 6 per group). ***$p < 0.001$, ****$p \leq 0.0001$.

processing/presentation, such as major histocompatibility complex (MHC) class I-related genes (e.g., *H2-D1*, *H2-K1*, *Psmb9*, *Tap1*, *Tap2*, and *Tapbp*) and class II-related genes (e.g., *H2-DM1* and *H2-DMb2*), and modulated the essential regulators of T cell activation (e.g., *Cd40/80/83/86*, *Foxp3*, *Icosl*, and *Il12a/b*) or cytotoxic functions (*Gzma/b*, *Ifnb1*, *Ifng*, and *Prf1*) (Figures 6A and 6B). Expression of the several STAT3-controlled mediators of lymphoma cell proliferation and survival, including *Bcl2l1*, *Bcl6*, *Casp3*, *Ccnd3*, and *Myc*, was reduced (Figure 6B). These gene expression profiles were reflected by significant increase of both cytotoxic cell and Th1 cell scores, with a decrease in cell cycle score, specifically in CpG(B)-STAT3dODN-treated lymphomas, but not in controls (Figure S7C).

These results prompted us to investigate whether CpG(B)-STAT3dODN can directly enhance the immunogenicity of BCL. Similarly as before, mice with s.c.-established A20 BCL were treated every other day using 1 mg/kg CpG(B)-STAT3dODN, CpG(B)-scrODN, or only PBS. A day after the fourth injection, tumors were harvested to analyze the phenotype of lymphoma cells and T cell infiltration. We first assessed phenotypic changes in surface markers related to the potential immunogenicity of A20 cells. CpG(B)-STAT3dODN, but not the control CpG(B)-scrODN, moderately enhanced the already high basal MHC class-II levels on BCL cells, but it led to a 2- to 3-fold increase in the expression of costimulatory molecules: CD80, CD86, and CD40 (Figure 7A). These changes in the phenotype of BCL cells were reflected by strongly enhanced CD8^{+} T cell infiltration of A20 lymphoma verified by immunohistochemical staining (Figure 7B). More specific flow-cytometric analysis confirmed 4- to

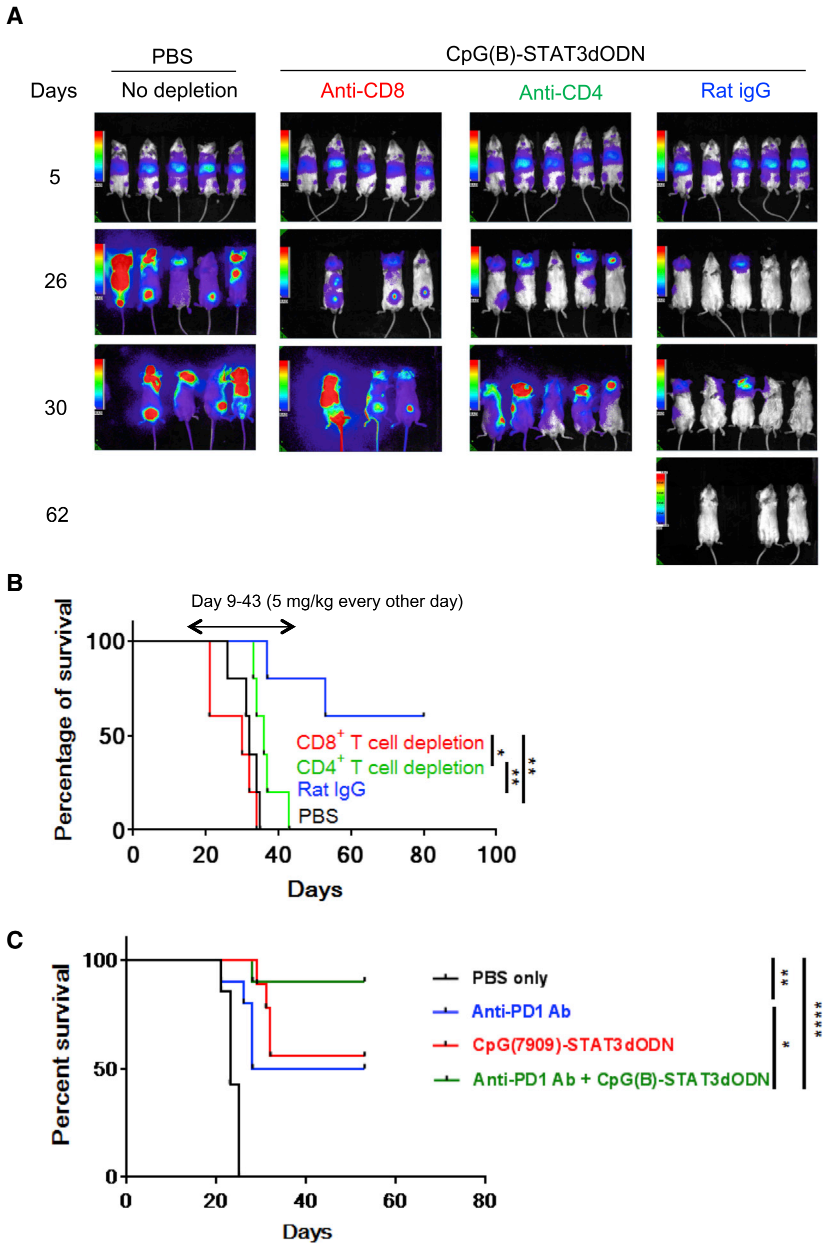

Figure 5. Therapeutic Effect of CpG(B)-STAT3dODN against BCL Relies on T Cell Activity and Can Be Augmented by Combination with PD1 Blockade

(A and B) $CD8^+$ and $CD4^+$ T cell populations were depleted in mice engrafted with $A20^{LUC}$ lymphoma using 200 μg i.p. anti-CD8 (2.43), anti-CD4 (GK1.5), or control rat IgG (Sigma-Aldrich) at day 6, day 8, and then once a week for the rest of the experiment. Starting from day 9, mice were administered an intravenous injection using 5 mg/kg CpG(B)-STAT3dODN every other day. (A) Lymphoma burden was monitored using BLI; shown are the representative images. (B) Combined results showing Kaplan-Meier survival curves for the indicated treatment groups (n = 5 per group). IgG, immunoglobulin G. (C) Mice with established systemic $A20^{LUC}$ lymphoma were treated using CpG(B)-STAT3dODN (5 mg/kg, every other day from day 9) and anti-PD1 antibody (eBioscience; 200 μg i.p.) injected alone or in combination. Kaplan-Meier survival curves are shown for the indicated treatment groups (n = 10 per group, except for PBS: n = 7). $^*p < 0.05$, $^{**}p < 0.01$, $^{****}p \leq 0.0001$.

5-fold increases in the percentage of tumor-infiltrating $CD8^+$ and $CD4^+$ T cells, with concomitant reduction in the percentage of $CD4^+$/$FoxP3^+$ regulatory T cells (Tregs). These changes produced an average 12-fold increase in the ratio of $CD8^+$ to Tregs, which can be indicative of an effective, adaptive antitumor immune response (Figure 7C). Finally, we verified whether CpG(B)-STAT3dODN-induced immune responses are specific for A20 BCL antigens. The irradiated A20 cells were used as a source of tumor antigens to trigger recall antitumor responses in T cells isolated from the lymph nodes of A20-lymphoma-bearing mice following treatment using CpG(B)-STAT3dODN, CpG(B)-scrODN, or PBS. The ELISPOT assay detected highly elevated numbers of IFNγ-secreting lymphocytes from CpG(B)-STAT3dODN-treated mice, compared to a significant but weak response to CpG(B)-scrODN treatment, versus PBS control mice (Figure 7D). Despite the potent antitumor efficacy of CpG(B)-STAT3dODN *in vivo*, we did not observe toxicities or autoimmune manifestations in immunocompetent mice over the 2-week treatment. Toxicology studies and histopathological analyses, in acute and chronic settings, failed to detect dose-limiting toxicities or organ gross abnormalities in CpG(B)-STAT3dODN-treated mice for up to 60 mg/kg/week (Figure S8). These results confirm that CpG(B)-STAT3dODN can be safe and well tolerated during repeated systemic administration. Finally, the immunostimulatory effect of CpG(B)-STAT3dODN on human PBMCs at low or high cell density was moderate, comparable with the effect of CpG(B) alone, and did not result in a "cytokine storm" (Figure S9).[43] Overall, our results support the feasibility and safety of using the combination of TLR9 stimulation with STAT3 inhibition for treatment of BCLs. The CpG(B)-STAT3dODN strategy has the potential to augment therapeutic effects by combining direct cytotoxicity with strong $CD8^+$ and $CD4^+$ T cell-mediated antitumor immunity.

DISCUSSION

In this study, we demonstrate that a conjugate of STAT3 decoy inhibitor with TLR9 ligand, CpG(B)-STAT3dODN, provides an *in vivo* strategy for cell-selective delivery to BCL cells. Without the need for pharmacological formulation, the unformulated CpG(B)-STAT3dODN, injected locally or systemically, was effective in blocking STAT3 in lymphoma cells. Repeated treatment with CpG(B)-STAT3dODN had a therapeutic effect against established

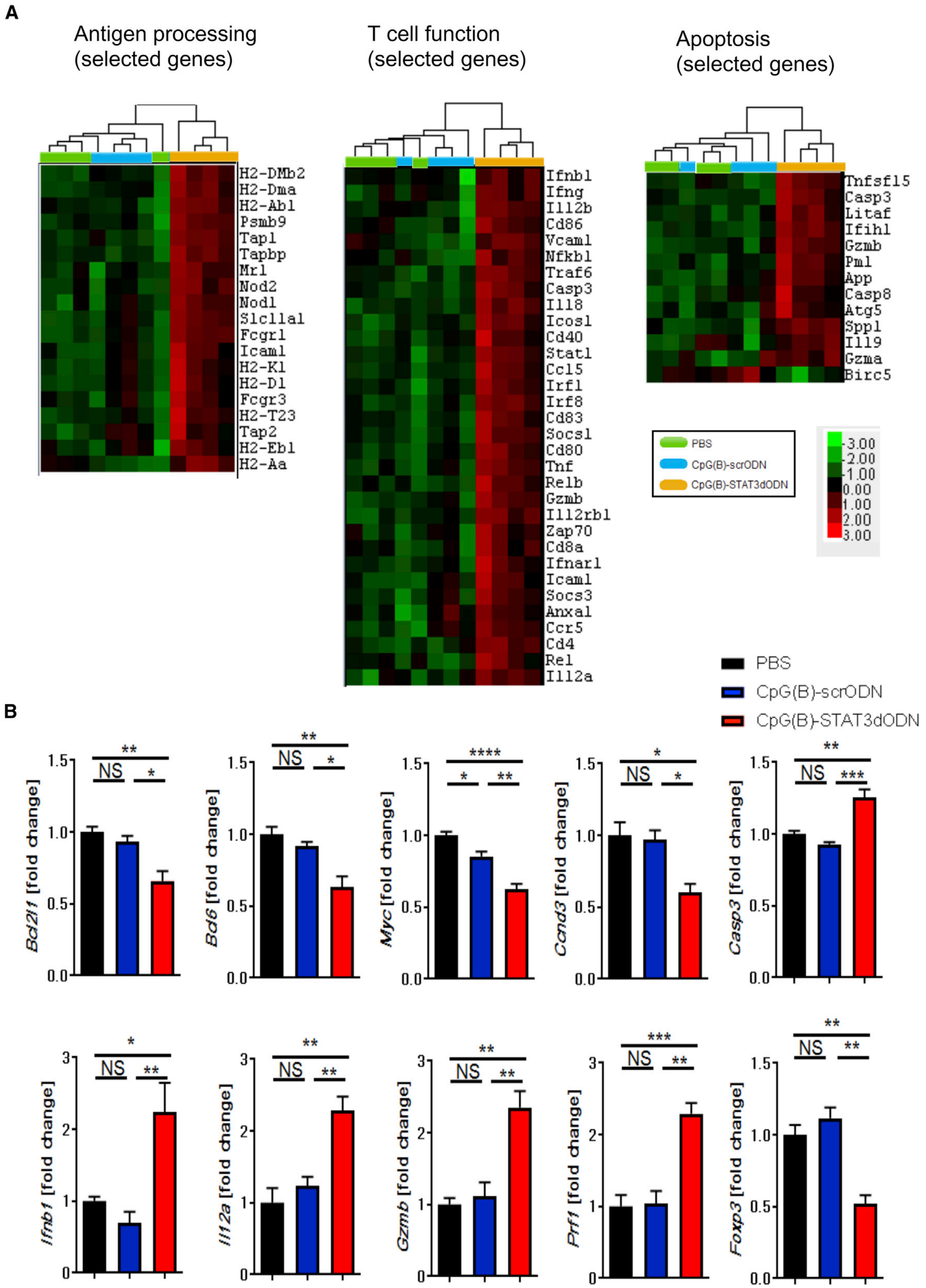

(legend on next page)

human DLBCL xenotransplants, inhibiting their growth by a direct cytotoxic/cytostatic effect. While the therapeutic efficacy of CpG(B)-STAT3dODN in immunocompetent mice relied mainly on the potent T cell-mediated antitumor immunity, it was, at least partly, supported by the direct antitumor effect. The exact contribution of indirect versus direct antitumor effects will be explored in our further studies in TLR9-deficient lymphoma models. Importantly, the dual-function antitumor effects required the combination of TLR9 agonist (CpG ODN) with STAT3 inhibitor in one molecule and were not observed for CpG7909 ODN or STAT3 inhibitor when injected alone or even when co-injected. The STAT3 inhibition, through the reversible decoy effect, combined with concurrent TLR9 activation seemed sufficient for the induction of the lymphoma differentiation to antigen-presenting phenotype, thereby ensuring therapeutic antitumor efficacy.

Previous studies by other groups demonstrated that TLR9 agonists—such as CpG7909, used in our study—inhibit the growth of cultured BCL cells and stimulate their immunogenicity.[25,30,44] Preclinical studies in mice suggested that the increased immunogenicity of BCL cells can contribute to systemic antitumor immune responses generated by local administration of CpG ODNs, creating an effect of *in situ* vaccination.[45] However, these effects required 5- to 10-fold higher concentrations of TLR9 agonists and direct IT injections to achieve antitumor effects.[44,46,47] To overcome these limitations, TLR9 agonists in preclinical studies, and later in clinical trials, were combined with either chemotherapy, radiotherapy, or targeted antibody-based strategies (e.g., rituximab).[25,47,48] Our previous studies and current observations suggest that blocking STAT3 removes restraints on the potency of TLR9-driven immunostimulation.[26,28,38,49] This is in agreement with an original report in mouse lymphoma cells that suggested the role of STAT3 in restricting BCL cell immunogenicity.[50] Importantly, while mutations may prevent direct effects of STAT3 inhibition on BCL cells, the increased activity of non-malignant antigen-presenting cells can be sufficient for the induction of antitumor immune responses.[51] Several small-molecule JAK inhibitors and STAT3 antisense oligonucleotides are currently in clinical trials for hematologic malignancies, including drug-resistant or relapsed BCL cells.[22,52] Whether their efficacy partly relies on immune-mediated effects is not yet clear. The non-selective, systemic Jak/STAT inhibition potentially might impede IFN-mediated antitumor T cell immunity and/or generation of memory T cells.[11] In contrast, emerging clinical immunotherapies activate T cells by directly blocking immune checkpoint molecules, such as PD1, responsible for T cell exhaustion. Given that targeting PD1 has already shown promise in initial phase I/II clinical trials in patients with advanced-stage NHL, including DLBCL, these strategies are likely to benefit from the combination with immunogenic CpG(B)-STAT3dODN.[52] Therefore, TLR9-targeted delivery of STAT3 inhibitors, as single agents or in combination with T cell-based approaches, offers a novel strategy for safer and more effective immunotherapy of BCL and, potentially, other hematologic malignancies.

MATERIALS AND METHODS

Cells

PBMCs from anonymous healthy donors were collected in accordance with the Declaration of Helsinki under the institutional review board (IRB) protocol 13378 (City of Hope; COH).[19] Cell viability was >90%, as confirmed using flow cytometry. Mouse A20 and human OCI-Ly3 cells were from ATCC or DSMZ, respectively, and TMD8 and U2932 ABC-DLBCL cells were kindly provided by Dr. G. Inghirami (Weill Cornell Medicine, New York, NY, USA). Cells were cultured in RPMI 1640/10% fetal bovine serum (FBS) media. To generate A20^{LUC} and OCI-Ly3^{LUC} cells, parental cells were transduced with *luciferase/mCherry* using a lentiviral vector.[53]

Mice

All animal experiments were followed established institutional guidance and approved protocols from the institutional animal care and use committee (COH). BALB/c mice were purchased from the National Cancer Institute (Frederick, MD, USA). *NOD/SCID/IL-2RγKO* (NSG) mice, originally from the Jackson Laboratory, were maintained at the COH. Mice were injected intravenously or s.c. with 5×10^6 OCI-Ly3^{LUC} or A20^{LUC} in PBS, and lymphoma engraftment/progression was monitored using BLI on the AmiX (Spectral Instruments).

Oligonucleotides

All oligonucleotides were synthesized in the DNA/RNA Synthesis Core (COH) by linking CpG7909 to STAT3dODN in a manner similar to one previously described.[26] The resulting conjugates are shown below (x = single C3 unit; asterisks indicate phosphorothioation sites):

CpG(B)-STAT3dODN

5′-T*C*G*T*C*G*T*T*T*T*G*T*C*G*T*T*T*T*G*T*C*G*T*T-xxxxx-C*A*T*TTCCCGTAAATC-xxxx-GATTTACGGGAA*A*T*G-xxxxx-3′

CpG(B)-scrambledODN

5′-T*C*G*T*C*G*T*T*T*T*G*T*C*G*T*T*T*T*G*T*C*G*T*T -xxxxx-A*C*T*CTTGCCAATTAC-xxxx-GTAATTGGCAAG*A*G*T-xxxxx-3′

Figure 6. Gene Expression Profiling of BCL following TLR9 Stimulation with or without Concurrent STAT3 Inhibition

Mice with s.c.-established A20 lymphoma were treated starting on day 14 after tumor challenge using 1 mg/kg CpG(B)-STAT3dODN, control CpG(B)-scrODN, or PBS three times every other day (n = 4 per each group). Before tumor volumes differed, tumors were harvested to isolate total RNA for NanoString analysis using the mouse-specific PanCancer Immune Profiling panel. (A) Heatmap analyses for selected functional groups of genes related to antigen processing (left), T cell function (middle), and apoptosis (right). (B) Graph bars representing expression of specific targets from apoptosis- and T cell function-related groups; data indicate means ± SEM (n = 4). *$p < 0.05$, **$p < 0.01$, ***$p < 0.001$, ****$p \leq 0.0001$; NS, not significant.

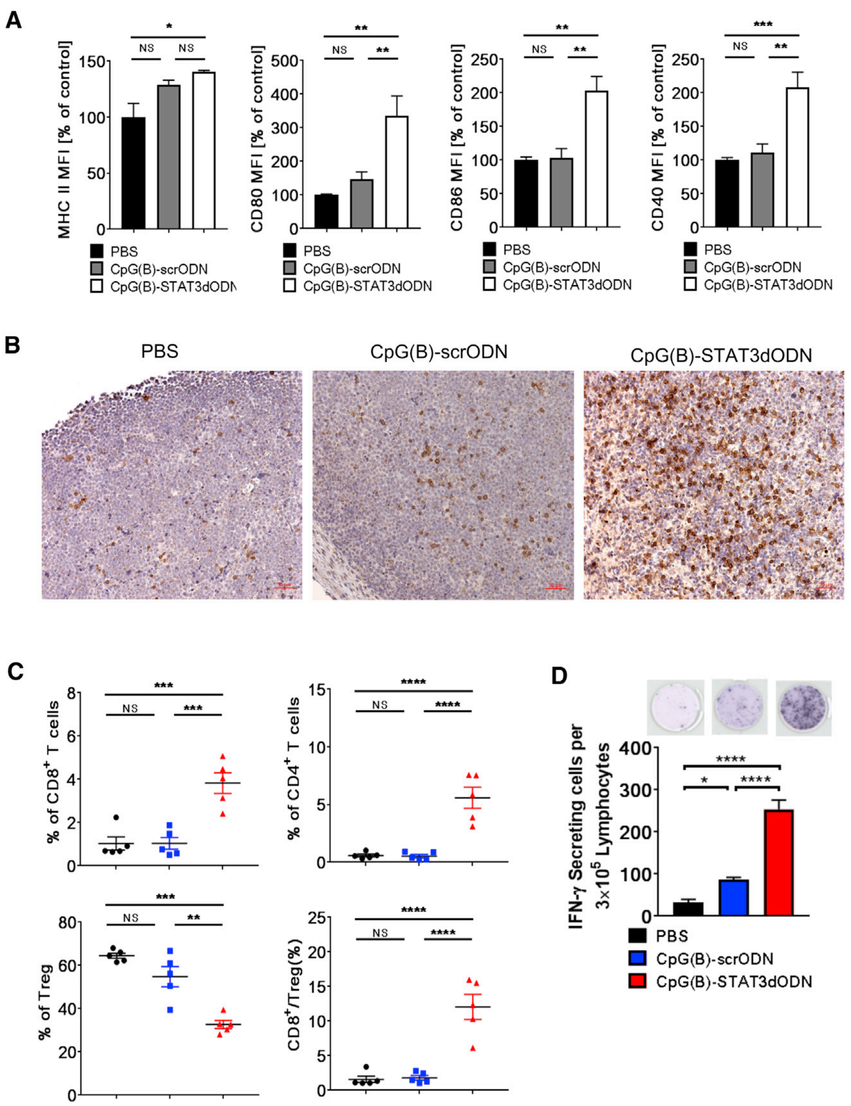

Figure 7. CpG(B)-STAT3dODN Treatment Results in B Cell-Lymphoma-Specific T Cell Immune Responses with Tumor Infiltration by CD8+ T Cells

Mice with s.c.-established A20 lymphoma were injected IT using 1 mg/kg CpG(B)-STAT3dODN, CpG-scrODN, or PBS 4 times every other day. Tumors were harvested and dispersed into single-cell suspensions for the phenotypic immune marker analysis using flow cytometry. (A) CpG(B)-STAT3dODN upregulates surface expression of MHC class-II, costimulatory CD40, CD80, and CD86 molecules. Data indicate means ± SEM (n = 5) from one of two independent experiments with similar outcomes. (B) $CD8^+$ T cell infiltration in A20 lymphoma tumors visualized using immunohistochemical staining on FFPE tumor sections. Scale bars, 50 μm. (C) The infiltration by various T cell populations in A20 tumors were assessed using flow cytometry. Shown are percentages of total $CD3^+CD8^+$, $CD3^+CD4^+$ T cells, and $CD3^+CD4^+Foxp3^+$ regulatory T cells (Tregs), as well as the ratio of $CD3^+CD8^+$ T cells to Tregs. (D) TLR9 stimulation combined with STAT3 inhibition generates A20 B cell-lymphoma-specific immune responses. Recall response to irradiated (100 Gy) A20 cells was assessed using single-cell suspensions prepared from tumor-draining lymph nodes from *in vivo*-treated mice as described above. Numbers of IFNγ-secreting cells were assessed using an ELISPOT assay. Upper panel: representative images; bottom panel: bar graphs representing results from one of two independent experiments. Data indicate means ± SEM (n = 5). $^*p < 0.05$, $^{**}p < 0.01$, $^{***}p < 0.001$, $^{****}p \leq 0.0001$; FFPE, formalin-fixed, paraffin embedded; MFI, mean fluorescence intensity; NS, not significant.

STAT3dODN

5′-C*A*T*TTCCCGTAAATC-xxxx-GATTTACGGGAA*A*T*G-xx xxx-3′

CpG(B/7909)

5′-T*C*G*T*C*G*T*T*T*T*G*T*C*G*T*T*T*T*G*T*C*G*T*T-3′

For internalization/biodistribution studies, oligonucleotides were 3′-labeled using Cy3 fluorochrome.

EMSAs and Protein Detection

EMSAs to detect STAT3 DNA-binding activity were performed as described previously.[26] Briefly, 10 μg nuclear extracts were incubated with hSIE ^{32}P-labeled oligonucleotide probes specific to STAT3 (hSIE)[54] for supershift control using anti-STAT3 antibody (Santa Cruz Biotechnology). Western blot detection to detect STAT3, pSTAT3, BCL-X_L, c-Myc, and β-actin expression was performed as described earlier.[19]

Cell Viability Assay

10^4 DLBCL lymphoma cell lines were seeded in 100 μL 1% FBS Iscove's Modified Dulbecco's Medium (IMDM) in a 96-well plate and incubated in the presence of designated reagent for 3 days. 10 μL Vita-Orange Cell Viability Reagent (Biotool, B34304) was added into each well, and the mixture was incubated at 37°C with 5% CO_2 for 4 hr. Optical density (OD) at 450 nm was detected using Citation3, with OD at 690 nm as reference.

Immune Assays

For extra-/intracellular staining, fluorochrome-labeled antibodies and previously described staining protocols were used (eBioscience).[17,29] Data collected on the BD-Accuri C6 and BD-Fortessa (BD Biosciences) were analyzed using FlowJo software (TreeStar). The ELISPOT assay was performed following manufacturer's protocol (CellSciences), as described previously.[28] Immunohistochemistry using anti-CD8 antibody (eBioscience/4SM15) was performed as described previously[19] and was analyzed on the Observer II microscope (Zeiss). For confocal microscopy, cells were fixed in 2% paraformaldehyde (Electron Microscopy Sciences); then, nuclei were stained using DAPI (Sigma-Aldrich),

and slides were analyzed using an inverted confocal microscope (LSM880-Airyscan; Zeiss) and the LSM Image Browser (Zeiss, v.4.2.0.121).[27]

NanoString Analysis

Total RNA was extracted from whole OCI-Ly3 or A20 tumors using the mirVana miRNA Isolation Kit (Ambion). RNA quality was verified using the Bioanalyzer-2100 (Agilent). Gene expression was analyzed using the PanCancer Immune Profiling panel for human or mouse, XT-CSO-HIP1-12 or XT-CSO-MIP1-12, respectively, on the nCounter system (NanoStringTechnologies) following the manufacturer's recommendations. Results were analyzed using nSolver 3.0 software (NanoStringTechnologies) and automated normalization of raw data.

Statistical Analysis

An unpaired t test was used to calculate the two-tailed p value to estimate statistical significance of differences between two experimental groups. A one-way ANOVA plus Bonferroni posttest were applied to assess the statistical significance of differences between multiple treatment groups. The relationship between two groups was assessed using correlation and linear regression. The p and r^2 values are indicated in the figures with asterisks: $^*p < 0.05$; $^{**}p < 0.01$; $^{***}p < 0.001$. Data were analyzed using Prism v.6.03 software (GraphPad).

SUPPLEMENTAL INFORMATION

Supplemental Information includes Supplemental Materials and Methods and nine figures can be found with this article online at https://doi.org/10.1016/j.ymthe.2018.01.007.

AUTHOR CONTRIBUTIONS

Study design: M.K. and X.Z.; Conducting experiments: X.Z., Z.Z., D.M., Y.-L.S., H.W., and T.A.; Data analysis/interpretation: M.K., S.F., L.K., X.Z., Z.Z., H.H.Y., and R.K.P.; Providing reagents: Y.L., Z.D., P.S., and L.K.; Writing manuscript: Z.Z., X.Z., and M.K.

ACKNOWLEDGMENTS

We are grateful to Dr. Giorgio Inghirami (Weill Cornell Medicine, New York, NY, USA) for providing DLBCL cell lines and advice and to the staff at Analytical Cytometry, Pathology, Animal Resource Cores (COH). This work was supported in part by the National Cancer Institute/NIH awards R01CA213131 (to M.K.), P50CA107399 (to S.F.), and P30CA033572 (to the COH) and by the STOP-CANCER Foundation (to M.K.). The content is solely the responsibility of the authors and does not necessarily represent the official views of the NIH.

REFERENCES

1. Siegel, R.L., Miller, K.D., and Jemal, A. (2016). Cancer statistics, 2016. CA Cancer J. Clin. *66*, 7–30.
2. Glass, A.G., Karnell, L.H., and Menck, H.R. (1997). The National Cancer Data Base report on non-Hodgkin's lymphoma. Cancer *80*, 2311–2320.
3. Scott, D.W., and Gascoyne, R.D. (2014). The tumour microenvironment in B cell lymphomas. Nat. Rev. Cancer *14*, 517–534.
4. Amin, A.D., Peters, T.L., Li, L., Rajan, S.S., Choudhari, R., Puvvada, S.D., and Schatz, J.H. (2017). Diffuse large B-cell lymphoma: can genomics improve treatment options for a curable cancer? Cold Spring Harb. Mol. Case Stud. *3*, a001719.
5. Baran-Marszak, F., Boukhiar, M., Harel, S., Laguillier, C., Roger, C., Gressin, R., Martin, A., Fagard, R., Varin-Blank, N., Ajchenbaum-Cymbalista, F., and Ledoux, D. (2010). Constitutive and B-cell receptor-induced activation of STAT3 are important signaling pathways targeted by bortezomib in leukemic mantle cell lymphoma. Haematologica *95*, 1865–1872.
6. Ding, B.B., Yu, J.J., Yu, R.Y., Mendez, L.M., Shaknovich, R., Zhang, Y., Cattoretti, G., and Ye, B.H. (2008). Constitutively activated STAT3 promotes cell proliferation and survival in the activated B-cell subtype of diffuse large B-cell lymphomas. Blood *111*, 1515–1523.
7. Frank, D.A. (2013). Transcription factor STAT3 as a prognostic marker and therapeutic target in cancer. J. Clin. Oncol. *31*, 4560–4561.
8. Huang, X., Meng, B., Iqbal, J., Ding, B.B., Perry, A.M., Cao, W., Smith, L.M., Bi, C., Jiang, C., Greiner, T.C., et al. (2013). Activation of the STAT3 signaling pathway is associated with poor survival in diffuse large B-cell lymphoma treated with R-CHOP. J. Clin. Oncol. *31*, 4520–4528.
9. Ok, C.Y., Chen, J., Xu-Monette, Z.Y., Tzankov, A., Manyam, G.C., Li, L., Visco, C., Montes-Moreno, S., Dybkær, K., Chiu, A., et al. (2014). Clinical implications of phosphorylated STAT3 expression in de novo diffuse large B-cell lymphoma. Clin Cancer Res. *20*, 5113–5123.
10. Ngo, V.N., Young, R.M., Schmitz, R., Jhavar, S., Xiao, W., Lim, K.H., Kohlhammer, H., Xu, W., Yang, Y., Zhao, H., et al. (2011). Oncogenically active MYD88 mutations in human lymphoma. Nature *470*, 115–119.
11. Hillmer, E.J., Zhang, H., Li, H.S., and Watowich, S.S. (2016). STAT3 signaling in immunity. Cytokine Growth Factor Rev. *31*, 1–15.
12. Lam, L.T., Wright, G., Davis, R.E., Lenz, G., Farinha, P., Dang, L., Chan, J.W., Rosenwald, A., Gascoyne, R.D., and Staudt, L.M. (2008). Cooperative signaling through the signal transducer and activator of transcription 3 and nuclear factor-κB pathways in subtypes of diffuse large B-cell lymphoma. Blood *111*, 3701–3713.
13. Gabrilovich, D.I., Ostrand-Rosenberg, S., and Bronte, V. (2012). Coordinated regulation of myeloid cells by tumours. Nat. Rev. Immunol. *12*, 253–268.
14. Kusaba, H., Ghosh, P., Derin, R., Buchholz, M., Sasaki, C., Madara, K., and Longo, D.L. (2005). Interleukin-12-induced interferon-gamma production by human peripheral blood T cells is regulated by mammalian target of rapamycin (mTOR). J. Biol. Chem. *280*, 1037–1043.
15. Yu, H., Kortylewski, M., and Pardoll, D. (2007). Crosstalk between cancer and immune cells: role of STAT3 in the tumour microenvironment. Nat. Rev. Immunol. *7*, 41–51.
16. Yu, J., Wang, Y., Yan, F., Zhang, P., Li, H., Zhao, H., Yan, C., Yan, F., and Ren, X. (2014). Noncanonical NF-κB activation mediates STAT3-stimulated IDO upregulation in myeloid-derived suppressor cells in breast cancer. J. Immunol. *193*, 2574–2586.
17. Kortylewski, M., Kujawski, M., Wang, T., Wei, S., Zhang, S., Pilon-Thomas, S., Niu, G., Kay, H., Mulé, J., Kerr, W.G., et al. (2005). Inhibiting Stat3 signaling in the hematopoietic system elicits multicomponent antitumor immunity. Nat. Med. *11*, 1314–1321.
18. Welte, T., Zhang, S.S., Wang, T., Zhang, Z., Hesslein, D.G., Yin, Z., Kano, A., Iwamoto, Y., Li, E., Craft, J.E., et al. (2003). STAT3 deletion during hematopoiesis causes Crohn's disease-like pathogenesis and lethality: a critical role of STAT3 in innate immunity. Proc. Natl. Acad. Sci. USA *100*, 1879–1884.
19. Zhang, Q., Hossain, D.M., Nechaev, S., Kozlowska, A., Zhang, W., Liu, Y., Kowolik, C.M., Swiderski, P., Rossi, J.J., Forman, S., et al. (2013). TLR9-mediated siRNA delivery for targeting of normal and malignant human hematopoietic cells in vivo. Blood *121*, 1304–1315.
20. Sen, M., and Grandis, J.R. (2012). Nucleic acid-based approaches to STAT inhibition. JAK-STAT *1*, 285–291.
21. Szeląg, M., Czerwoniec, A., Wesoly, J., and Bluyssen, H.A. (2014). Comparative screening and validation as a novel tool to identify STAT-specific inhibitors. Eur. J. Pharmacol. *740*, 417–420.

22. Hong, D., Kurzrock, R., Kim, Y., Woessner, R., Younes, A., Nemunaitis, J., Fowler, N., Zhou, T., Schmidt, J., Jo, M., et al. (2015). AZD9150, a next-generation antisense oligonucleotide inhibitor of STAT3 with early evidence of clinical activity in lymphoma and lung cancer. Sci. Transl. Med. *7*, 314ra185.
23. Kortylewski, M., and Nechaev, S. (2014). Cancer therapy using oligonucleotide-based STAT3 inhibitors: will they deliver? Ther. Deliv. *5*, 239–242.
24. Iwasaki, A., and Medzhitov, R. (2004). Toll-like receptor control of the adaptive immune responses. Nat. Immunol. *5*, 987–995.
25. Krieg, A.M. (2012). CpG still rocks! Update on an accidental drug. Nucleic Acid Ther. *22*, 77–89.
26. Kortylewski, M., Swiderski, P., Herrmann, A., Wang, L., Kowolik, C., Kujawski, M., Lee, H., Scuto, A., Liu, Y., Yang, C., et al. (2009). In vivo delivery of siRNA to immune cells by conjugation to a TLR9 agonist enhances antitumor immune responses. Nat. Biotechnol. *27*, 925–932.
27. Nechaev, S., Gao, C., Moreira, D., Swiderski, P., Jozwiak, A., Kowolik, C.M., Zhou, J., Armstrong, B., Raubitschek, A., Rossi, J.J., and Kortylewski, M. (2013). Intracellular processing of immunostimulatory CpG-siRNA: Toll-like receptor 9 facilitates siRNA dicing and endosomal escape. J. Control. Release *170*, 307–315.
28. Hossain, D.M., Dos Santos, C., Zhang, Q., Kozlowska, A., Liu, H., Gao, C., Moreira, D., Swiderski, P., Jozwiak, A., Kline, J., et al. (2014). Leukemia cell-targeted STAT3 silencing and TLR9 triggering generate systemic antitumor immunity. Blood *123*, 15–25.
29. Hossain, D.M., Pal, S.K., Moreira, D.F.D., Duttagupta, P., Zhang, Q., Won, H., Jones, J., D'Apuzzo, M., Forman, S., and Kortylewski, M. (2015). TLR9-targeted STAT3 silencing abrogates immunosuppressive activity of myeloid-derived suppressor cells from prostate cancer patients. Clin. Cancer Res. *21*, 3771–3782.
30. Jahrsdorfer, B., Mühlenhoff, L., Blackwell, S.E., Wagner, M., Poeck, H., Hartmann, E., Jox, R., Giese, T., Emmerich, B., Endres, S., et al. (2005). B-cell lymphomas differ in their responsiveness to CpG oligodeoxynucleotides. Clin. Cancer Res. *11*, 1490–1499.
31. Muzio, M., Bertilaccio, M.T., Simonetti, G., Frenquelli, M., and Caligaris-Cappio, F. (2009). The role of toll-like receptors in chronic B-cell malignancies. Leuk. Lymphoma *50*, 1573–1580.
32. Wang, J.Q., Jeelall, Y.S., Beutler, B., Horikawa, K., and Goodnow, C.C. (2014). Consequences of the recurrent MYD88(L265P) somatic mutation for B cell tolerance. J. Exp. Med. *211*, 413–426.
33. Carvalho, A., Cunha, C., Almeida, A.J., Osório, N.S., Saraiva, M., Teixeira-Coelho, M., Pedreiro, S., Torrado, E., Domingues, N., Gomes-Alves, A.G., et al. (2012). The rs5743836 polymorphism in TLR9 confers a population-based increased risk of non-Hodgkin lymphoma. Genes Immun. *13*, 197–201.
34. Zhang, Q., Hossain, D.M., Duttagupta, P., Moreira, D., Zhao, X., Won, H., Buettner, R., Nechaev, S., Majka, M., Zhang, B., et al. (2016). Serum-resistant CpG-STAT3 decoy for targeting survival and immune checkpoint signaling in acute myeloid leukemia. Blood *127*, 1687–1700.
35. Leonard, J.P., Link, B.K., Emmanouilides, C., Gregory, S.A., Weisdorf, D., Andrey, J., Hainsworth, J., Sparano, J.A., Tsai, D.E., Horning, S., et al. (2007). Phase I trial of Toll-like receptor 9 agonist PF-3512676 with and following rituximab in patients with recurrent indolent and aggressive non Hodgkin's lymphoma. Clin. Cancer Res. *13*, 6168–6174.
36. Link, B.K., Ballas, Z.K., Weisdorf, D., Wooldridge, J.E., Bossler, A.D., Shannon, M., Rasmussen, W.L., Krieg, A.M., and Weiner, G.J. (2006). Oligodeoxynucleotide CpG 7909 delivered as intravenous infusion demonstrates immunologic modulation in patients with previously treated non-Hodgkin lymphoma. J. Immunother. *29*, 558–568.
37. Murray, P.J., and Smale, S.T. (2012). Restraint of inflammatory signaling by interdependent strata of negative regulatory pathways. Nat. Immunol. *13*, 916–924.
38. Kortylewski, M., Kujawski, M., Herrmann, A., Yang, C., Wang, L., Liu, Y., Salcedo, R., and Yu, H. (2009). Toll-like receptor 9 activation of signal transducer and activator of transcription 3 constrains its agonist-based immunotherapy. Cancer Res. *69*, 2497–2505.
39. Gao, C., Kozlowska, A., Nechaev, S., Li, H., Zhang, Q., Hossain, D.M., Kowolik, C.M., Chu, P., Swiderski, P., Diamond, D.J., et al. (2013). TLR9 signaling in the tumor microenvironment initiates cancer recurrence after radiotherapy. Cancer Res. *73*, 7211–7221.
40. Alas, S., and Bonavida, B. (2003). Inhibition of constitutive STAT3 activity sensitizes resistant non-Hodgkin's lymphoma and multiple myeloma to chemotherapeutic drug-mediated apoptosis. Clin. Cancer Res. *9*, 316–326.
41. Hardee, J., Ouyang, Z., Zhang, Y., Kundaje, A., Lacroute, P., and Snyder, M. (2013). STAT3 targets suggest mechanisms of aggressive tumorigenesis in diffuse large B-cell lymphoma. G3 (Bethesda) *3*, 2173–2185.
42. Xiang, M., Kim, H., Ho, V.T., Walker, S.R., Bar-Natan, M., Anahtar, M., Liu, S., Toniolo, P.A., Kroll, Y., Jones, N., et al. (2016). Gene expression-based discovery of atovaquone as a STAT3 inhibitor and anticancer agent. Blood *128*, 1845–1853.
43. Römer, P.S., Berr, S., Avota, E., Na, S.Y., Battaglia, M., ten Berge, I., Einsele, H., and Hünig, T. (2011). Preculture of PBMCs at high cell density increases sensitivity of T-cell responses, revealing cytokine release by CD28 superagonist TGN1412. Blood *118*, 6772–6782.
44. Li, J., Song, W., Czerwinski, D.K., Varghese, B., Uematsu, S., Akira, S., Krieg, A.M., and Levy, R. (2007). Lymphoma immunotherapy with CpG oligodeoxynucleotides requires TLR9 either in the host or in the tumor itself. J. Immunol. *179*, 2493–2500.
45. Jiang, W., Lederman, M.M., Harding, C.V., and Sieg, S.F. (2011). Presentation of soluble antigens to CD8+ T cells by CpG oligodeoxynucleotide-primed human naive B cells. J. Immunol. *186*, 2080–2086.
46. Betting, D.J., Yamada, R.E., Kafi, K., Said, J., van Rooijen, N., and Timmerman, J.M. (2009). Intratumoral but not systemic delivery of CpG oligodeoxynucleotide augments the efficacy of anti-CD20 monoclonal antibody therapy against B cell lymphoma. J. Immunother. *32*, 622–631.
47. Betting, D.J., Hurvitz, S.A., Steward, K.K., Yamada, R.E., Kafi, K., van Rooijen, N., and Timmerman, J.M. (2012). Combination of cyclophosphamide, rituximab, and intratumoral CpG oligodeoxynucleotide successfully eradicates established B cell lymphoma. J. Immunother. *35*, 534–543.
48. Marabelle, A., Kohrt, H., Sagiv-Barfi, I., Ajami, B., Axtell, R.C., Zhou, G., Rajapaksa, R., Green, M.R., Torchia, J., Brody, J., et al. (2013). Depleting tumor-specific Tregs at a single site eradicates disseminated tumors. J. Clin. Invest. *123*, 2447–2463.
49. Kortylewski, M., and Kuo, Y.H. (2014). Push and release: TLR9 activation plus STAT3 blockade for systemic antitumor immunity. OncoImmunology *3*, e27441.
50. Cheng, F., Wang, H.W., Cuenca, A., Huang, M., Ghansah, T., Brayer, J., Kerr, W.G., Takeda, K., Akira, S., Schoenberger, S.P., et al. (2003). A critical role for Stat3 signaling in immune tolerance. Immunity *19*, 425–436.
51. Cao, Y., Zhou, X., Zhou, M., Xu, D., Ma, Q., Zhang, P., Huang, X., Li, Q., Ma, D., and Zhou, J. (2014). Inhibition of STAT3 activity re-activates anti-tumor immunity but fails to restore the immunogenicity of tumor cells in a B-cell lymphoma model. Cancer Biol. Ther. *15*, 1153–1162.
52. Camicia, R., Winkler, H.C., and Hassa, P.O. (2015). Novel drug targets for personalized precision medicine in relapsed/refractory diffuse large B-cell lymphoma: a comprehensive review. Mol. Cancer *14*, 207.
53. Kimbrel, E.A., Davis, T.N., Bradner, J.E., and Kung, A.L. (2009). In vivo pharmacodynamic imaging of proteasome inhibition. Mol. Imaging *8*, 140–147.
54. Turkson, J., Kim, J.S., Zhang, S., Yuan, J., Huang, M., Glenn, M., Haura, E., Sebti, S., Hamilton, A.D., and Jove, R. (2004). Novel peptidomimetic inhibitors of signal transducer and activator of transcription 3 dimerization and biological activity. Mol. Cancer Ther. *3*, 261–269.

YMTHE, Volume *26*

Supplemental Information

B Cell Lymphoma Immunotherapy Using TLR9-Targeted Oligonucleotide STAT3 Inhibitors

Xingli Zhao, Zhuoran Zhang, Dayson Moreira, Yu-Lin Su, Haejung Won, Tomasz Adamus, Zhenyuan Dong, Yong Liang, Hongwei H. Yin, Piotr Swiderski, Raju K. Pillai, Larry Kwak, Stephen Forman, and Marcin Kortylewski

Supplementary Materials and Methods

Western blot for phospho-iκBα

10^6 of mouse A20 cells or human OCI-Ly3 lymphoma cells were engrafted SC into BALB/C or NSG mice, respectively. After tumors were established, mice were treated using 5 mg/kg CpG(B)-STAT3dODN, control CpG(B)-scrODN or PBS every day for 3 days. Tumors were harvested and protein was extracted for Western blot detection of phospho-iκBα (Cell signaling) levels compared to β-actin (Santa Cruz) used a s a loading control.

Animal studies

BALB/c mice with established disseminated $A20^{LUC}$ lymphoma were treated using 5 mg/kg of CpG(B)-STAT3dODN injected every other day, 200mg/kg of atovaquone (Mepron™) injected daily or PBS. Lymphoma burden was monitored using BLI for up to 100 days for surviving mice.

Luminex assays

Cytokine secretion from cultured healthy donors' PBMC was analyzed using Human Magnetic Luminex Screening Assay (R&D) on Flexmap 3D system (Luminex).

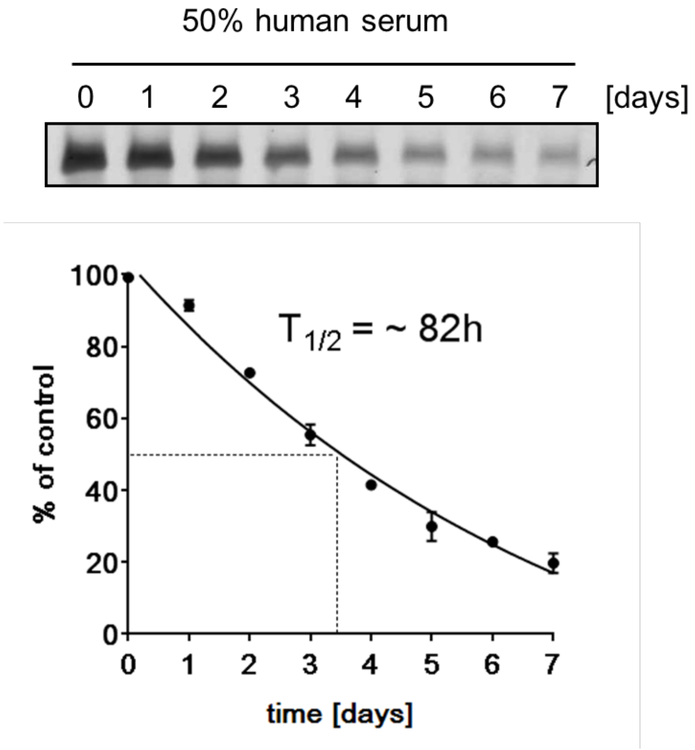

Supplemental Fig. S1. Stability of chemically modified CpG-STAT3dODN in human serum. CpG(B)-STAT3dODN were incubated in 50% human serum at 37℃ for the indicated times. The samples were then resolved on the 7.5 M Urea/20 % PAGE gel and stained using ethidium bromide; the representative gel image for CpG(B)-STAT3dODN is shown in the upper panel. Bottom panel shows the quantification of band intensities for CpG(B)-STAT3dODN; shown are means ± SEM (*n* = 3). The estimated half-life ($T_{1/2}$) of CpG(B)-STAT3dODN was indicated.

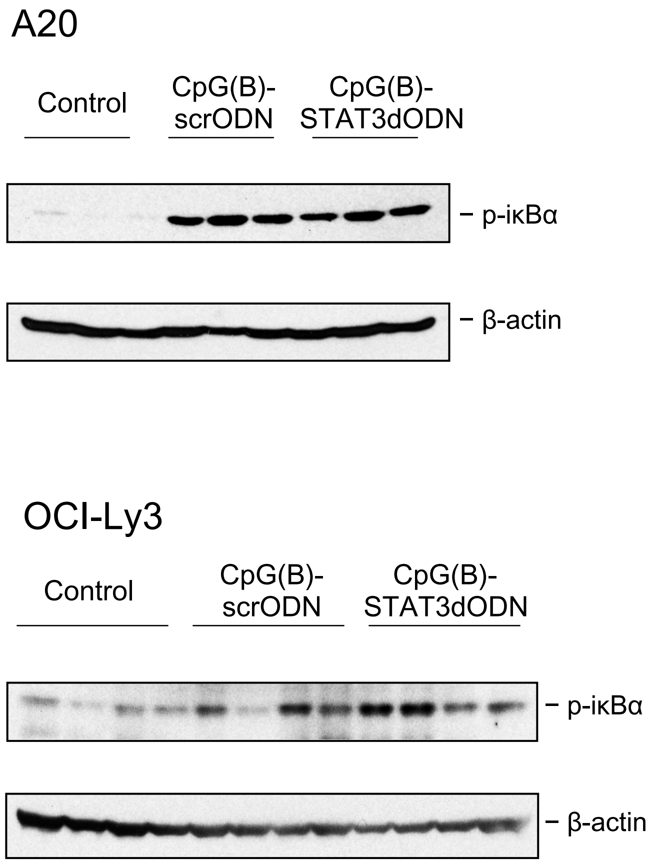

Supplemental Fig. S2. ***In vivo*** **CpG(B)-STAT3dODN treatment results in activation of NF-κB signaling.** (A) 10^6 of mouse A20 cells or human OCI-Ly3 lymphoma cells were engrafted SC into BALB/C or NSG mice, respectively. After tumors were established, mice were treated using 5 mg/kg CpG(B)-STAT3dODN, control CpG(B)-scrODN or PBS every day for 3 days. Tumors were harvested and protein was extracted for Western blot detection of phospho-iκBα levels compared to β-actin used a s a loading control.

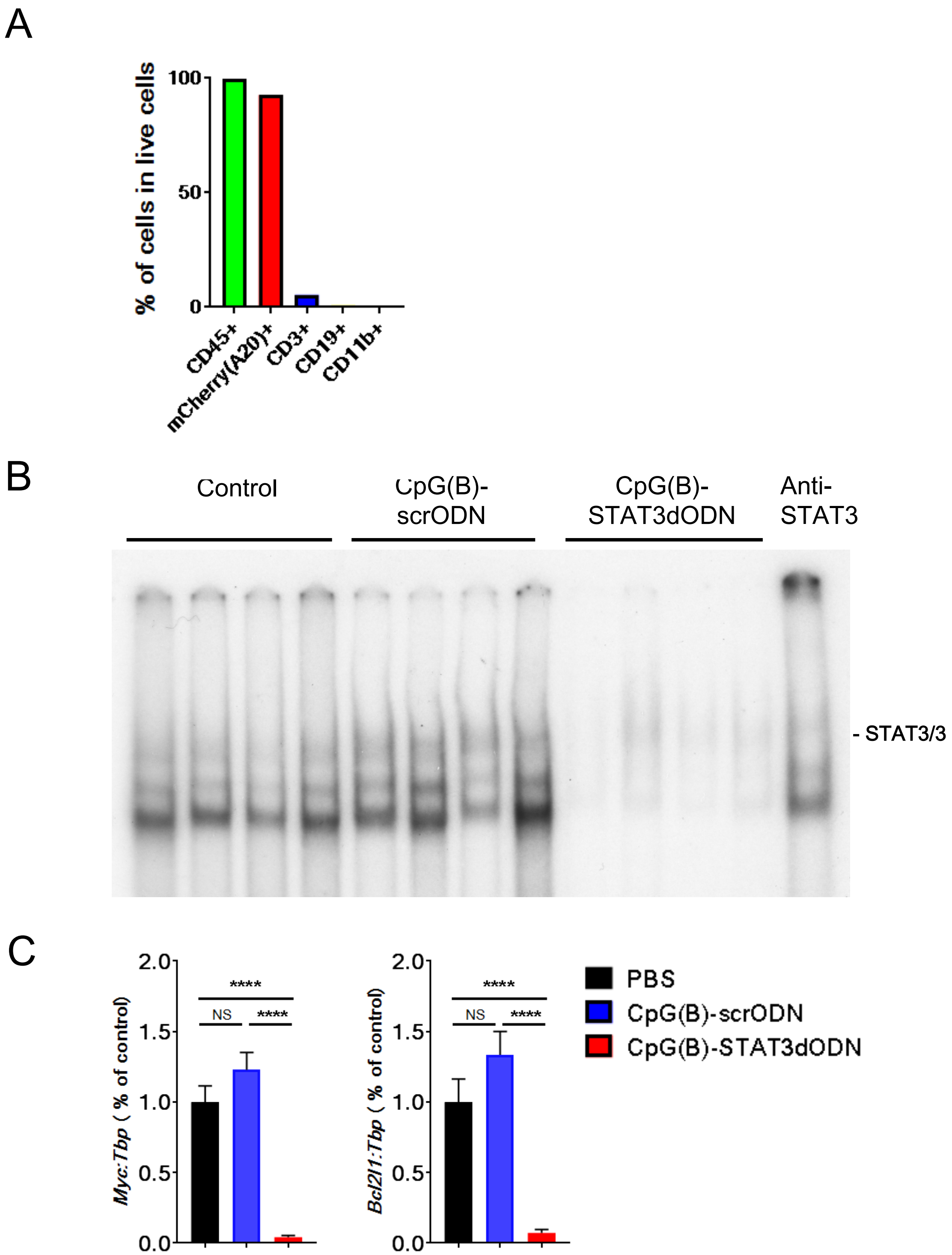

Supplemental Fig. S3. Local administration of CpG(B)-STAT3dODN inhibits STAT3 and downstream target gene expression in A20 lymphoma.

BALB/c mice were engrafted SC using 10^7 A20/mCherry/Luc cells. After tumors were established, mice treated with 1 mg/kg CpG(B)-STAT3dODN, CpG(B)-scrODN or PBS every day for 3 days. Tumors were harvested to assess cellular composition, STAT3 inhibition and downstream gene activity **(A)** Cellular composition of A20 tumors from mice before treatment as assessed using flow cytometry. Over 95% of cells are $CD45^+$/$mCherry^+$ A20 lymphoma cells with a small infiltrate of $CD3^+$ T cells and <1% of $CD45^-$ stromal cells. STAT3 activity measured using EMSA **(B)** and *c-Myc* and *Bcl2l1* expression using qPCR **(C)** after normalization to *Tbp.* Results (B,C) from one of two independent experiments; means ± SEM (n = 4).

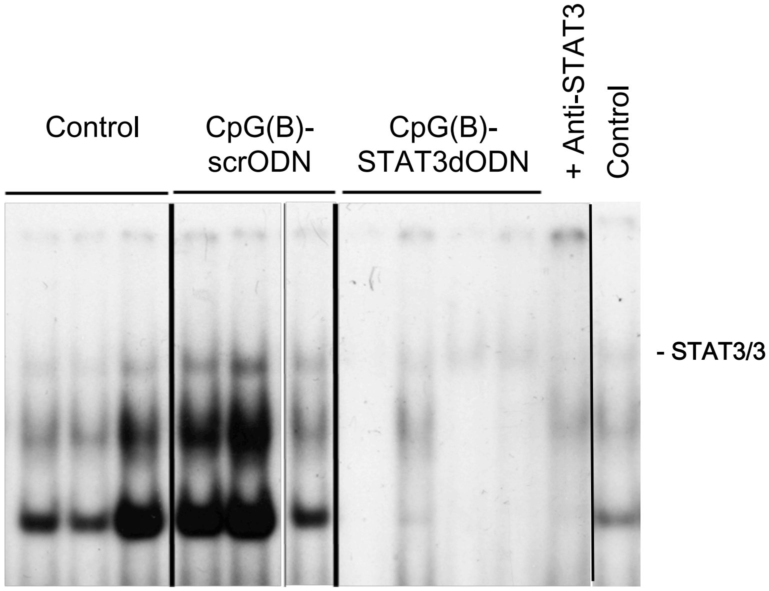

Supplemental Fig. S4. Treatment with CpG(B)-STAT3dODN inhibits STAT3 DNA binding in human B cell lymphoma *in vivo*. 10^6 of human B-cell lymphoma OCI-Ly3 cells were engrafted SC into NSG mice. After tumors were established, mice were treated using 1 mg/kg CpG(B)-STAT3dODN, control CpG(B)-scrODN or PBS every day for 3 days. Tumors were harvested for EMSA analysis of STAT3 activity using hSIE probe. The location of major STAT3-specific bands is indicated.

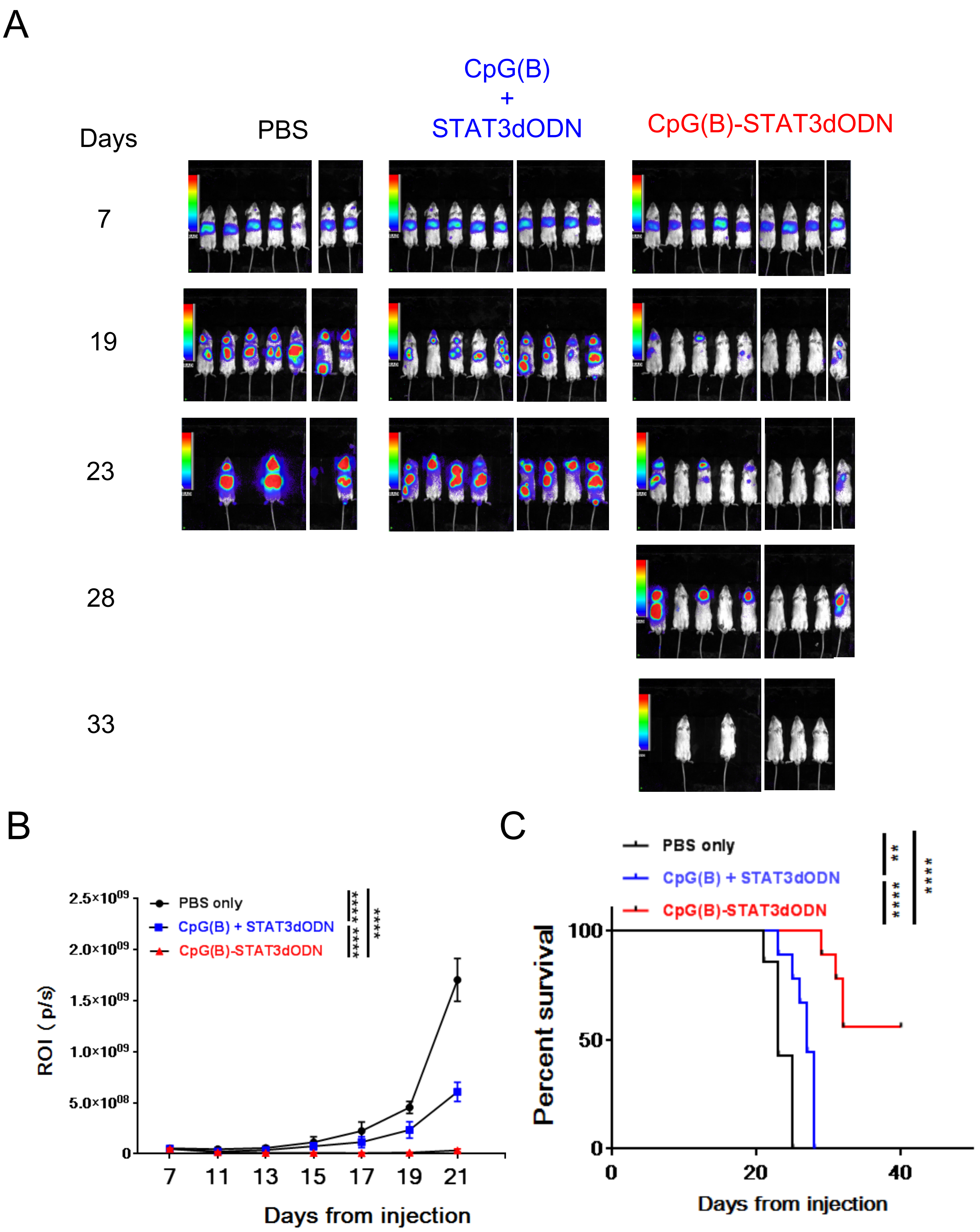

Supplemental Fig. S5. CpG(B)-STAT3dODN showed better antitumor effect *in vivo* compared with co-injection of equimolar amounts of CpG and STAT3dODN. **(A)** Mice with established systemic A20^{LUC} lymphoma were treated every other day starting from day 9 after injection using PBS or 5.3 nmole of each: CpG(B)-STAT3dODN, CpG(B) alone or together with equimolar amount of STAT3dODN alone; Lymphoma burden was monitored using BLI. **(B)** Tumor growth kinetics were assessed using quantification of the BLI signal during the experiment; ROI, regions of interest; p/s, photons/second; means ± SEM (*n* = 9/per group, except for PBS: *n* = 7). **(C)** CpG(B)-STAT3dODN but not CpG(B) plus STAT3dODN alone (*n* = 9/per group, except for PBS: *n* = 7) results in long-term survival of the majority of mice.

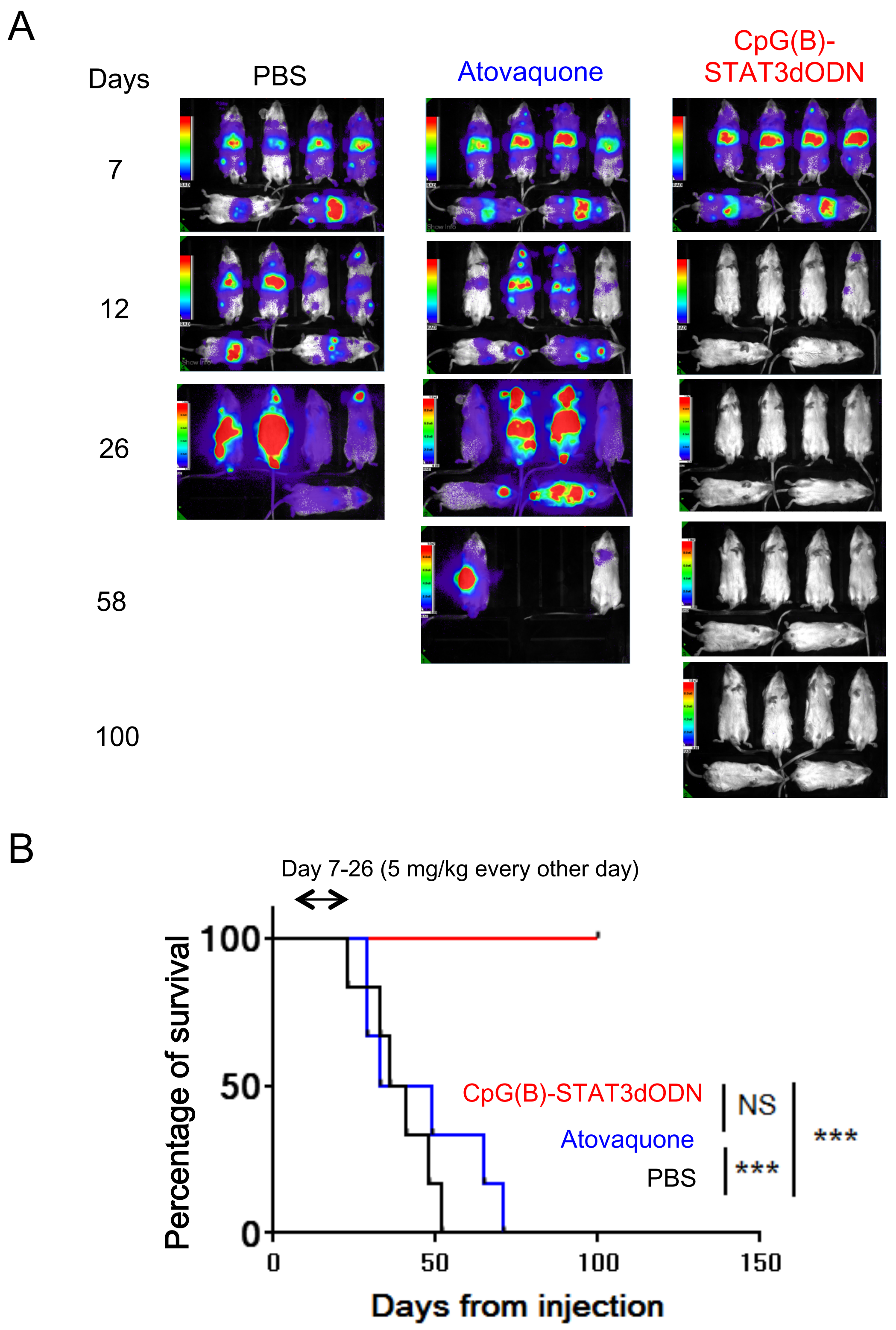

Supplemental Fig. S6. Systemic administration of CpG(B)-STAT3dODN induces better regression of syngeneic A20 B-cell lymphoma mouse model compared with the STAT3 inhibitor (atovaquone) treatment. (A) BALB/c mice with established disseminated A20^{LUC} lymphoma were treated using 5 mg/kg/every other day of CpG(B)-STAT3dODN, 200 mg/kg/day atovaquone or PBS. Lymphoma burden was monitored using BLI for up to 100 days for surviving mice. Shown are representative images from one experiments. **(B)** Treatment with CpG(B)-STAT3dODN but not atovaquone (n = 6/per group) leads to long-term mice survival. Shown are survival curves for the indicated treatment groups.

A

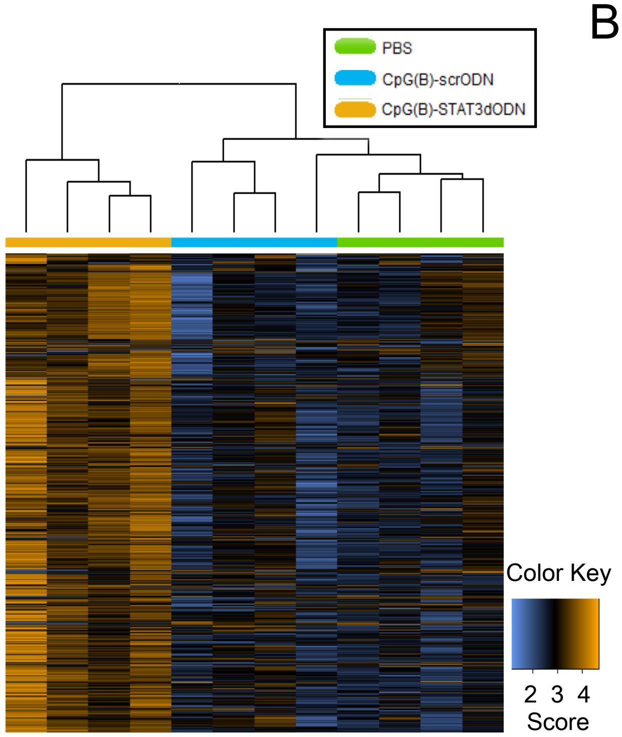

B

Signaling pathways (number of genes)	Significantly altered genes compared to PBS-treated group	
	CpG-scrODN (% coverage)	CpG-STAT3dODN (% coverage)
Cell adhesion (28)	7.1	71.4
NF-κB signaling (91)	2.2	33.0
PI3K-AKT signaling (30)	10.0	66.7
Apoptosis (45)	4.4	53.3
T-cell receptor signaling (37)	5.4	43.2
TNF signaling (63)	1.6	54.0

C

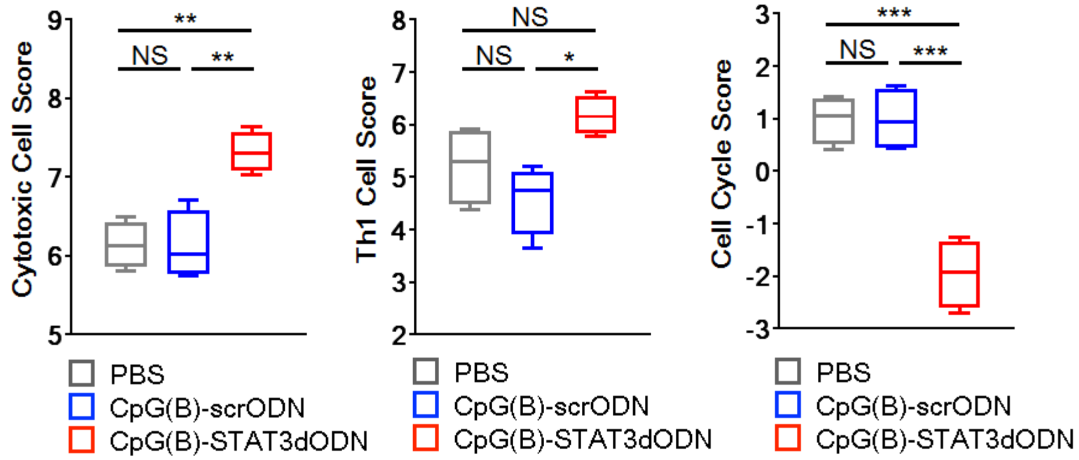

Supplemental Fig. S7. Gene expression profiling of B-cell lymphoma following TLR9 stimulation with or without concurrent STAT3 inhibition. Mice with established A20 lymphoma (SC) were treated starting on day 14 after tumor challenge using 1 mg/kg of CpG(B)-STAT3dODN, control CpG(B)-scrODN or PBS three times every other day (*n* = 4/per each group). Before tumor volumes differed, tumors were harvested to isolate total RNA for Nanostring gene expression analysis. **(A)** Comparative heat-map analysis of total gene expression changes in all samples. **(B)** Top functional pathways operating in CpG(B)-STAT3dODN- and control CpG(B)-scrODN-treated samples vs. PBS-treated controls. **(C)** Predictive scores generated by the nSolver analysis for the presence of cytotoxic cells (left), Th1 cells (middle) and the active cell cycle (right) in tested B-cell lymphoma samples; shown are means ± SEM (*n* = 4).

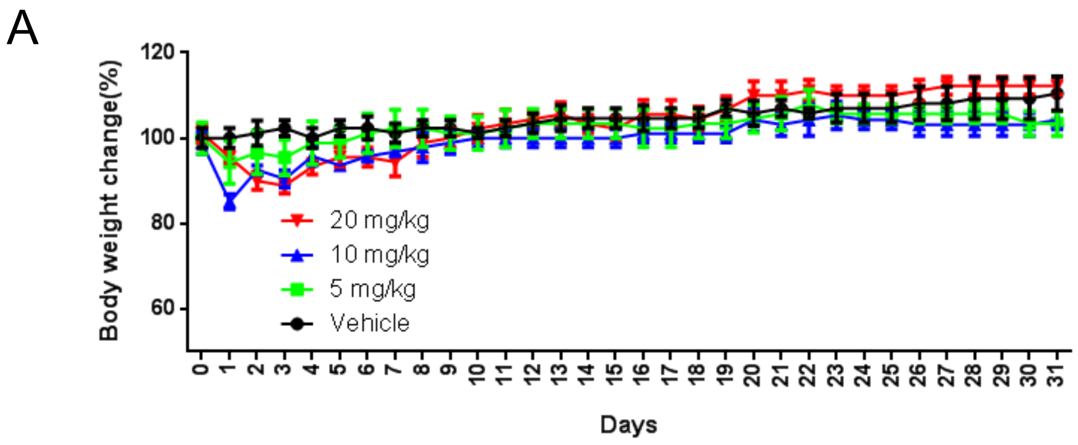

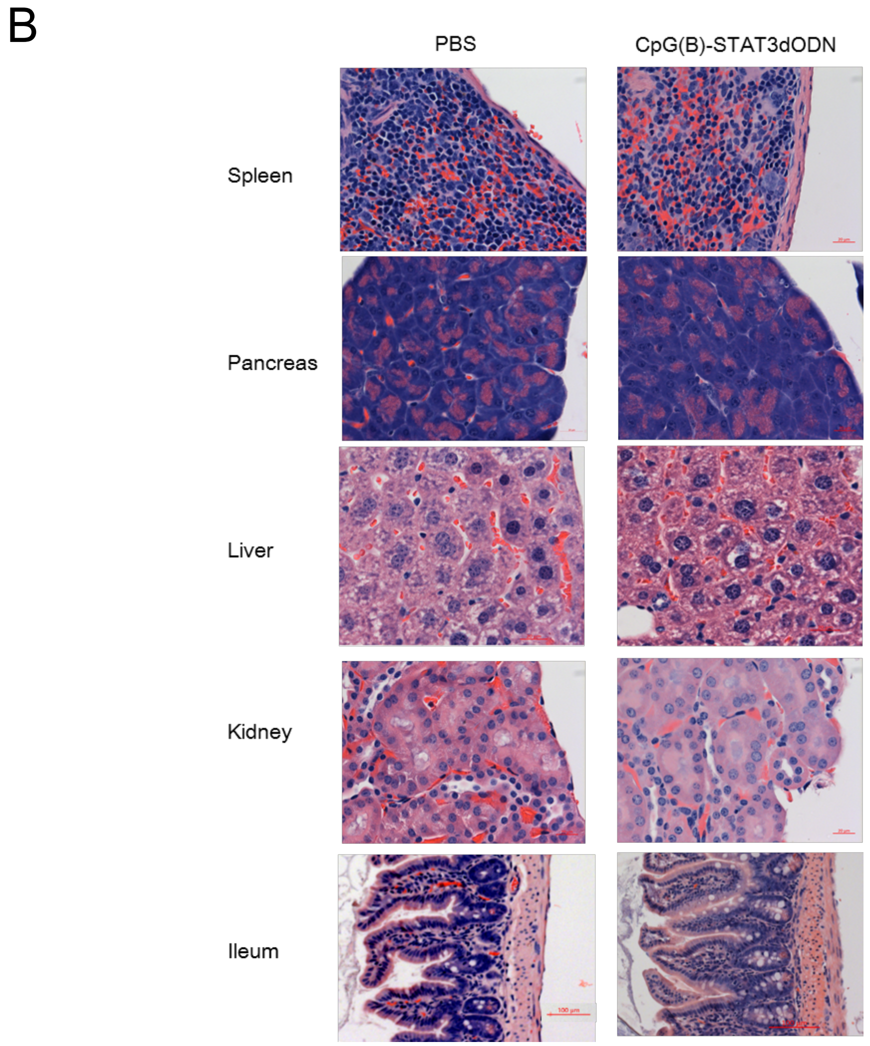

Supplemental Fig. S8. Repeated injection of CpG(B)-STAT3dODN are well tolerated in mice. (A) Balb/C mice were injected IV with 5, 10, or 20 mg/kg of CpG-STAT3dODN every other day for two weeks. The animal condition was monitored by changes in body weight compared to animal weight at the baseline (day 0) set as 100%. There were no toxicities or mortalities during the treatment and for up to 2 weeks after treatment completions. Shown are means ± SD (*n* = 4). **(B)** Repeated CpG(B)-STAT3dODN treatments did not result in gross abnormalities or inflammatory manifestations in tested organs as assessed 2 weeks after treatment completion. Histological assessment was performed on tissue sections from the highest dose group (60 mg/kg/week) vs. PBS control group. Tissue sections from the indicated organs were formalin fixed and stained using haematoxylin and eosin.

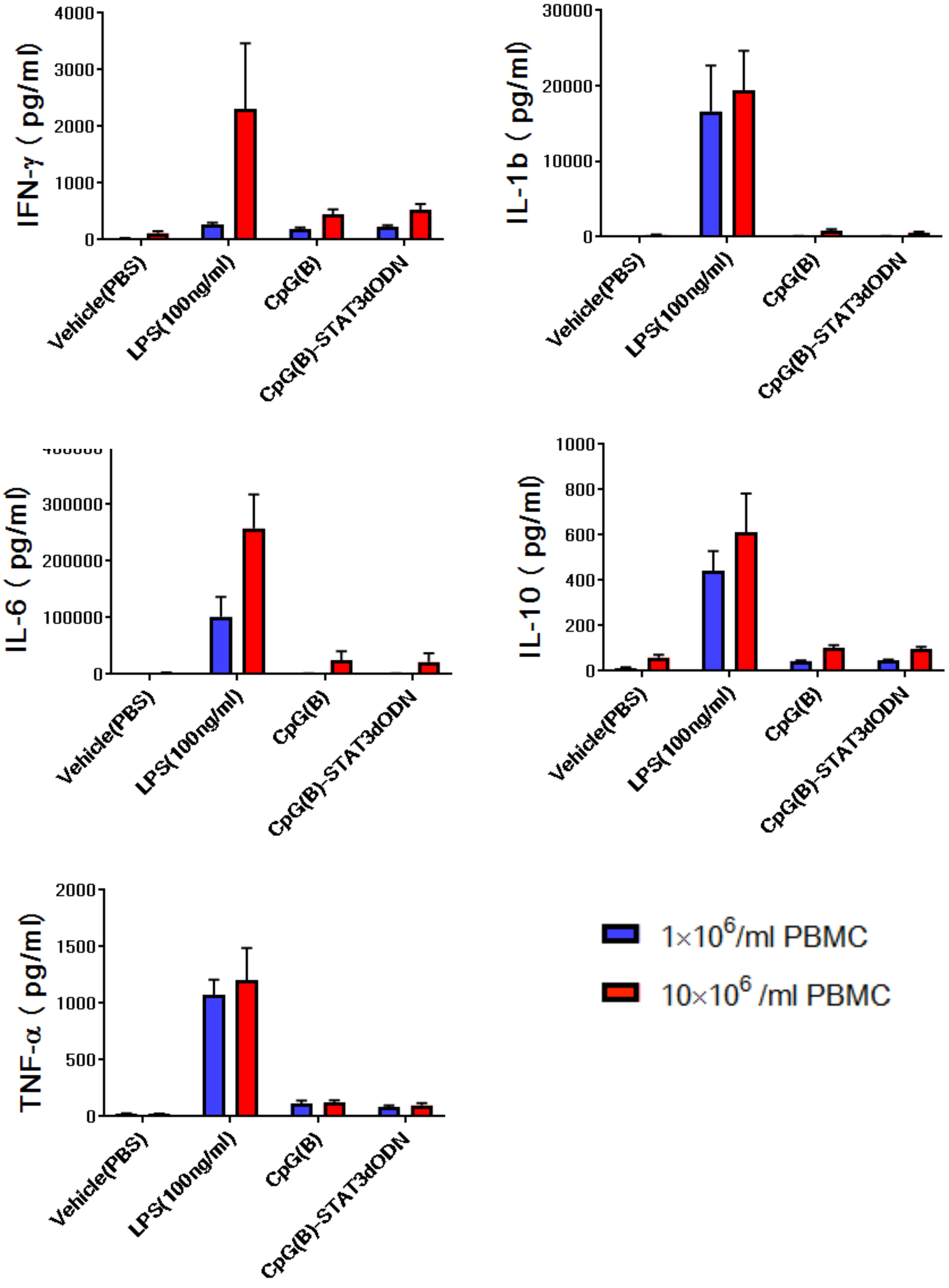

Supplemental Fig. S9. Proinflammatory cytokine production by human PBMCs cultured at low or high cell density in the presence of CpG(B)-STAT3dODN. Human PBMC isolated from from 9 healthy donors were cultured in RPMI 1640 medium (10% FBS) at standard or high density, 1 or 10 mln cells/ml, respectively. Cell were then treated using PBS, LPS (100 ng/ml), CpG(B) alone (1 μM), CpG(B)-STAT3dODN (1 μM) for 48 hours. Supernatant were collected and concentrations of immunoregulatory mediators was analyzed using Luminex assay; shown are means ± SEM (*n* = 9).
